# Recurrent evolution of selfishness from an essential tRNA synthetase in *Caenorhabditis tropicalis*

**DOI:** 10.1038/s41559-025-02894-2

**Published:** 2025-11-17

**Authors:** Polina Tikanova, James Julian Ross, Andreas Hagmüller, Florian Pühringer, Pinelopi Pliota, Daniel Krogull, Valeria Stefania, Manuel Hunold, Alevtina Koreshova, Anja Koller, Ivanna Ostapchuk, Jacqueline Okweri, Joseph Gokcezade, Peter Duchek, Gang Dong, Eyal Ben-David, Alejandro Burga

**Affiliations:** 1 Institute of Molecular Biotechnology of the Austrian Academy of Sciences, Vienna BioCenter, Vienna, Austria; 2 Vienna BioCenter PhD Program, Doctoral School of the University of Vienna and Medical University of Vienna, Vienna, Austria; 3 Max Perutz Labs, Vienna BioCenter, Medical University of Vienna, Vienna, Austria; 4 Illumina Artificial Intelligence Laboratory, Illumina, San Diego, CA USA

**Keywords:** Molecular evolution, Evolutionary biology

## Abstract

No genome on Earth is free of selfish genes. This reflects both their ability to subvert the laws of inheritance and their de novo emergence from host genes. Yet, despite their ubiquity and key role in driving innovation, the mechanisms responsible for their genesis remain largely unexplored. Here we report the discovery of three toxin–antidote elements in the nematode *Caenorhabditis tropicalis*. Toxin–antidote elements are selfish genes that increase their frequency in populations by poisoning non-carrier individuals. We find that all three novel toxins—*klmt-1*, *pzl-1* and *hyde-1*—arose via gene duplication from *fars-3*, an essential subunit of the phenylalanyl tRNA synthetase. Their antidotes—KSS proteins—are rapidly evolving F-box proteins that degrade toxins via the SCF ubiquitin–ligase complex. Our phylogenetic and genomic analyses strongly suggest that the ancestor of all extant KSS antidotes fortuitously acquired affinity for FARS-3, much like ‘self’ proteins are targeted in autoimmune disease. This interaction neutralized the toxicity of future paralogues before it arose (presuppression), allowing otherwise deleterious mutant alleles to persist and ultimately evolve into selfish genes—consistent with the theory of constructive neutral evolution.

## Abstract (web summary)

In *Caenorhabditis tropicalis*, three toxin–antidote elements arose via gene duplication from the essential tRNA-synthetase subunit FARS-3. The ancestral antidote probably acquired affinity for FARS-3, enabling presuppression of toxicity and allowing otherwise deleterious mutant alleles to evolve into selfish genes.

## Main

Selfish elements are the cellular currency of innovation. Much like wealth, they are primarily passed down through generations, occasionally exchanged and exceptionally forged anew. Yet, regardless of their age or unexpected origins—such as horizontal gene transfer^1^—all selfish elements must ultimately trace back their origins to genes that were once non-selfish. The exact mechanisms behind this transformation remain elusive, leaving critical gaps in our understanding of genome evolution and the emergence of biological novelty.

**Fig. 1:**
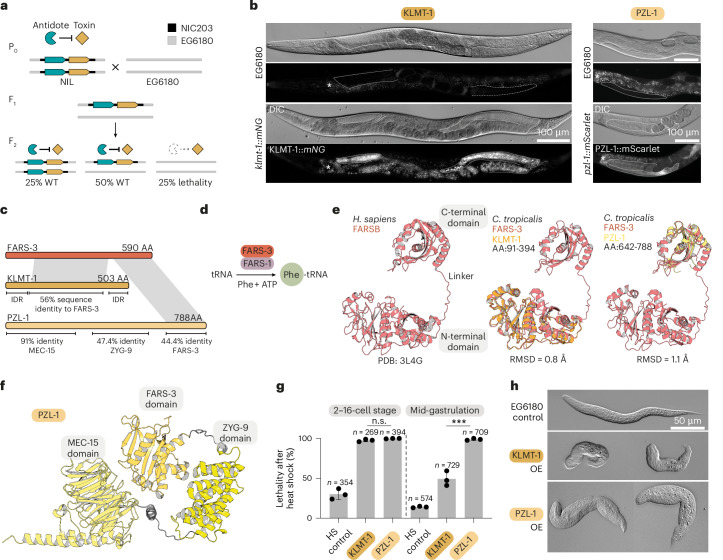
Two toxins, PZL-1 and KLMT-1, evolved from FARS-3, an essential tRNA synthetase subunit. **a**, TA mechanism of action. In crosses between TA-carrying NILs (Chr. II or Chr. V NIC203 introgression into EG6180 background) and the susceptible strain (EG6180), all F_2_ progeny are exposed to a maternally deposited toxin. Homozygous susceptible individuals (25% of the F_2_) lack the TA locus and are affected because they cannot express the zygotic antidote. NIC203 introgression is shown in black. **b**, Expression of *klmt-1::mNG*, or *pzl-1::mScarlet* and corresponding differential interference contrast images. *mNeonGreen* (*mNG*). Both proteins localize to the gonads (dotted outline), unfertilized eggs and embryos. The signal in the gut (asterisk) corresponds to autofluorescence, also detectable in the EG6180 control. **c**, FARS-3, KLMT-1 (for killer of embryos and larvae maternal toxin) and PZL-1 (PheRS beta subunit, z*yg-9* and *mec-15* fusion maternal toxin) protein domains and shared homology. IDR, intrinsically disordered region. **d**, FARS-3 and FARS-1 form the phenylalanyl–tRNA synthetase complex. **e**, Comparison of *Homo sapiens* FARSB structure (left) with the predicted structure of *C. tropicalis* FARS-3, overlaid with KLMT-1 (middle) or PZL-1 (right) predictions. KLMT-1 does not include the predicted N- and C-terminal IDRs; PZL-1 includes only the C-terminal region with homology to FARS-3. **f**, AlphaFold2 model of the full-length PZL-1. **g**, Overexpression of KLMT-1 and PZL-1 under a heat-shock-inducible promoter in early embryos and after 3 h of development. Both kill early embryos (two-sided unpaired *t*-test; *P* = 0.0716); however, KLMT-1 is less potent at later stages (two-sided unpaired *t*-test; *P* = 0.0008). Heat shock in EG6180 control causes ~30% background lethality. Dots indicate individual replicates; ≥50 embryos per replicate. Error bars represent the mean ± s.d. For this and following figures ***P ≤ 0.001, ****P ≤ 0.0001; n.s., not significant. **h**, Overexpression phenotypes in L1 larvae in early (up to 16-cell stage) embryos and EG6180 control. Source data

A major barrier remains the intense evolutionary pressure these elements face. The relentless arms race between selfish genes and host defence systems drives them to evolve at a much faster pace than the rest of the genome, often obscuring the details of their origins. This is particularly true for transposable elements, the best-characterized selfish genes, whose core enzymatic machinery predates the emergence of eukaryotes^2^. To overcome this limitation, we focused instead on a second class of selfish genes: toxin–antidote elements (TAs). Unlike ancient and broadly distributed transposons, TAs are phylogenetically restricted, underscoring their recent and independent origins in a diverse array of fungus, plant and animal species^3–6^. TAs typically consist of a toxin in linkage with its antidote and spread by selectively killing non-carrier offspring. In nematodes, for instance, heterozygous mothers deposit toxin into all unfertilized eggs, but only those inheriting the TA can neutralize it via zygotic antidote expression—eliminating up to 25% of F_2_ progeny and driving the element towards fixation (Fig. 1a).

However, the evolution of these genetic parasites from host genomes poses an evolutionary conundrum. Because the toxin is lethal without its antidote, and the antidote appears to serve no purpose other than neutralizing the toxin, this raises the question: how can such a selfish system originate in the first place? Natural selection would seemingly eliminate the toxin if it arose alone, and the simultaneous emergence of both components appears highly unlikely. Here, we address this conundrum by dissecting how an enzyme that is universally essential for life—the phenylalanyl–tRNA synthetase—gave birth to three distinct selfish TAs in the nematode *Caenorhabditis tropicalis*.

## Results

### Evolution of selfishness from an essential tRNA synthetase

**Extended Data Fig. 1:**
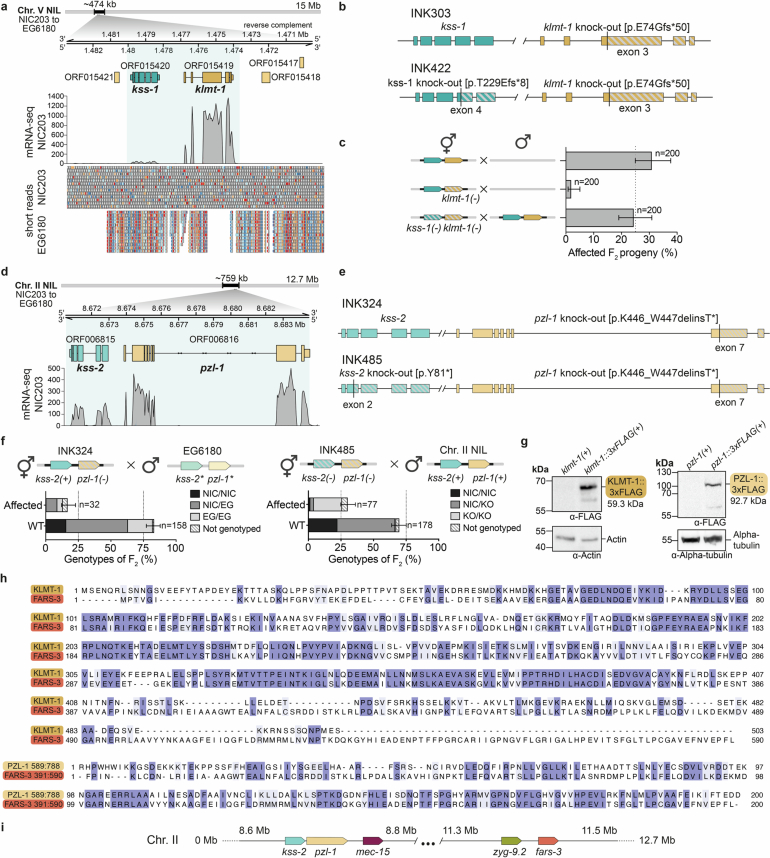
Genetic mapping of the *klmt-1/kss-1* and *pzl-1/kss-2* toxin-antidote elements. (**a**) Fine-mapping of the Chr. V TA. Alignment of genomic short reads and mRNA-seq from early embryos to the NIC203 *de novo* assembly. Top candidate genes are highlighted in light blue. (**b**) CRISPR/Cas generated *klmt-1(-)* and *klmt-1(-) kss-1(-)* alleles. (**c**) The Chr. V TA causes ~ 25% F_2_ lethality (top). *klmt-1* frameshift abolishes lethality (middle). The *kss-1(-) klmt-1(-)* double mutant is poisoned by the WT allele (bottom). Error bars represent the mean with 95% CI, hybrid Wilson/Brown method. (**d**) Fine-mapping of the Chr. II TA. (**e**) CRISPR/Cas generated *pzl-1(-)* and *pzl-1(-) kss-2(-)* alleles. (**f**) *pzl-1* frameshift abolishes F_2_ developmental delay (left). A *pzl-1(-) kss-2(-)* double mutant is poisoned by the WT allele (right). Sample sizes are shown for each phenotypic class. Error bars represent the mean with 95% CI, hybrid Wilson/Brown method. (**g**) Western blot of endogenously tagged KLMT-1::3xFLAG and PZL-1::3xFLAG. Respective NILs – negative control. (**h**) Protein sequence alignment of KLMT-1 and PZL-1 with FARS-3 (only C-terminus of PZL-1 is included). (**i**) Relative position of *mec-15*, *zyg-9*, *fars-3* and *pzl-1* on Chr. II. Source data

TAs cause extensive genetic incompatibilities in the nematode *C. tropicalis* and may have driven the evolution of self-fertilization in this species^5,7^. Through studying crosses between two wild isolates from the Caribbean, NIC203 (Guadeloupe) and EG6180 (Puerto Rico), we discovered three TAs in NIC203, located on chromosomes (Chr.) II, III and V, respectively^5^. Each of these TAs poisons individuals that are homozygous for the corresponding EG6180-susceptible allele (Fig. 1a). We previously identified the genes that comprise the Chr. III TA—*slow-1/grow-1*^8^. However, the genes underlying the Chr. II and Chr. V TAs remained unknown. To identify these genes, we leveraged near-isogenic lines (NILs), Nanopore long-read genome assemblies and RNA sequencing (RNA-seq) expression data and validated candidates using clustered regularly interspaced short palindromic repeats (CRISPR)–Cas editing. This approach allowed us to successfully map both genetic incompatibilities to single toxin–antidote gene pairs, which we named *pzl-1/kss-2* (Chr. II) and *klmt-1/kss-1* (Chr. V) (Methods; Extended Data Fig. 1a–f, Supplementary Note and Supplementary Fig. 1).

*pzl-1* (pronounced *puzzle*) and *klmt-1* (pronounced *klimt*) encode 90-kDa and 57-kDa proteins, respectively, which are maternally loaded into eggs (Fig. 1b, Extended Data Fig. 1g, Supplementary Note and Supplementary Figs. 2 and 3). At first glance, the two toxins, KLMT-1 and PZL-1, appeared unrelated, as they shared no sequence similarity. However, upon closer analysis, we were surprised to find that they were both partially homologous to FARS-3, an essential subunit of the phenylalanyl tRNA synthetase (PheRS) (Fig. 1c and Extended Data Fig. 1h). In bacteria, archaea and eukaryotes, PheRS is a heterotetrameric enzyme made up of two alpha and two beta subunits, which in nematodes are encoded by *fars-1* and *fars-3*, respectively^9,10^. PheRS plays an essential role in mRNA translation by charging tRNA^Phe^ with its cognate amino acid, L-phenylalanine (Fig. 1d). The protein architecture of FARS-3 is highly conserved between nematodes and humans and consists of an N-terminal (amino acids (AAs): 1–376) and a C-terminal domain (AAs: 390–590) connected by a small linker^9^ (Fig. 1e). Alphafold2 models suggested that KLMT-1 shares extensive structural homology with the N-terminal domain of FARS-3, while PZL-1 is homologous to the C-terminal domain (Fig. 1e). In addition, we found that PZL-1 is a chimeric toxin, exhibiting sequence and structural similarity with two other proteins, MEC-15 and ZYG-9, probably resulting from gene fusion (Fig. 1f and Extended Data Fig. 1i). This non-overlapping homology pattern and divergent domain architecture suggests distinct mechanisms of action for these toxins. Supporting this view, KLMT-1 overexpression was lethal only when induced during early embryonic stages, while PZL-1 overexpression was lethal throughout both embryonic and early larval (L1/L2) stages (Fig. 1g). Furthermore, the resulting phenotypes were markedly different: KLMT-1 poisoning resulted in small, crumpled L1 larvae, whereas PZL-1 poisoning resulted in bloated L1 larvae (Fig. 1h). We conclude that *klmt-1*/*kss-1* and *pzl-1*/*kss-2* are two evolutionarily related yet independently acting selfish elements segregating in wild populations.

### The KSS-1 and KSS-2 antidotes are closely related F-box proteins

**Fig. 2:**
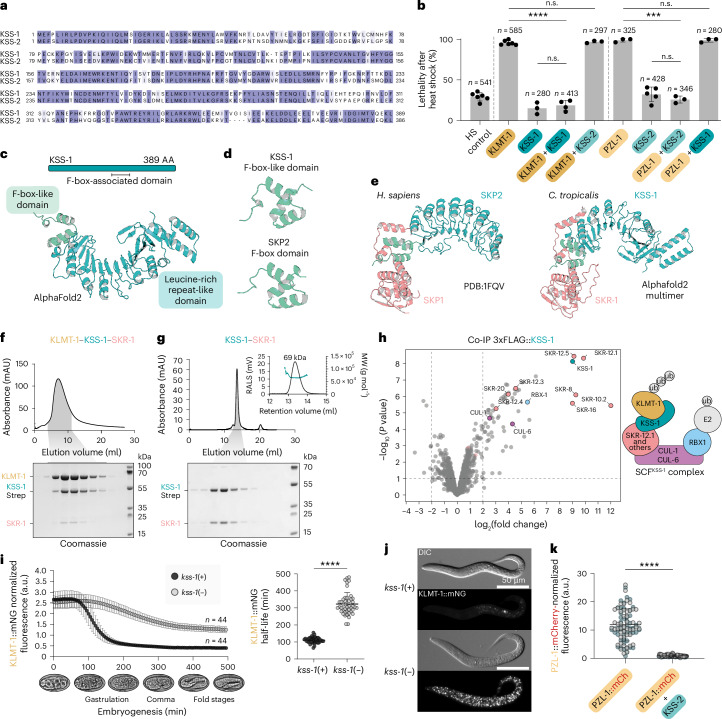
KSS-1 and KSS-2 are F-box proteins that mediate the degradation of cognate toxins. **a**, Protein sequence alignment of antidotes. **b**, Rescue assay demonstrating antidote specificity. Only cognate antidotes conferred protection (KLMT-1 with KSS-1: *P*_adj_ < 0.0001; PZL-1 with KSS-2: *P*_adj_ = 0.0003; KLMT-1 with KSS-2: *P*_adj_ = 0.7529; PZL-1 with KSS-1: *P*_adj_ > 0.9999). Overexpression of antidotes alone had no effect (KSS-1: *P*_adj_ = 0.8726, KSS-2: *P*_adj_ = 0.4825). Brown–Forsythe ANOVA followed by Dunnett post hoc test (KLMT-1 comparisons – F*(3, 5.154) = 314.6, *P* < 0.0001; PZL-1 – F*(3, 6.886) = 222.1, *P* < 0.0001); dots indicate individual replicates; ≥50 embryos per replicate. Error bars represent the mean ± s.d. n.s., not significant. **c**, Predicted KSS-1 domains and AlphaFold2 model. **d**, Comparison of human SKP2 F-box domain and KSS-1 F-box-like domain. **e**, Human SKP1–SKP2 complex and predicted *C. tropicalis* SKR-1–KSS-1 complex. **f**, Top: Strep-affinity purification of KLMT-1–KSS-1::Strep–SKR-1 complex in Sf9 cells. Bottom: elution fractions were confirmed by SDS–PAGE. **g**, Size-exclusion chromatography elution profile of the KSS-1–SKR-1 complex (top) and SDS–PAGE validation (bottom). The molecular weight (69 kDa, determined by static light scattering) matches the KSS-1–SKR-1 1:1 heterodimer (47.1 + 19.8 kDa) (inset). RALS, right-angle light scattering; MW, molecular weight. **h**, Left: volcano plot of 3xFLAG::KSS-1 co-IP/MS. Right: SCF complex members are colour-coded by function. Co-IPs performed in biological triplicates. Chr. V NIL – control. **i**, KLMT-1::mNG quantification. Left: fluorescence measured from 8–16-cell stages onwards and normalized to the *kss-1*(+) strain. Right: toxin half-life is increased in *kss-1*(−) compared with *kss-1*(+) (two-sided Mann–Whitney test, *P* < 0.0001). Error bars represent the mean ± s.d. **j**, KLMT-1::mNG localization in the L1 larvae. Top: in the *kss-1*(+) strain, localization is restricted to Z2/Z3 germline precursors, consistent with zygotic expression. Bottom: the *kss-1*(−) strain showed high KLMT-1::mNG levels. DIC, differential interference contrast. **k**, KSS-2 overexpression reduced PZL-1::mCherry expression (two-sided unpaired Welch’s *t*-test; *P* < 0.0001). Experiment repeated twice; ≥45 embryos per condition per replicate; biological replicates shown in grey and cyan. Error bars represent the mean ± s.d. Source data

The evolutionary connection between *pzl-1*/*kss-2* and *klmt-1*/*kss-1* TAs extends beyond *fars-3*, as their KSS antidotes (killer rescue via zygotically expressed gene, pronounced kiss) were also homologous. KSS-1 and KSS-2 share 64,6% sequence identity at the protein level (Fig. 2a). While very similar, KSS-1 and KSS-2 are highly specific. In controlled transgenic overexpression experiments, each antidote efficiently neutralized its cognate toxin but provided no cross-resistance (Fig. 2b). In prokaryotes, antitoxins utilize various mechanisms to neutralize toxins. For instance, some antitoxins are antisense RNAs that block toxin synthesis, while others are proteins that bind to the toxin, preventing it from interacting with its target^11,12^. Because the mechanisms by which eukaryotic antidotes neutralize toxins are still largely unexplored^13,14^, we investigated how KSS-1 and KSS-2 carry out this function.

**Extended Data Fig. 2:**
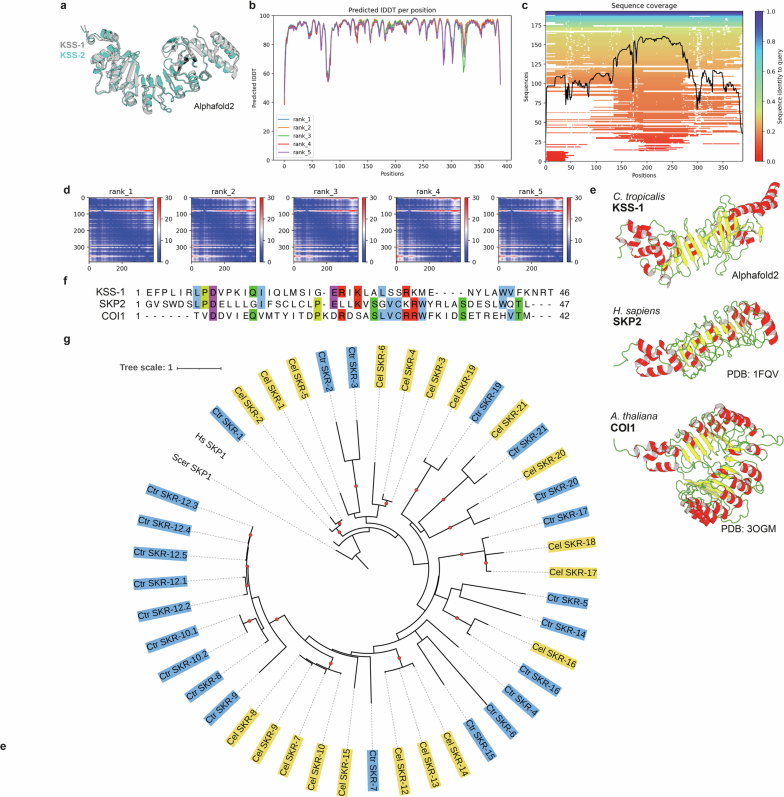
Alphafold2 predicts that KSS-1 is structurally similar to F-box proteins. (**a**) Alignment of KSS-1 (gray) and KSS-2 (cyan) Alphafold2 models (RMSD 1.6 Å). (**b**) Per-residue confidence scores (pLDDT) for the KSS-1 model. (**c**) Coverage of the Alphafold2 multiple sequence alignment. (**d**) Predicted aligned error (confidence in the domain packing and large-scale topology) for the best five KSS-1 models. (**e**) Structural comparison of the KSS-1 model with the two most similar experimentally solved structures, as identified by the DALI server: human F-box protein SKP2 (PDB: 1FQV) and Arabidopsis F-box protein COI1 (PDB: 3OGM). Alpha-helices are shown in red and beta strands in yellow. (**f**) Alignment of KSS-1, SKP2 and COI1 F-box domains. (**g**) Phylogeny of SKR proteins from *C. elegans* (yellow) and *C. tropicalis* (blue). Human SKP1 and *Saccharomyces cerevisiae* SKP1 are included as outgroups. Red circles indicate bootstrap values greater than 95%.

To better understand the molecular role of the antidotes, we predicted their tertiary structure using AlphaFold2. The resulting models showed high accuracy and revealed KSS-1 and KSS-2 are highly similar (root mean square deviation (RMSD) 1.6 Å; Extended Data Fig. 2a). Both antidotes are predicted to adopt a horseshoe fold with parallel beta sheets running on its inner face and alpha helices on its exterior (Extended Data Fig. 2a–d). Due to their structural similarity and shared molecular role, we focused on KSS-1 for further analysis. Using the KSS-1 Alphafold2 model as a template, we searched for structurally similar proteins in the Protein Data Bank (PDB)^15^. This analysis revealed significant structural similarity between KSS-1 and F-box proteins, notably human S-phase kinase-associated protein 2 (SKP2; *z*score 7.8) and *Arabidopsis* coronatine-insensitive protein 1 (COI1; *z*score 8.4). This similarity encompassed not only the C-terminal solenoid but also, crucially, the N-terminal F-box domain, despite a lack of sequence conservation (Fig. 2c,d and Extended Data Fig. 2e,f). F-box proteins are the substrate recognition subunits of the SKP1–CUL1–F-box (SCF) E3 ubiquitin ligase complexes, which target proteins for proteasome-mediated degradation.

**Extended Data Fig. 3:**
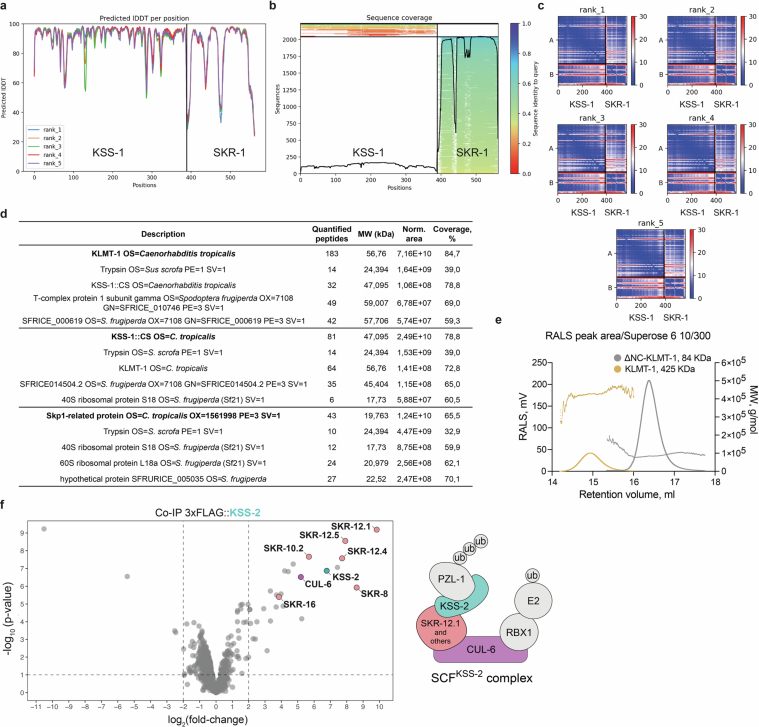
Characterization of KSS-1 and SKR-1 protein binding, and KSS-2 interactome. (**a**) Per-residue confidence indicated by the pLDDT (predicted local distance difference test) metric for the AlphaFold2-multimer model of KSS-1–SKR-1 binding. (**b**) Coverage of the multiple sequence alignment for the KSS-1 and SKR-1 interaction. (**c**) Predicted aligned error for the top five KSS-1–SKR-1 models. (**d**) LC-MS analysis of bands corresponding to KLMT-1, KSS-1::Strep and SKR-1 (shown in Fig. 2f). Five proteins with the highest normalized area for each band are listed. (**e**) Static Light Scattering (SLS) of full-length KLMT-1 and ΔNC-KLMT-1 (lacking both predicted N- and C-terminal IDRs), both expressed in *E. coli*. Full-length KLMT-1 exhibits an apparent molecular weight of ~ 425 kDa and is prone to aggregation; ΔNC-KLMT-1 is ~ 84 kDa and does not aggregate, suggesting it forms a homodimer. (**f**) Volcano plot of 3xFLAG::KSS-2 co-IP/MS (left). Construct is expressed under a constitutive promoter (*Ctr*-*rpl-36p*). Members of the SCF complex are color-coded according to their function (right). Co-IPs were performed in biological triplicates. Lysate from Chr. II NIL served as a control.

SKP1 serves as a key adaptor that links different F-box proteins to the scaffold protein CUL1, which together with RBX1 recruits the E2 ubiquitin-conjugating enzyme^16,17^. While humans and yeast possess a single *SKP1* gene, nematodes have undergone a substantial expansion of this protein family. For instance, the genomes of *Caenorhabditis* *elegans* and *C. tropicalis* code for 20 and 23 SKP1-related (*skr*) genes, respectively^18,19^ (Extended Data Fig. 2g). Among these, *skr-1* is essential for embryonic development in *C. elegans*^18–20^. We used AlphaFold-Multimer to test whether *C. tropicalis* SKR-1 interacted with the antidote. We identified a high-confidence interaction between the C terminus of SKR-1 (alpha helices H5–H8) and the N-terminal F-box-like domain of KSS-1, analogous to the interaction found in SKP1–SKP2 and other known SCF complexes^16–19,21^ (Fig. 2e and Extended Data Fig. 3a–c).

### KSS-1 binds to KLMT-1 and targets it for degradation via the SCF complex

The structural similarity between KSS-1 and F-box proteins led us to hypothesize that KSS-1 targets KLMT-1 for degradation by binding the toxin and recruiting the SCF complex. To test this model in vitro, we co-expressed a C-terminally tagged KSS-1::Strep together with untagged versions of KLMT-1 and SKR-1 in Sf9 insect cells. Affinity purification of KSS-1::Strep followed by size-exclusion chromatography revealed that KSS-1 stably binds both KLMT-1 and SKR-1 (Fig. 2f and Extended Data Fig. 3d). KLMT-1’s strong tendency to aggregate through its disordered regions prevented us from identifying the precise stoichiometry of this complex (Extended Data Fig. 3e); however, in agreement with SKP1–SKP2 structures^21^, we found that KSS-1 and SKR-1 form a stable 1:1 heterodimer (Fig. 2g).

To test whether KSS-1 binds components of the SCF complex in vivo, we performed co-immunoprecipitation of an N-terminally tagged 3xFLAG::KSS-1 transgene followed by quantitative mass spectrometry (co-IP/MS). As expected, KSS-1 was highly enriched in the IP samples compared with a wild-type (WT) control line (~500 fold change; *P* < 10^−8^; Fig. 2h). We also identified eight SKR proteins among the most significant KSS-1 interactors, many of which were closely related paralogues, such as SKR-10.2, SKR-12.1 and SKR-12.5 (~500–4,000 fold change; *P* < 10^−5^; Fig. 2h, Extended Data Fig. 2g and Supplementary Data 1). Furthermore, two cullin scaffolds, CUL-1 and CUL-6—the two closest paralogues to human CUL1—were also enriched (~5-fold change and ~20-fold change, respectively; *P* < 10^−4^; Fig. 2h), as was RBX1 (~45-fold change; *P* < 10^−5^; Fig. 2h). An analogous co-IP experiment revealed that KSS-2—like KSS-1—interacts with core components of the SCF complex (Extended Data Fig. 3f and Supplementary Data 2)

**Extended Data Fig. 4:**
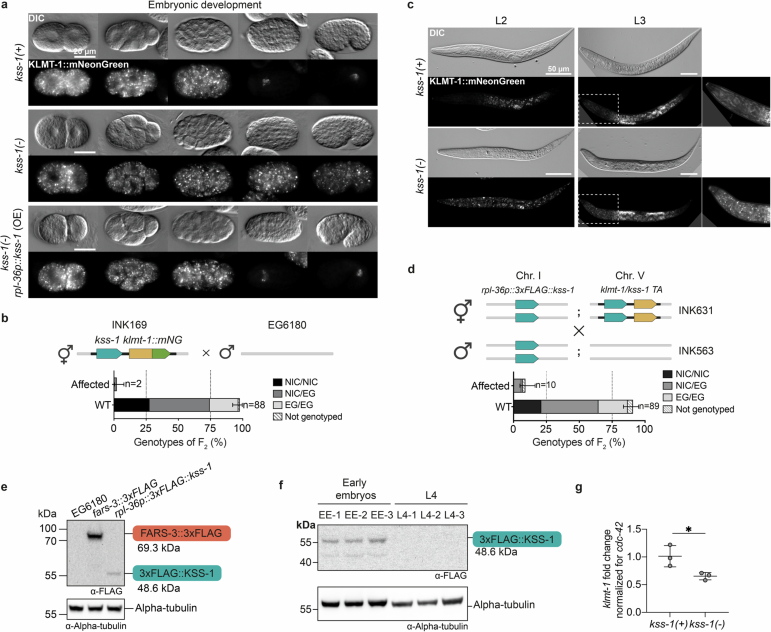
Characterization of the *klmt-1::mNeonGreen* reporter line. (**a**) KLMT-1::mNG fluorescence during embryogenesis (2-cell to comma stage). In wild-type *kss-1(+)* strain, KLMT-1::mNG levels sharply decline after early gastrulation (top). In *kss-1(-)* strain KLMT-1::mNG is present at late embryonic stages (middle). Overexpression of *kss-1* (*rpl-36p::3xFLAG::kss-1)* in the *kss-1(-) klmt-1::mNG* background leads to efficient clearance of KLMT-1::mNG and shows the specificity of this mutant phenotype. OE, overexpression. DIC, differential interference contrast. (**b**) Genetic cross between *klmt-1::mNG* strain and EG6180 revealed that the *klmt-1::mNG* allele is not toxic, as healthy homozygous EG/EG individuals are recovered in the F_2_. In (**b**) and (**d**) sample sizes are shown for each phenotypic class; error bars represent the mean with 95% CI, hybrid Wilson/Brown method. (**c**) In *kss-1(-)*, maternal KLMT-1::mNG is not effectively degraded and can be detected in the soma of L2 and L3 larval stages. Zoom-in – KLMT-1::mNG in the head of L3 larvae (bottom). Contrast is enhanced for zoom-in regions. Larvae carrying a *kss-1(+)* allele do not have KLMT-1::mNG in somatic tissues (top). (**d**) Overexpression of 3xFLAG::KSS-1 in susceptible strain EG6180 rescues the F_2_ lethality associated with the *klmt-1/kss-1* TA. (**e**) Western blot verifies 3xFLAG::KSS-1 expression from the transgenic line used in (**a**) and (**d**). Line with endogenously tagged FARS-3::3xFLAG – positive control. EG6180 – negative control. (**f**) Endogenous 3xFLAG::KSS-1 is detectable in early embryos, but not in L4s, which indicates zygotic expression of KSS-1 post-fertilization. Samples were analyzed in biological triplicates. (**g**) RT-qPCR quantification of *klmt-1* mRNA abundance from *kss-1(+)* and *kss-1(-)* lines (INK169 and INK206, respectively) normalized to *kss-1(+)* line (n = 3; two-sided unpaired *t*-test; *P* = 0.0374). Error bars represent the mean ± s.d. For this and following figures: *P ≤ 0.05, **P ≤ 0.01, *** *P* ≤ 0.001, **** *P* ≤ 0.0001; n.s. – not significant. Source data

To test whether KSS-1 targets KLMT-1 for degradation in embryos, we used a reporter strain carrying a C-terminal mNeonGreen (mNG) tag in the *klmt-1* endogenous locus (Fig. 1b and Extended Data Fig. 4a). While the addition of this fluorescent tag abrogated KLMT-1 toxicity (2.2% lethality in F_2_, *n* = 90) (Extended Data Fig. 4b), this inactive reporter allowed us to quantify KLMT-1 levels in *kss-1*(−) embryos, which would otherwise be dead. In line with this, we generated a *kss-1*(−) null allele in the *klmt-1*::*mNG* background using CRISPR–Cas, and the resulting strain was healthy and viable (94% WT, *n* = 100). Next, we quantified the expression dynamics of KLMT-1::mNG in both *kss-1*(−) and WT *kss-1*(+) embryos. In line with our hypothesis, we found that KLMT-1::mNG protein levels were largely stabilized in *kss-1*(−) embryos leading to a 2.9-fold increase in the toxin’s half-life (Fig. 2i). While KLMT-1::mNG was no longer detectable in the soma of WT embryos by the comma stage, *kss-1*(−) embryos retained somatic KLMT-1::mNG expression throughout embryogenesis; even during advanced L2/L3 larval stages (Fig. 2j and Extended Data Fig. 4c). Reintroduction of WT antidote activity via a single-copy transgene restored prompt KLMT-1::mNG degradation, fully rescuing the *kss-1*(−) mutant phenotype (Extended Data Fig. 4a,d,e). Moreover, KLMT-1 protein—but not mRNA—levels dropped sharply in early embryos coinciding with KSS-1 expression (Extended Data Fig. 4f,g). Similarly, overexpression of KSS-2 caused a marked reduction in PZL-1 fusion reporter levels (Fig. 2k), indicating that both KSS-1 and KSS-2 act as canonical F-box proteins: they bind their respective toxins and target them for degradation via the SCF E3 ligase complex.

### The *fars-3* locus harbours a cryptic TA in *C. tropicalis*

**Fig. 3:**
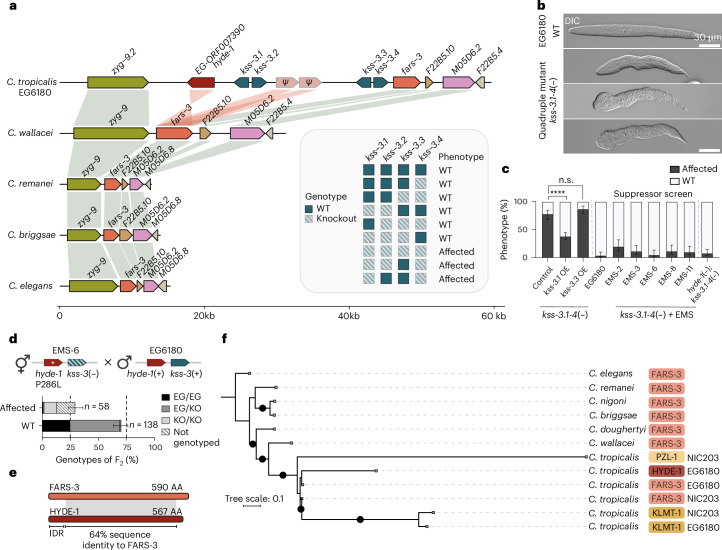
A cryptic TA in linkage to the *fars-3* parental locus. **a**, Comparison of the *fars-3* locus across *Caenorhabditis* species. Orthologous genes are connected by shading. Paralogues with homology to *fars-3* are indicated by red shading. Four paralogues of *kss-1* and *kss-2* were identified in *C. tropicalis* EG6180 between *zyg-9.2* and *fars-3*. Inset summarizes the genotype and phenotype of lines carrying various combinations of mutations in *kss-3* paralogies. **b**, Morphological defects observed in L1 larvae lacking all four *kss-3* paralogues, denoted as *kss-3.1-4*(−), strain INK950. EG6180 is a WT reference. **c**, Barplots summarizing the percentage of affected *kss-3.1-4*(−) embryos in various genetic backgrounds: (i) Overexpression (OE) of *kss-3.1* or *kss-3.3* (*P* < 0.0001 and *P* = 0.0967, respectively, two-sided Fisher’s exact test, ≥100 embryos per condition). The *kss-3.1-4*(−) strain serves as a control. (ii) Suppressor alleles recovered from an EMS mutagenesis screen (EMS-2, EMS-3, EMS-6, EMS-8 and EMS-11, ≥60 embryos per condition). (iii) *ORF007390* (*hyde-1*) null allele generated using CRISPR–Cas (100 embryos). Error bars represent the mean with 95% confidence interval (CI), hybrid Wilson/Brown method. **d**, Genetic cross between the suppressor line EMS-6 and the EG6180 wild isolate. EMS-6 carries a putative null mutation of *hyde-1*[P286L] as well as null mutations of all four *kss-3* paralogues. As expected, if *hyde-1*/*kss-3* was a TA, ~25% of their F_2_ progeny is affected and these individuals are homozygous for the susceptible haplotype. Error bars represent the mean with 95% CI, hybrid Wilson/Brown method. The KO/KO genotype corresponds to *hyde-1*[P286L] *kss-3.1-4*(−) homozygous individuals. **e**, Comparison of FARS-3 and HYDE-1 protein domains and shared homology. The per cent identity value is calculated on the basis of the local alignment. IDR, intrinsically disordered region. AA, amino acid. **f**, DNA-based phylogenetic tree showing the evolutionary relationship between *klmt-1*, *pzl-1*, *hyde-1* and *fars-3*. Black dots denote branches with a bootstrap value (SH-aLRT) >80%. Source data

**Extended Data Fig. 5:**
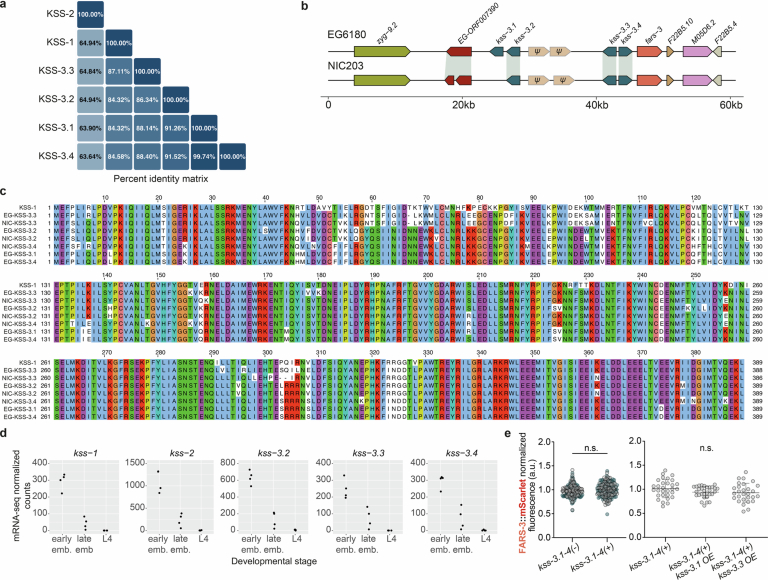
A cluster of tandem *kss* paralogs next to the *fars-3* locus. (**a**) Protein sequence similarity among various KSS paralogs. Percent identity values are calculated in Clustal Omega. (**b**) Schematic representation of *fars-3* locus in EG6180 and NIC203. (**c**) Multiple sequence alignment of KSS-1 and KSS-3 paralogs from EG6180 and NIC203. Colors correspond to the Clustal X Color Scheme. (**d**) Expression levels of NIC203 *kss-1*, *kss-2*, *kss-3.2*, *kss-3.3*, and *kss-3.4* at three different developmental stages (emb. – embryo). Expression was quantified by mRNA-seq performed in biological quadruplicates. (**e**) Quantification of FARS-3::mScarlet levels in the wild type *kss-3.1-4(+)* and *kss-3.1-4(-)* quadruple mutant background (two-sided unpaired t-test; *P* = 0.0641) (left). Experiment was performed twice; ≥ 93 embryos per condition per replicate; biological replicates shown in gray and cyan. Quantification of FARS-3::mScarlet levels in the wild type *kss-3.1-4(+)* background and in lines overexpressing *kss-3.1* or *kss-3.3* under heat-shock induction from single copy insertion transgenes (right). Brown-Forsythe ANOVA (F*(2, 73.38) = 2.094, *P* = 0.1305). Experiment performed once; ≥ 28 embryos per condition. Error bars for both represent the mean ± s.d. Source data

Intrigued by the recurrent ability of *fars-3* to give rise to genetic parasites, we examined the *C. tropicalis fars-3* locus in closer detail. In *C. elegans*, *fars-3* is part of a polycistronic operon along with its upstream neighbour *zyg-9* and downstream neighbours, *F22B5.10* and *M05D6.2* (Chr. II: 8.4 Mb)^22^. Overall, the synteny of these four genes is highly conserved among *Caenorhabditis* nematodes, including *C. tropicalis* and its sister species, *C. wallacei* (Bali, Indonesia)^23^ (Fig. 3a). However, we found a large ~34-kb insertion between *C. tropicalis zyg-9.2* and *fars-3* that is missing from *C. wallacei* and other representative *Caenorhabditis* species. Within this insertion, we identified three divergent paralogues of *fars-3*, including at least two copies that exhibited signs of pseudogenization (Fig. 3a). Remarkably, we also found several closely related paralogues with extensive homology to *kss-1* and *kss-2*, which we collectively named *kss-3* (Fig. 3a and Extended Data Fig. 5a). The number of these paralogues varied depending on the isolate. For instance, EG6180 carried a cluster of four paralogues: *kss-3.1*, *kss-3.2*, *kss-3.3* and *kss-3.4*, whereas NIC203 carried three: *kss-3.2*, *kss-3.3* and *kss-3.4* (Extended Data Fig. 5b). All these paralogues code for intact proteins (Extended Data Fig. 5c), and RNA-seq profiling revealed a similar expression pattern to that of *kss-1* and *kss-2*, suggesting that they may act during early embryonic development (Extended Data Fig. 5d).

To gain insights into the function of the *kss-3* paralogues, and anticipating redundancy given their similarity, we systematically generated knockout alleles of these genes in the EG6180 background using CRISPR–Cas. Most double- and triple-knockout mutants did not display any obvious embryonic or larval phenotypes (Fig. 3a). However, double- or triple-mutant combinations missing *kss-3.1* and *kss-3.4*, as well as the knockout of all four *kss-3* paralogues, exhibited a high proportion of dead and sick progeny. For instance, in the quadruple mutant, we observed 57.2% embryonic and larval lethality, and 20% strong delay or poor fecundity (*n* = 180) (Fig. 3a and Supplementary Table 1). The phenotype of the surviving larvae included a wide range of anterior and posterior morphological defects and protrusions along the body axis, phenotypes that were never observed in embryos poisoned by KLMT-1 or PZL-1 (Fig. 3b). Overexpression of *kss-3.1*—but not *kss-3.3*—significantly rescued the mutant phenotype of the quadruple mutant (Fig. 3c), indicating that these morphological defects were specifically caused by the targeted disruption of *kss* paralogues rather than off-target mutations induced by CRISPR–Cas.

Having confirmed the specificity of the quadruple knockout strain, we investigated why loss of *kss-3.1* and *kss-3.4* resulted in embryonic and larval defects. Given their sequence similarity to KSS-1 and KSS-2 (Extended Data Fig. 5c), we hypothesized that these genes might also function as F-box proteins, degrading an unknown target. Considering that the *kss* paralogues are located immediately upstream of *fars-3*—echoing the architecture of *klmt-1*/*kss-1* and *pzl-1*/*kss-2* loci—we first tested whether these genes could degrade FARS-3. To do so, we quantified FARS-3 protein levels upon deletion or overexpression of *kss* paralogues using an endogenously tagged *fars-3*::*mScarlet* reporter line but found no significant differences compared with controls (Extended Data Fig. 5e). Next, we adopted an unbiased genome-wide approach. The quadruple-knockout strain is very sick but viable; those few embryos that reach adulthood are usually fertile, allowing the mutant strain to be propagated indefinitely. Hence, we reasoned that an ethyl methanesulfonate (EMS)-forward genetic screen could help us identify the putative target among its suppressors^24^. Following mutagenesis and a fitness competition selection regime, we isolated five independent suppressor lines. To validate these lines, we quantified their percentage of embryonic and larval lethality, which closely resembled the WT line, EG6180 (Fig. 3c). Finally, we sequenced the whole genome of the parental and these five suppressor lines, identified EMS-derived de novo mutations and predicted their coding impact. Four out of five suppressor lines carried either non-synonymous or splicing-altering variants in the same gene: *EG-ORF007390* (Supplementary Data 3).

**Extended Data Fig. 6:**
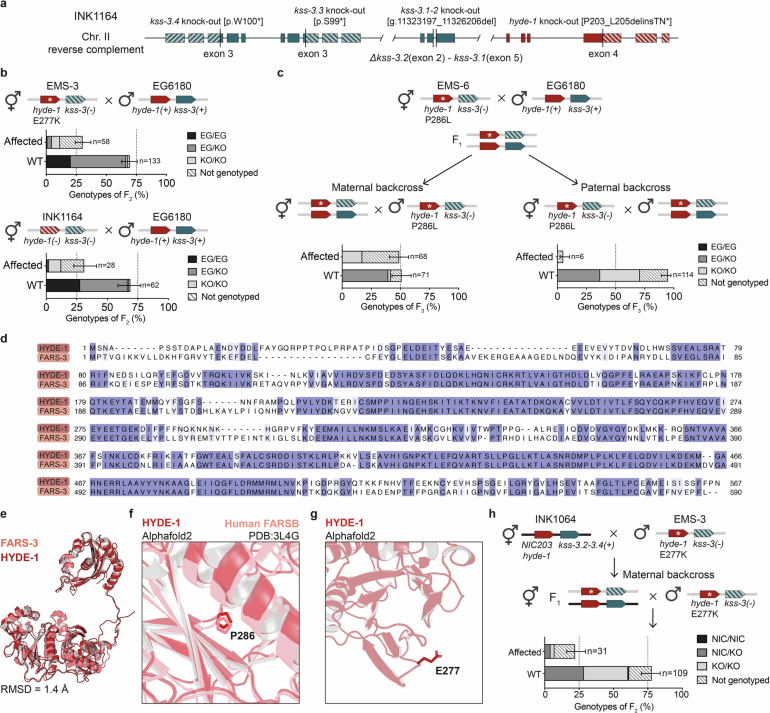
*hyde-1/kss-3* is another *fars-3*-derived maternal-effect TA. (**a**) Schematic of the INK1164 carrying CRISPR/Cas-derived null mutations in *kss-3.1-3.4*, and in *ORF007390* (*hyde-1)*. (**b**) Crosses between the suppressor line EMS-3 (INK1092) and EG6180 (top) and *hyde-1(-*) *kss-3.1-4(-)* (INK1164) and EG6180 (bottom) show ~ 25% affected F_2_ progeny, at large homozygous for the *hyde-1*(-) *kss-3.1-4(-)* allele. KO/KO corresponds to *hyde-1*[E277K] *kss-3.1-4(-)* or *hyde-1*(-) *kss-3.1-4(-)*. **(c**) Maternal- versus paternal-effect diagnostic crosses for HYDE-1. In a maternal backcross, heterozygous F_1_ hermaphrodites, derived from a cross between EMS-6 line and EG6180, are mated with EMS-6 males (left). In a paternal backcross, heterozygous F_1_ males are mated with EMS-6 hermaphrodites (right). Maternal-effect (the toxin is deposited in eggs and not in sperm) results in ~ 50% lethality in maternal backcross. Paternal backcross shows no effect, confirming maternal-effect of HYDE-1. KO/KO genotype corresponds to *hyde-1*[P286L] *kss-3.1-4(-)*. (**d**) Protein sequence alignment of HYDE-1 and *C. tropicalis* FARS-3. (**e**) Alphafold2-predicted structures of HYDE-1 and *C. tropicalis* FARS-3. (**f**) Alignment of HYDE-1 model and human FARSB highlighting Pro286, highly conserved residue mutated in the EMS-6 line (Pro286Leu). (**g**) HYDE-1 model highlighting Glu277, mutated in the EMS-3 line (Glu277Lys). (**h**) *NIC-ORF007668* codes for HYDE-1 in NIC203, shares 56% of protein sequence identity to HYDE-1 toxin in EG6180 and carries a premature stop codon. To test whether the *NIC-hyde-1* behaves as a toxin, we crossed hermaphrodites of a NIC203 strain (INK1064) in which all known toxins acting against EG6180 (*pzl-1*, s*low-1*, *klmt-1*, and *NIC-ORF019770*) had been mutated using CRISPR/Cas with males of the suppressor EMS-3 line that carries an inactive *hyde-1/kss-3* TA. Crosses between heterozygous F_1_ hermaphrodites and EMS-3 males confirm NIC203 *hyde-1* was inactive: homozygous *hyde-1* mutants corresponding to EMS-3 allele were observed in a 1:1 ratio as heterozygous. KO/KO genotype corresponds to *hyde-1*[E277K] *kss-3.1-4(-)*. For (**b**), (**c**), (**h**) sample sizes are shown for each phenotypic class, error bars represent the mean with 95% CI, hybrid Wilson/Brown method. In (**b**), (**c**) and (**h**) the asterisk marks a mutation in *hyde-1*. Source data

*ORF007390* is one of the *fars-3* paralogues located downstream of *zyg-9.2* in *C. tropicalis* (Fig. 3a). To confirm that mutations in *ORF007390* suppressed the mutant phenotype, we generated *ORF007390*(−) null mutants using CRISPR–Cas (Extended Data Fig. 6a). The quadruple *kss-3* knockout strain was fully viable and healthy in an *ORF007390*(−) genetic background, thus validating the candidate suppressor (Fig. 3c). The discovery that *ORF007390* and the *kss-3* paralogues were together dispensable for embryonic development led to an unexpected revelation: ORF007390 was not a pseudogene but a toxin, and *kss-3.1* and *kss-3.4* were its cognate antidotes. In agreement with the TA model, we crossed EG6180 with the EMS-6 suppressor line and observed developmental defects in ~25% of the F_2_ progeny and affected embryos were homozygous for the EMS-derived suppressor allele (Fig. 3d). The same pattern of F_2_ lethality was observed when crossing the suppressor EMS-3 with EG6180 and the CRISPR-derived *ORF007390*(−) *kss-3.1-4*(−) mutant line with EG6180 (Extended Data Fig. 6b). Furthermore, reciprocal maternal and paternal backcrosses indicated that ORF007390 was a maternal-effect toxin, like *klmt-1* and *pzl-1* (Extended Data Fig. 6c). Based on these findings, we named the new toxin *hyde-1* (for harmful phenylalanine–tRNA synthetase duplicate). Unlike all other TAs, *hyde-1*/*kss-3.1,4* is tightly linked to its parental locus (Fig. 3a). As the *hyde-1*/*kss-3.1,4* TA spreads in the population via gene drive, the linked *fars-3* haplotype is predicted to rise in frequency through genetic hitchhiking—without any direct benefit to the TA^25^. This represents a unique case in which a selfish genetic element could indirectly promote the persistence of its non-selfish parental gene.

HYDE-1 is 567 amino acids long and, unlike KLMT-1 and PZL-1, shares substantial homology with both the N-terminal and C-terminal domains of FARS-3 (Fig. 3e and Extended Data Fig. 6d,e). Among the suppressors of *kss-3* loss of function, we identified a Pro286Leu substitution (line EMS-6). This proline is highly conserved across FARS-3 orthologues and probably hinders the correct folding of the toxin (Extended Data Fig. 6f). Also, we found a Glu277Lys substitution (line EMS-3), which changes a negative charge for a positive one in a solvent exposed residue (Extended Data Fig. 6g). The parental NIC203 carries a divergent copy of *hyde-1*, which is truncated and not toxic (Extended Data Figs. 5b and 6h). The *hyde-1*/*kss-3-1,4* TA is cryptic, as it does not cause a genetic incompatibility in crosses between NIC203 and EG6180, probably because NIC203 *kss-3.4* expression is sufficient to neutralize HYDE-1 (Extended Data Fig. 5c,d). Thus, we identified a third maternal-effect TA derived from the *fars-3* locus. Although related, the toxins from all three TAs appear to have different mechanisms, and the KSS antidotes are highly specific for their toxins.

### Multiple independent duplication events of the *fars-3* locus

To better understand the evolutionary relationships between *klmt-1*, *pzl-1*, *hyde-1* and *fars-3*, we constructed a DNA-based phylogenetic tree (Fig. 3f and Supplementary Fig. 4a,b). To ensure accuracy and reliability, we leveraged sequence conservation and structural models to exclude predicted intrinsically disordered regions from the toxins. Further, for *pzl-1*, we included only the region homologous to *fars-3* (Methods). The phylogeny revealed that all three toxins originated from *fars-3* following the divergence of *C. tropicalis* and its sister species, *C. wallacei*. Remarkably, the toxins did not form a monophyletic group, instead, we found strong statistical support for at least two independent duplication events of *fars-3*. The first duplication event gave rise to *pzl-1*. Subsequent duplications led to the emergence of either *klmt-1* and *hyde-1*, or their common ancestor; however, distinguishing between these models was not possible. The oldest age of the *pzl-1*/*kss-2* TA is also consistent with the fact that PZL-1 is the most derived toxin, including homology to MEC-15, which was probably a secondary acquisition (Fig. 1f and Extended Data Fig. 1i). What is the molecular basis for the recurrent evolution of these genetic parasites from *fars-3*? To address this question, we examined the evolutionary origins of KSS antidotes in *Caenorhabditis* species.

### KSS antidotes are evolving rapidly, targeting new substrates

**Extended Data Fig. 7:**
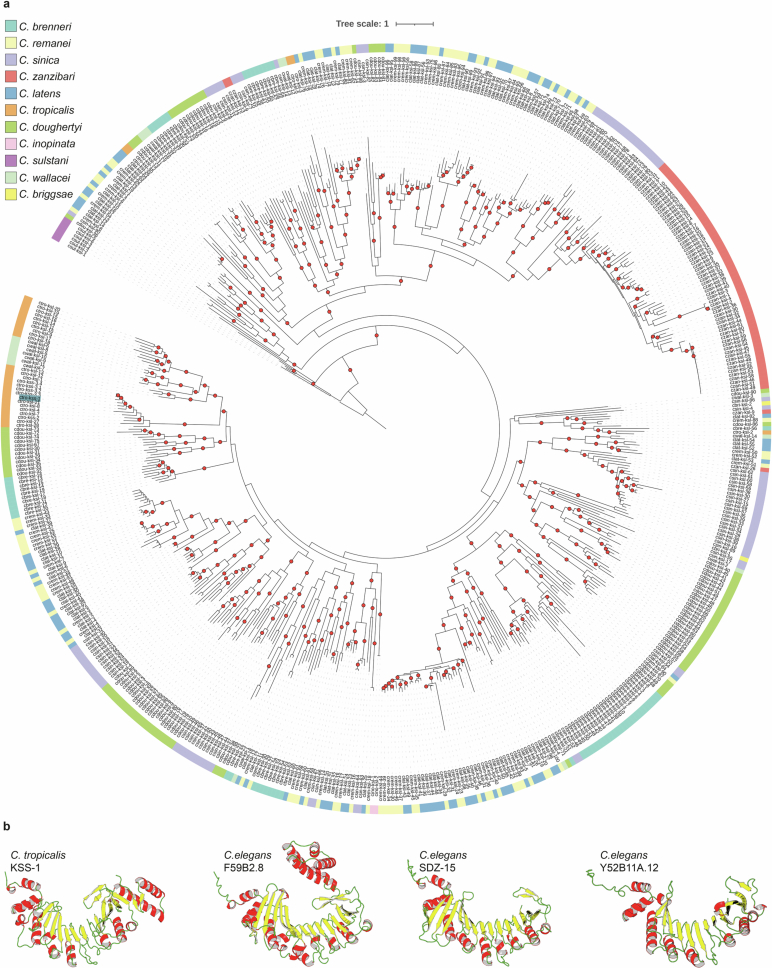
Phylogenetic tree of KSL proteins across nematodes. (**a**) Phylogenetic tree of 560 KSL proteins from *Caenorhabditis* species. Red dots denote branches with SH-aLRT ≥ 80% and UFboot ≥ 95%. The nucleotide alignment was filtered to retain only nucleotide positions covered by ≥ 80% of the sequences. Colors indicate different species (inset). (**b**) Examples of *C. elegans* proteins structurally similar to *C. tropicalis* KSS-1 identified by Foldseek. F29B2.8 (prob = 0.8, Tm = 0.59), SDZ-15 (prob = 0.84, Tm = 0.67), and Y52B11A.12 (prob = 0.82, Tm = 0.68). Alpha-helices are shown in red, beta strands in yellow. Full list available in Supplementary Data 5.

An exploratory screen for homologous sequences across 55 *Caenorhabditis* species revealed that these were primarily restricted to the *Elegans* group—with only a few strong matches in *C. sulstoni*, which belongs to the *Japonica* group. Thus, we selected 11 *Elegans*-group species with chromosome-level assemblies, as well as *C. sulstoni*, annotated genes homologous to KSS-1 using a curated hidden Markov model (HMM) and constructed a comprehensive phylogenetic tree (Extended Data Fig. 7a). In total, we identified 560 homologues across 12 species, which we named *ksl* (for KSS-like genes) (Supplementary Data 4). The phylogeny of KSL proteins revealed extensive expansion and diversification of this family. Most KSL proteins were species-specific, a pattern similar to that observed in canonical F-box proteins from *C. elegans* and other nematodes, which are known to form large clusters on chromosomal arms—regions typically associated with elevated recombination rates^26,27^ (Extended Data Fig. 7a). The species with the highest number of KSL proteins were *C. remanei* (100), *C. latens* (98) and *C. doughertyi* (90). By contrast, *C. briggsae* and *C. inopinata* contained only 2 KSL proteins each, and no homologues were identified in *C. elegans*. The low number or absence of KSL homologues in these and other species could, in part, be explained by the rapid diversification of F-box proteins, which makes sequence-based identification challenging. Supporting this view, a structural-based search found 221 *C. elegans* proteins with significant similarity to KSS-1 (Extended Data Fig. 7b and Supplementary Data 5; Foldseek Prob. >0.5 and Tm-score >0.5).

**Fig. 4:**
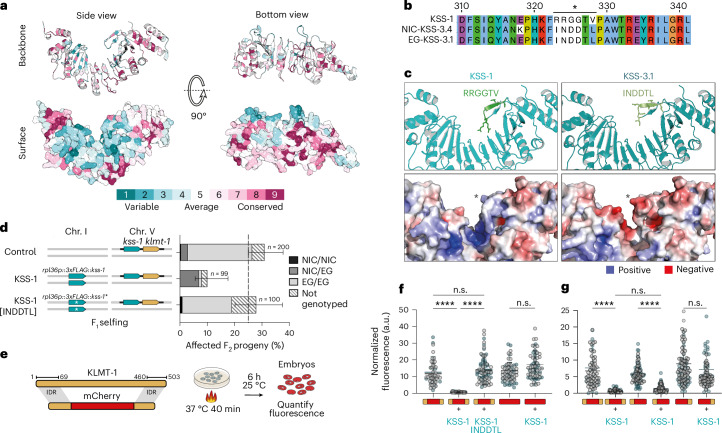
The fast-evolving inner solenoid surface of KSS antidotes mediates substrate recognition. **a**, Conservation of the KSS antidotes and KSL paralogues (ConSurf). **b**, Alignment of KSS-1 and KSS-3 from EG6180 and NIC203. The asterisk marks one of the most variable segments between KSS-1 and KSS-3 that is located on a beta strand opposing the inner solenoid surface. **c**, Top: KSS-1 and KSS-3.1 with variable six-amino-acid loop marked in shades of green (AlphaFold2). Bottom: electrostatic potential of KSS-1 and KSS-3.1. The asterisk marks the variable loop. **d**, Overexpression of 3xFLAG::KSS-1 rescues F_2_ lethality associated with the *klmt-1*/*kss-1* TA in EG6180, but 3xFLAG::KSS-1 (RRGGTV > INDDTL) does not. Error bars represent the mean with 95% CI, hybrid Wilson/Brown method. **e**, Fluorescent reporter assay to test the sufficiency of KLMT-1 IDRs for KSS-1-mediated degradation. mCherry reporters were expressed as single-copy heat-shock inducible transgenes on Chr. IV. Extracted embryos were heat-shocked for 40 min and imaged after 6 h recovery. **f**, Fusion of the N- and C-terminal IDRs of KLMT-1 to mCherry was sufficient for KSS-1-dependent degradation (*P*_adj_ < 0.0001). mCherry alone was not degraded (*P*_adj_ > 0.9999). The KSS-1 mutant failed to degrade IDR-mCherry-IDR (KSS-1 mutant versus no KSS-1: *P*_adj_ > 0.9999, KSS-1 mutant versus KSS-1: *P*_adj_ < 0.0001). KSS-1 is expressed under a constitutive promoter (*Ctr*-*rpl-36p*), Chr. I. Kruskal–Wallis test followed by Dunn’s post-hoc test (*H*(5) = 133.1, *P* < 0.0001). **g**, N-terminal IDR is sufficient for KSS-1-mediated degradation (IDR-mCherry-IDR only versus with KSS-1: *P*_adj_ < 0.0001, IDR-mCherry only versus with KSS-1: *P*_adj_ < 0.0001), but not C-terminal IDR (mCherry-IDR with KSS-1: *P*_adj_ = 0.4314). KSS-1 degrades IDR-mCherry with similar efficiency as does IDR-mCherry-IDR (*P*_adj_ = 0.5937). Kruskal–Wallis test followed by Dunn’s post hoc test (*H*(6) = 306.8, *P* < 0.0001). For **f** and **g**: error bars represent the mean ± s.d. Experiments repeated twice; ≥14 embryos per condition per replicate; biological replicates shown in grey and cyan. Source data

**Extended Data Fig. 8:**
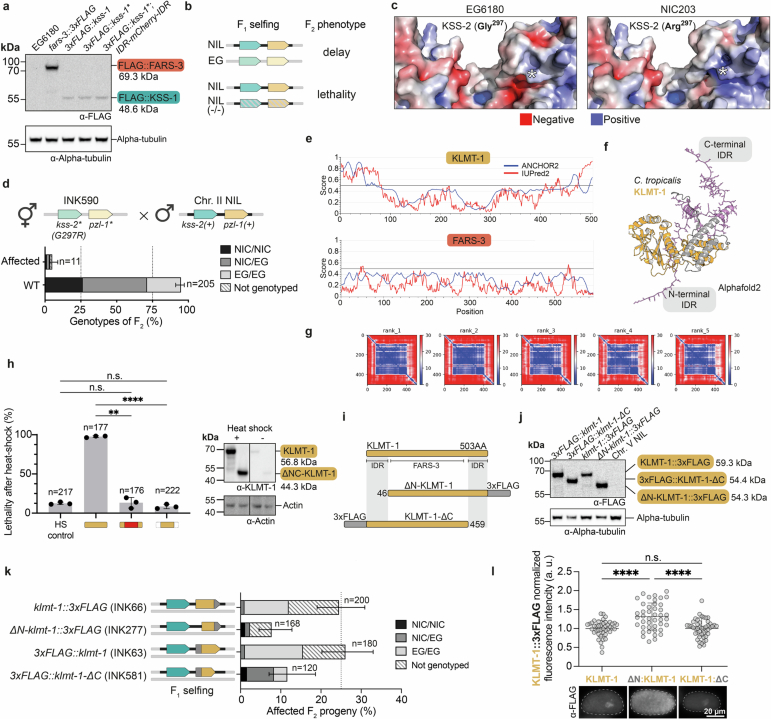
KSS-2 polymorphism affects PZL-1 clearance; KLMT-1 N-terminal IDR is necessary and sufficient for KSS-1 mediated degradation. (**a**) Western blot shows comparable expression of 3xFLAG::KSS-1 and 3xFLAG::KSS-1[INDDTL] (INK563 versus INK777 or INK806). FARS-3::3xFLAG – positive control, EG6180 – negative control. (**b**) Crossing Chr. II NIL to EG6180 shows developmental delay, but crosses with *pzl-1(-) kss-2(-)* or PZL-1 overexpression cause embryonic/larval lethality (Fig. 1g and Extended Data Fig. 1f). (**c**) EG6180 carries KSS-2 with Leu74Met and Arg297Gly substitutions, likely a hypomorph. Electrostatic potential of KSS-2 models, Alphafold2. Solvent-exposed Arg297 (asterisk) lies on the inner solenoid surface. (**d**) Gly297Arg reversion in the *EG-kss-2* restores antidote function and explains the difference in toxicity phenotype. The Arg297Gly variant is present in 223/690 wild isolates (CaeNDR). Sample sizes are shown for each phenotypic class. For (**d**) and (**k**) error bars represent the mean with 95% CI, hybrid Wilson/Brown method. (**e**) IDRs prediction. (**f**) AlphaFold2 KLMT-1 model. Orange – high-confidence region. Violet – IDRs. Gray – low confidence (AlphaFold2), but not disordered (IUPred2A) regions. (**g**) Predicted aligned error of KLMT-1 models. (**h**) Overexpression of mCherry with KLMT-1 IDRs or truncated KLMT-1 does not increase lethality upon heat-shock (IDR-mCherry-IDR versus control: *P*_*adj*_ = 0.3171; IDR-mCherry-IDR versus KLMT-1: P_*adj*_ = 0.0054; truncated KLMT-1 versus control *P*_*adj*_ = 0.9989; truncated KLMT-1 versus KLMT-1: *P*_*adj*_ < 0.0001). Brown-Forsythe ANOVA (F*(3, 3.058) = 386.3, *P* = 0.0002), followed by Dunnett post hoc test. Dots indicate individual replicates; ≥ 50 embryos per replicate. Error bars represent the mean with 95% CI, hybrid Wilson/Brown method. Western blot confirms expression (right). No induction – negative control. (**i**) Endogenous KLMT-1 truncations to test IDRs necessity. N-terminal truncation was chosen based on ANCHOR2 prediction and is 24 amino acids shorter than N-terminal IDR fused to mCherry (Fig. 4e–g). (**j**) Western blot confirms expression of truncated alleles. Chr. V NIL – negative control. (**k**) Crosses to EG6180 indicated that both IDRs are necessary for toxicity. (**l**) Immunofluorescence shows that N-terminal, but not C-terminal IDR is necessary for degradation *in vivo* (ΔN-KLMT-1 versus KLMT-1: *P*_*adj*_ < 0.0001; ΔN-KLMT-1 versus ΔC-KLMT-1: *P*_*adj*_ < 0.0001; ΔC-KLMT-1 versus KLMT-1: *P*_*adj*_ > 0.9999). KLMT-1 levels were quantified at the late gastrulation and bean stages. Brown-Forsythe ANOVA (F*(2, 102.8) = 18.52, *P* < 0.0001), followed by Games-Howell post hoc test. Error bars represent the mean ± s.d. Representative embryos of late gastrulation stage (bottom). Source data

Including known antidotes, we identified 30 KSL proteins in *C. tropicalis* (Extended Data Fig. 7a). The most divergent residues across *C. tropicalis* KSL proteins were found in the inner surface of the solenoid (Fig. 4a). Thus, we hypothesized that this fast-evolving region might contribute to substrate recognition, similar to the role of the LRR domain in F-box proteins^28,29^. To test this idea, we examined in closer detail KSS-1 and KSS-3.1, which are highly specific antidotes despite being 84.3% identical at the protein level (Extended Data Fig. 5c). Upon inspecting both sequences, we identified a highly variable region between KSS-1 and KSS-3.1 within a protruding β-hairpin motif facing the inner solenoid, which conferred opposite charge distributions (Fig. 4b,c). To determine if this region contributes to substrate specificity, we replaced a 6-amino-acid patch in the KSS-1 β-hairpin (RRGGTV) with the corresponding sequence in KSS-3.1 (INDDTL). In controlled transgenic experiments, overexpression of a WT copy of KSS-1 fully rescued the lethality associated to KLMT-1, whereas the KSS-1[INDDTL] chimera did not, indicating that these residues are required for the specificity of KSS-1 towards KLMT-1 (Fig. 4d). As a control, we confirmed that differences in protein expression levels of these transgenes did not account for variations in antidote activity (Extended Data Fig. 8a). Similarly to KSS-1, we identified a natural variant in KSS-2 at a rapidly evolving solvent-exposed residue (Arg297), which reduced the efficiency of PZL-1 degradation (Extended Data Fig. 8b–d), further supporting the idea that fast-evolving residues play a key role in toxin recognition.

Next, we investigated which region of KLMT-1 is recognized by KSS-1. We hypothesized that the disordered domains of KLMT-1 are crucial for this interaction, as they are absent in its paralogue FARS-3 (Fig. 1c and Extended Data Fig. 8e–g). To test this, we designed a chimeric construct by fusing both N-terminal and C-terminal disordered domains of KLMT-1 to mCherry (AAs: 1–69 and AAs: 460–503, respectively), which did not exhibit any toxic activity upon overexpression (Fig. 4e and Extended Data Fig. 8h). We then quantified the abundance of the chimeric protein in embryos in the presence and absence of KSS-1. Our results showed that the disordered domains of KLMT-1 are sufficient to target mCherry for degradation by KSS-1 and that KSS-1[INDDTL] could not degrade the reporter (Fig. 4f). As a negative control, KSS-1 had no effect on the expression levels of mCherry alone (Fig. 4f). Lastly, we determined that the N-terminal disordered region of KLMT-1 was both sufficient and necessary for KSS-1-mediated degradation (Fig. 4g and Extended Data Fig. 8i–l). Together, these results strongly suggest that KSL proteins quickly evolve specificity for new degron sequences, leading to the recognition and degradation of new substrates.

### Duplication of the *kss*/*fars-3* module led to recurrent TA evolution

**Fig. 5:**
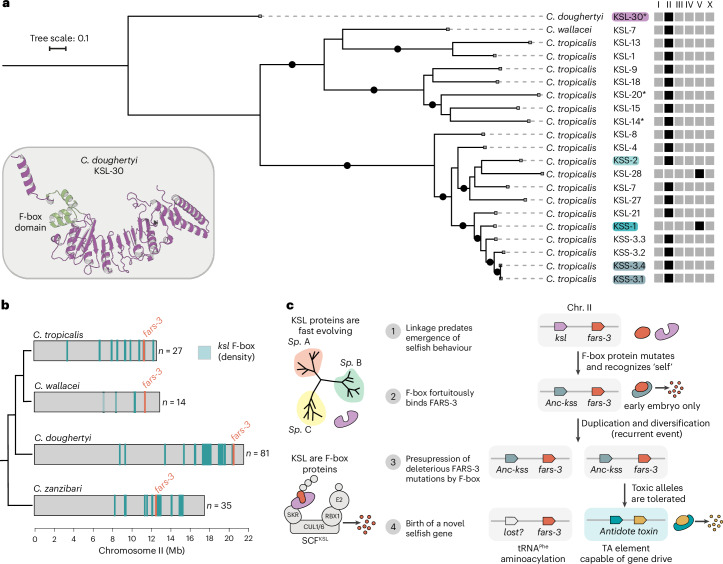
The emergence of KSS antidotes and a model for the recurrent evolution of TAs from *fars-3.* **a**, Protein-based phylogenetic tree of the KSS antidotes, their closest paralogues in *C. tropicalis* and representative homologues of *C. wallacei* and *C. doughertyi*. *C.dou-*KSL-30 was designated as the outgroup. Black dots denote branches with a bootstrap value (SH-aLRT) >80%. The chromosome where each KSS paralogue is located is indicated in the diagram on the right. KSL proteins with F-box domain predicted by InterProScan are marked with an asterisk. **b**, The relative position of *fars-3* and *ksl* F-box genes on Chr. II across representative species of the *Elegans* group. Position of ksl genes is semi-transparent to emphasize the positions of clusters; *n* denotes the number of *ksl* genes in Chr. II. **c**, KSL proteins are fast-evolving and largely species-specific proteins that are capable of recruiting targets to the SCF complex. (1) A KSL F-box protein, part of a rapidly evolving cluster on Chr. II, is found in linkage with *fars-3*. (2) This F-box fortuitously acquired affinity for FARS-3—analogous to an autoimmune interaction—giving rise to the ancestral KSS antidote (Anc-KSS). Anc-KSS was probably expressed exclusively in the early zygote. (3) After the duplication of the *fars-3*/Anc-*kss* genetic module and under relaxed selection, the divergent FARS-3 paralogue became toxic, with the linked F-box protein providing presuppression of toxic alleles by targeted degradation and concurrently becoming its antidote. (4) Over time, positive selection for gene drive efficiency enhanced both toxicity and antidote specificity, which led to the birth of the TA element.

**Extended Data Fig. 9:**
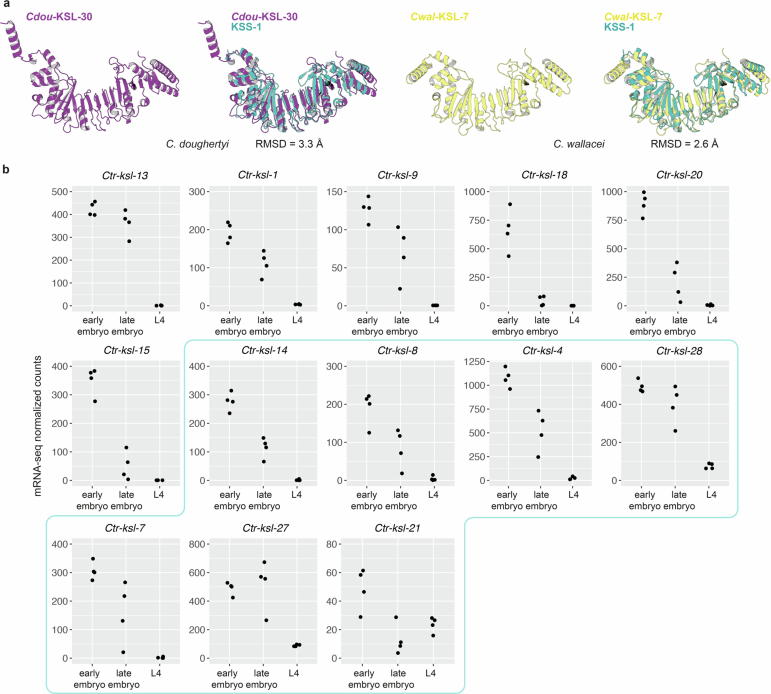
KSL paralogs have a similar structure and expression pattern to KSS antidotes. (**a**) Alphafold2 models of *Cdou-*KSL-30 and *Cwal-*KSL-7 (left). Predicted structures of both paralogs are highly similar to KSS-1 (right). (**b**) Expression levels of *ksl* paralogues identified on Chr. II of *C. tropicalis* (Fig. 5a). Paralogs that form a clade with characterized antidotes are marked in cyan. Expression was quantified by mRNA-seq in biological quadruplicates.

We identified sister clades to the KSS antidotes in *C. wallacei* and *C. doughertyi*, the two closest relatives to *C. tropicalis* (Extended Data Fig. 7a). Although representative KSL proteins, such as *Cwal-ksl-7* and *Cdou-ksl-30*, were only 35% and 28% identical to KSS-1, Alphafold2 models of these proteins suggested extensive structural similarity to KSS-1, confirming their shared ancestry (Fig. 5a and Extended Data Figs. 7a and 9a). In addition, InterProScan detected a canonical F-box domain sequence on the N terminus of some KSL proteins (Fig. 5a). While we cannot discard this is the result of convergent evolution, a more plausible explanation is that KSS antidotes share homology with canonical F-box proteins, with their N-terminal sequences having diverged beyond easy recognition. Surprisingly, we found that *ksl* genes were not randomly distributed across all six equally sized chromosomes: 78.4% (439/560) of *ksl* genes were located on Chr. II (Fig. 5a). This bias was present in all ten species from the *Elegans* group where *ksl* were identified, including *C. tropicalis* (90%), *C. wallacei* (82.4%) and *C. doughertyi* (90%). Furthermore, *ksl* genes were not evenly distributed within Chr. II, but formed large clusters (Fig. 5b). These clusters were found in close proximity to their respective *fars-3* orthologues—approximately 1 Mb or less in the two closest relatives of *C. tropicalis* (Fig. 5b and Supplementary Data 5). Altogether, these results strongly suggest that the *ksl* family originated in Chr. II and that genetic linkage between *fars-3* and the ancestor of all three antidotes predated the evolution of their selfish behaviour.

**Extended Data Fig. 10:**
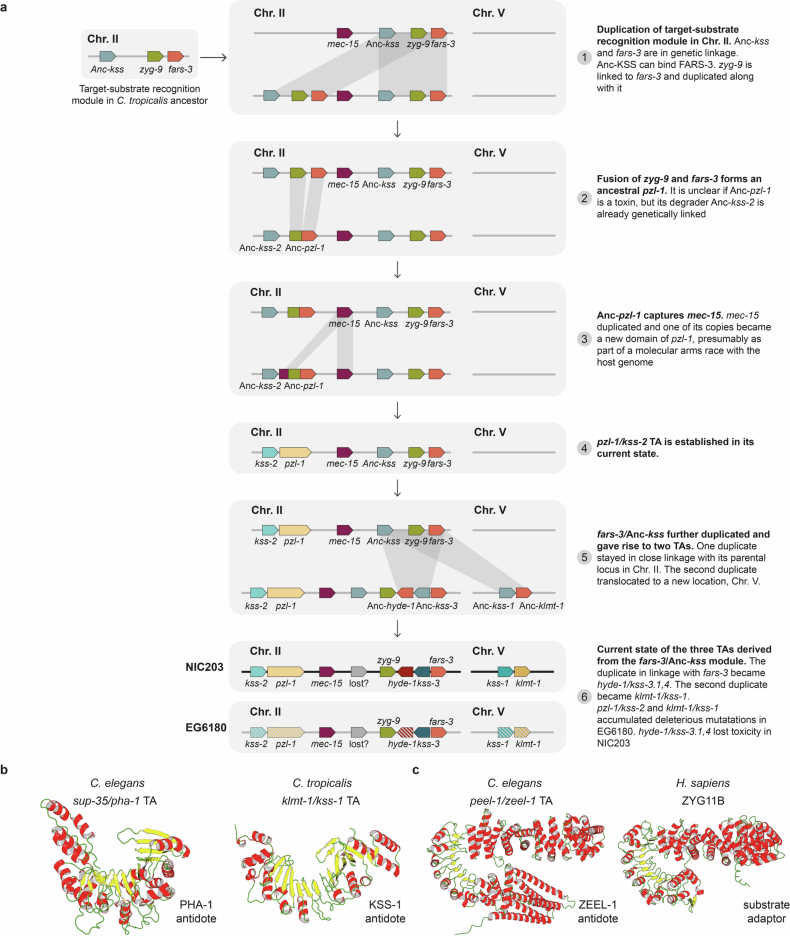
Evolutionary scenarios leading to the emergence of three TA elements from *fars-3.* (**a**) Following the split of *C. wallacei* and *C. tropicalis*, a *C. tropicalis ksl* F-box protein in linkage with *fars-3* randomly evolved affinity for FARS-3 and became the ancestor of all *kss* antidotes (Anc*-kss*). (1) The *fars-3/*Anc*-kss* module duplicated with its upstream neighbor *zyg-9*. (2) The reduced selective pressure experienced by the duplicated module facilitated the evolution of a TA element. We speculate that *Anc-pzl-1* (a chimeric protein of *zyg-9* and *fars-3*) was already mildly toxic at this stage, and the Anc-*kss* paralog became Anc-*kss-2*. (3) After the formation of the first TA system, Anc-*pzl-1* fused with a paralog of *mec-15*, potentially resulting in an increase in *pzl*-1 toxicity. (4) Current *pzl-1/kss-2* TA. (5) Either a second duplication of the *fars-3/*Anc*-kss* module gave rise to a common ancestor of the *klmt-1/kss-1* and *hyde-1/kss-3* TAs, or these TA systems evolved independently from two separate duplication events of the parental module. (6) *klmt-1/kss-1* translocated to Chr. V, whereas *hyde-1/kss-3* remained linked to its parental locus in Chr. II, providing an evolutionary advantage to *fars-3* through genetic hitchhiking. *Anc-kss* likely became pseudogenized and was lost over time, as it did not confer a selective advantage to its host. For simplicity, only one antidote of *hyde-1* is shown. (**b**) Alphafold2 structures of PHA-1 and KSS-1. PHA-1, the antidote of the *C. elegans sup-35/pha-1* TA, is predicted to have a horseshoe fold with parallel beta sheets running on its inner face and alpha helices on its exterior, analogous to KSS-1, suggesting that it may bind and degrade SUP-35. (**c**) Predicted structures of ZEEL-1 and human ZYG11B. ZEEL-1 (antidote of the *C. elegans peel-1/zeel-1* TA) is homologous to ZYG11B, which serves as a substrate adapter subunit in the E3 ubiquitin ligase complex ZYG11B–CUL2–Elongin-BC. This similarity strongly suggests a link with protein degradation. In (**b**) and (**c**) alpha-helices are shown in red and beta strands in yellow.

The phylogeny of the KSS antidotes mirrors that of the toxins and also supports the view that *pzl-1*/*kss-2* was the first TA to emerge from the *fars-3* locus (Fig. 5a and Supplementary Note). Given that the ancestral *fars-3* gene duplicated at least twice, and considering that all three antidotes share a common evolutionary origin, there are two possible scenarios that could account for our observations: either ancestral KSS F-box proteins independently evolved the ability to bind different toxins on at least two occasions, or the binding evolved only once and involved the common ancestor of all these toxins, FARS-3 (Extended Data Fig. 10a). As the second scenario is the most parsimonious and given that linkage between the ancestral *kss* F-box proteins and *fars-3* predated the emergence of the TAs, our results strongly suggest that it was the duplication of the module consisting of both the ancestral F-box protein (Anc-*kss*) and *fars-3*, that drove the recurrent evolution of these selfish elements (Fig. 5c). Furthermore, our data support the conclusion that this fortuitous interaction neutralized the toxicity of *fars-3* paralogues before it arose (presuppression), allowing otherwise harmful mutant alleles to persist and ultimately evolve into selfish genes (Fig. 5c).

Given the essential role of FARS-3, how could Anc-KSS evolve an affinity for FARS-3 if doing so would result in its degradation? To explore this, we examined the expression patterns of *C. tropicalis* KSL paralogues. We found that 10 out of 13 *C. tropicalis* KSL paralogues (including also representatives of the more distant sister clade) are transcribed only during a brief window in early development, with no detectable expression at the L4 stage, like the KSS antidotes (Fig. 5a and Extended Data Fig. 9b). This pattern strongly suggests that transient zygotic expression is the ancestral state of this protein family and did not evolve as a consequence of their role as an antidote. Moreover, this explains how the degradation of FARS-3 by Anc-KSS could have been tolerated. As FARS-3 is constitutively expressed throughout all embryonic and larval stages, the ancestral F-box would have had only a short-lived effect on its dosage.

## Discussion

From CRISPR–Cas complexes to the establishment of memory in the nervous system, selfish genes are at the core of many key prokaryotic and eukaryotic innovations. However, the molecular mechanisms by which selfishness itself arises from genomes are largely unknown. In this study, we uncovered one such mechanism: the gratuitous degradation of newly acquired substrates by F-box proteins, which permits toxic alleles to escape purifying selection and facilitates the emergence of selfish traits. Although we cannot formally discard the possibility that *fars-3* paralogues might have initially conferred a fitness advantage to their host, our findings are more consistent with the theory of constructive neutral evolution^30–32^.

Constructive neutral evolution posits that complexity can increase through a series of neutral steps^30,33–36^. Critical for this theory is the concept of presuppression, wherein a pre-existing, non-essential interaction buffers the negative effects of deleterious mutations. In our model system, this ‘excess capacity’ stems from the massive expansion and diversification of F-box proteins, which generate a broad repertoire of protein binders capable of fortuitously suppressing harmful alleles. Although the transient evolutionary nature of the interaction between ancestral FARS-3 and KSS antidotes may have precluded its direct observation in extant nematodes, several lines of evidence indicate that such interactions are both plausible and recurrent.

First, a recent study on a hybrid incompatibility between two closely related nematode species identified two underlying loci: *shls-1*, an essential phosphoglucomutase in *C. briggsae*, and *neib-1*, a rapidly evolving, dispensable F-box protein in *C. nigoni*^37^. In F_1_ hybrids, the zygotically expressed F-box protein specifically mediates the degradation of the phosphoglucomutase during embryogenesis, demonstrating that F-box proteins can—over evolutionary time—fortuitously evolve binding to essential proteins and target them for degradation. Second, two TA systems in *C. elegans*—*sup-35*/*pha-1* and *peel-1*/*zeel-1*—probably evolved via a similar presuppression mechanism^4,38^. The SUP-35 toxin evolved via gene duplication from a RMD-2, a conserved microtubule-binding protein^4^ and AlphaFold2-based structural predictions suggested that the antidote PHA-1 is analogous to KSS-1 (Extended Data Fig. 10b). Furthermore, the antidote ZEEL-1 shares homology with a conserved substrate-recognition subunit of the Cullin-2–RING E3 ubiquitin ligase complex^39,40^ (Extended Data Fig. 10c), suggesting that presuppression mechanisms could also be a feature of other protein degradation complexes.

The involvement of substrate-recognition proteins in the evolution of TAs also extends beyond nematodes. In plants, for example, HTA, the antidote of a TA causing pollen sterility in rice hybrids, also encodes an F-box protein^41^. Our model may further explain the recurrent evolution of self-incompatibility loci in plants, where cytotoxic enzymes are closely linked to detoxifying F-box proteins to enforce genetic diversity during fertilization^42^. The independent expansion and rapid evolution of F-box proteins in both nematodes and plants^27,43^ strongly suggest that these proteins may function as innate immune elements that target foreign proteins^27,44–47^. In light of this, we propose that the emergence of selfish TA systems in worms and plants—and potentially in other taxa—is an inevitable by-product of the evolutionary arms race between genome defence mechanisms and parasites.

The dual role of F-box proteins—as both protectors of the genome and facilitators of the evolution of selfish genes—finds parallels in prokaryotes, where common components of these systems, such as nucleases, are ‘guns for hire’ that can function in both genome defence and parasitism^48^. In other words, the very mechanisms that evolved to protect the host genome can inadvertently give rise to their own antagonists, thereby sowing the seeds of their own transformation. Lastly, we propose that presuppression mechanisms, as outlined by constructive neutral evolution, may be a widespread and unifying mechanism in the evolution of biological complexity, ranging from the emergence of selfishness to the evolution of sophisticated molecular machines.

## Methods

### Maintenance of worm strains

Nematodes were grown on modified nematode growth medium (NGM) plates with 1% agar/0.7% agarose to prevent *C. tropicalis* burrowing. Plates were seeded with OP50. Experiments were conducted at 25 °C. In cases of bacterial contamination, worms were bleached by adding 500 µl of a 1:1 mixture of 1 M NaOH and 5% bleach to 750 µl of worms in M9. Bleaching was stopped by washing the embryos twice with 14 ml of M9. Supplementary Tables 2 and 3 list all the strains used in this study, some of which were provided by the *Caenorhabditis* Genetics Center (CGC), funded by the National Institutes of Health Office of Research Infrastructure Programs (P40 OD010440).

### Phenotyping and genotyping of crosses

For crosses, 4–5 L4 hermaphrodites were mated with 40–50 males (1:10 ratio) in a single well of a 12-well plate with modified NGM. After 2 days, 15 L4 F_1_ progeny were transferred in bulk to a fresh plate, and then the next morning singled into individual plates, to ensure synchronized laying of F_2_ generation embryos. After 3 h of active laying, F_1_ hermaphrodites were collected for genotyping by PCR, and at least ten embryos from each heterozygous hermaphrodite were transferred to individual plates the same day. F_2_ were staged and phenotyped daily until they either died or reached sexual maturity. Embryonic lethality, larval lethality, arrested development, delayed reproduction and sterility were assessed. For maternal and paternal backcrosses, F_1_ offspring were additionally mated overnight with males or hermaphrodites of the appropriate parental strain. For genotyping by PCR, worms were digested with lysis buffer (10 mM Tris–Cl pH8, 50 mM KCl, 2.5 mM MgCl_2_ and 200 µg ml^−1^ Proteinase K). PCRs were performed using in-house hot-start Taq polymerase (Molecular Biology Service, IMBA). If necessary, PCR products were additionally analysed by Sanger sequencing. Efficiency of PCR was low for progeny arrested as embryos or at early larval stages. Detailed information on genotyping and phenotyping of crosses can be found in Supplementary Data 6.

### Generation of *C. tropicalis* transgenic lines

For CRISPR–Cas gene editing, we adapted previously described protocols^49^. In brief, 250 ng µl^−1^ Cas9 or Cas12a proteins (IDT) were incubated with 200 ng µl^−1^ crRNA (IDT) and 333 ng µl^−1^ tracrRNA (IDT) at 37 °C for 10 min before adding 2.5 ng µl^−1^ co-injection marker plasmid (pCFJ90-mScarlet-I, or pCFJ90-mNG for editing of lines expressing mScarlet-tagged proteins). For homology-directed repair, donor oligos (IDT) or biotinylated and melted PCR products were added at a final concentration of 200 ng µl^−1^ or 100 ng µl^−1^, respectively. Following injections into young hermaphrodites, mScarlet or mNG-positive F_1_ progeny were singled and their offspring screened by PCR and Sanger sequencing to detect successful editing. To insert single-copy transgenes we adapted a split selection system developed for *C. elegans*^50^. We introduced two synthetic landing pads (SLPs) with split hygromycin B resistance in the *C. tropicalis* EG6180 Chr. I and Chr. IV. For homology-directed repair, we used plasmids carrying an insert of interest followed by the N-terminal portion of the hygromycin resistance gene under ribosomal promoter *Ctr-rps-20p*, at a final concentration of 50–60 ng µl^−1^. On day 3 or 4 post-injection, we supplemented plates with 600 µl of 5 mg ml^−1^ hygromycin B. Then, 7–10 days after poisoning, survivors were singled, propagated and screened by PCR and Sanger sequencing to detect successful editing. All guide RNAs and HDR templates as well as plasmids used for SLP injections are listed in Supplementary Tables 4 and 5 and are available upon request. Lines carrying genes of interest in both SLPs were obtained by crossing strains carrying single Chr. I and Chr. IV SLP insertions. Double carriers were screened by PCR and Sanger sequencing to ensure homozygosity of both alleles of interest. To obtain homozygous *kss-3.1-3.4* quadruple-knockout, *kss-3.1; kss-3.2; kss-3.4* triple-knockout and *kss-3.1; kss-3.4* double-knockout lines, additional steps were necessary, as standard screening procedure following CRISPR–Cas gene editing resulted only in heterozygous lines, suggesting that homozygosity in this locus is highly detrimental for the worms. We screened a large number of offspring from heterozygous hermaphrodites to obtain low-frequency homozygous mutant survivors. All transgenic lines and wild isolates used in this study were bulk phenotyped to test if any abnormal phenotypes were present. L4 hermaphrodites were collected, grown overnight and then individually transferred to new plates for a 3-h egg-laying period. Synchronized embryos were transferred to new plates, five to ten embryos per plate, and phenotyped until adulthood. Phenotypes such as embryonic and larval arrest, developmental delay and sterility were assessed. At least 60 embryos were screened per strain. Data can be found in Supplementary Table 1.

### Genetic mapping of *klmt-1/kss-1* and *pzl-1/kss-2*

To map the NIC203 Chr. V TA, we have previously generated a NIL carrying a NIC203 introgression (Chr. V: 1,260,950–1,735,109 Mb) in an otherwise EG6180 genetic background (strain QX2343)^5^. Within this introgression, we identified 176 genes (Supplementary Data 7). Next, we searched for gene pairs in NIC203 that were (1) absent, mutated or highly divergent in EG6180 and (2) tightly linked genetically. From the handful of examples available in nematodes, genes encoding toxins and antidotes tend to have weak or no homology to conserved nematodes genes. Thus, to identify candidates, we defined *C. tropicalis* ‘divergent’ genes as those showing ≤90% sequence identity and ≤80% sequence coverage to their best reciprocal BLAST hit in *C. elegans* and also ≤90% sequence identity and ≤80% sequence coverage between *C. tropicalis* NIC203 and EG6180. These criteria were matched by one pair of genes: *NIC-ORF015419* and *NIC-ORF015420*. To test whether *ORF015419* encoded the toxin, we generated a putative *ORF015419*(−) null allele using CRISPR–Cas (strain INK303). To test whether *ORF015420* encoded the antidote, we generated a putative *ORF015420*(−) null allele in the *ORF015419*(−) genetic background (strain INK422). Both alleles were tested in genetic crosses. Crosses (including genotyping primers) and background phenotypes are described in detail in Supplementary Data 6 and Supplementary Table 1. See the Supplementary Note for more details on Chr. V TA mapping and KLMT-1 expression characterization.

To map Chr. II TA, we used QX2341, NIL carrying a NIC203 introgression (Chr. II: 8,077,521–8,836,894) in an otherwise EG6180 background. Within this introgression, we identified 243 genes (Supplementary Data 8). Using the same divergence and linkage criteria previously applied to the Chr. V TA did not reveal any candidate TA genes. Thus, we reasoned that the susceptible TA allele may simply carry deleterious non-synonymous substitutions. A pilot mRNA-seq differential expression analysis of NIC203 and EG6180 transcriptomes identified *ORF006816*, a gene located within this introgression, as one of the most differentially expressed genes between these two parental strains. To test whether *ORF006816* encoded the toxin, we generated a putative *ORF006816*(−) null allele using CRISPR–Cas (strain INK324). To test whether *ORF006815* encoded the antidote, we generated a putative *ORF006815*(−) null allele in the *ORF006816*(−) genetic background (strain INK485). Both alleles were tested in genetic crosses. Crosses and background phenotypes are described in detail in Supplementary Data 6 and Supplementary Table 1. The susceptible strain, EG6180, has *pzl-1*/*kss-2* TA with the following differences compared with the carrier strain. *EG-ORF006586* encodes EG-KSS-2, which has two substitutions compared with KSS-2. *EG-ORF006587* encodes EG-PZL-1, which has eight non-synonymous substitutions compared with PZL-1 toxin. In addition, there is a transposon insertion in intron 6. DNA and protein sequences can be found in the Supplementary Methods. KSS-1 and KSS-2 pairwise alignment is coloured according to BLOSUM 62 score with conservation colour increment 80 (Fig. 1a).

### Genetic mapping of *hyde-1*/*kss-3*

We ran BLASTN (BLAST v.2.8.1+) search using the *kss-1* sequence as a query against NIC203 and EG6180 genomes. While manually annotating top hits, we noticed that three and four paralogues in NIC203 and EG6180 genomes, correspondingly, were located in the *fars-3* locus, downstream of *zyg-9.2* and upstream of *fars-3*. We named those paralogues *kss-3.1*–*3.4*. We manually characterized every gene in the region between *zyg-9.2* and *fars-3* that was predicted by Funannotate using BLASTN and BLASTP search for homologues, which resulted in annotating 3 *fars-3* homologues in both genomes, including *EG-ORF007390*. Initially, we hypothesized that KSS-3 might be important for regulation of FARS-3 expression. To test this, we tagged *fars-3* with an mScarlet fluorescent tag and knocked out *kss-3.1–3.4* in the FARS-3::mScarlet background. We observed that quadruple knockout results in a severe yet livable phenotype, which was independent of FARS-3::mScarlet expression. An EMS screen performed in the quadruple-knockout strain, along with subsequent crosses between EMS revertant lines and EG6180 parental line, indicated that *fars-3* paralogue *EG-ORF007390* and *kss-3* form a toxin–antidote pair. The *hyde-1*/*kss-3* locus differs between NIC203 and EG6180 isolates. Both possess functioning antidotes: EG6180 has two antidotes, *kss-3.1* and *kss-3.4*, while NIC203 has only *kss-3.4. kss-3.4* in NIC203 and EG6180 share 93% of sequence identity. *NIC-ORF007668* encodes *NIC-hyde-1*, which is a divergent variant of *hyde-1* toxin. *NIC-hyde-1* shares 56% of sequence identity with *hyde-1*. In addition, it has a premature stop codon, leading to a non-functional toxin. DNA and protein sequences can be found in the Supplementary Methods. The multiple sequence alignment of KSS-1 and KSS-3 antidotes is coloured according to the Clustal scheme, using an identity threshold of 51 (Extended Data Fig. 5c).

### EMS-mutagenesis screen for *kss-3* suppressors

An EMS forward mutagenesis screen was performed on the quadruple *kss-3.1*–*3.4* knockout strain to identify phenotype revertants, based on the reasoning that phenotype reversion could result from mutations in potential KSS-3 binding partners. EMS mutagenesis was performed according to the standard protocol^51^ with an increased number of L4s to compensate for the high percentage of lethality in the *kss-3.1*–*3.4* (−) strain. In short, 240 L4s were incubated in 50 mM EMS for 4 h. After extensive washing, an equal number of worms were plated on 12 9-cm plates. Adults were washed off the plates the next day, after confirming that at least 200 F_1_ embryos had been laid per plate. F_1_ gravid adults and F_2_ embryos were collected from plates after 3 days and bleached. F_2_ embryos from each of 12 plates were split into equal proportions and seeded onto four 9-cm plates. Plates were chunked every 4–5 days for 3 weeks. After that, phenotypes were assessed, and all plates were rated based on how long it took for a plate to starve. Based on phenotyping results and to ensure optimal sequencing coverage on the selected Illumina flow cell lane, we chose five WT-like revertant strains (see Supplementary Table 1 for phenotyping results) and proceeded with DNA extraction and Illumina library preparation.

### DNA extraction, library preparation and sequencing

Total DNA was extracted from two freshly starved plates using MasterPure Complete DNA and RNA Purification Kit (Biosearch Technologies). Lysis was performed according to the tissue sample lysis protocol from the manufacturer, with the following changes: 4 µl of proteinase K instead of 1 µl, and incubation for 30 min instead of 15 min. The next part of the protocol, the precipitation of total nucleic acids, was followed without any changes. Library preparation was performed using the Illumina DNA Prep kit (Illumina) according to the manufacturer’s instructions with unique dual indexing. The libraries were assessed using a fragment analyser, pooled in equimolar ratios based on quantitative PCR (qPCR) and sequenced on a NovaSeq X system (Illumina) in single-end read mode for 100 cycles.

### Identification of coding variants

Demultiplexed sequences were aligned to the *C. tropicalis* EG6180 genome using BWA (version 0.7.17) and deduplicated using Sambamba (Samtools version 1.9). Variants were called using the GATK HaplotypeCaller (version 4.0.1.2) with default settings, and variants unique to each revertant strain were extracted with bcftools (version 1.9). Variant effects were revealed using SnpEff (version 5.2c), using a custom *C. tropicalis* EG6180 database, and moderate-/high-effect homozygous variants were extracted for further analysis. Identical pipeline was used to call variants for Arg297Gly variant of KSS-2 in wild isolates from the CaeNDR database^52^.

### RNA extraction and mRNA-seq

Total RNA was extracted from three full 9-cm plates using TRizol chloroform extraction^53^. Phase-lock tubes were used to improve phase separation. To remove DNA, samples were treated with DNase I at 37 °C for 30 min, followed by enzyme inactivation with 2.5 µM EDTA at 65 °C for 10 min. In the case of staged mRNA-seq, RNA was extracted from three different stages: early embryos, late embryos and L4s. Early embryos were obtained by collecting 20,000 synchronized adults per sample before the start of active laying. Adults were additionally gravity sedimented twice to ensure no carry-over of laid embryos. Washed adults were bleached, and the embryonic fraction was immediately snap-frozen. Late embryos fraction was prepared in the same fashion, but embryos were incubated for 6 h after bleaching before snap-freezing. An aliquot of each fraction was inspected at the dissecting microscope. The early embryo fraction includes all developmental stages up to early gastrulation, while the late embryo fraction comprises all stages from the comma stage onwards. For the L4 fraction, 2,000 L4s were collected per sample. Next, 100 ng of purified RNA was used as input for the TruSeq Stranded mRNA kit (Illumina; catalogue no. 20020595), and libraries were prepared according to the manufacturer’s instructions. Libraries were then assessed using a fragment analyser and sequenced on a NovaSeq S4 lane (XP Workflow) for 300 cycles, generating paired-end reads of 150 base pairs each. Library preparation and sequencing was performed by the Vienna Biocenter NGS facility. All samples were run in biological triplicate or quadruplicate for staged mRNA-seq. Transcript quantification and normalization were performed using kallisto^54^ and DESeq2^55^. For mRNA-seq visualization in Extended Data Fig. 1a,d, we aligned mRNA-seq reads to NIC203 genome using HISAT2^56^ (version 2.1.0). Both figures were made with Gviz package^57^ (version 1.52.0).

### RT–qPCR

Total RNA from synchronized embryos was extracted using TRizol chloroform extraction as described earlier. RNA concentrations were measured using the Qubit High-Sensitivity RNA fluorescence kit (Thermo). Absence of genomic DNA contamination in representative RNA samples was confirmed by no amplification signal in PCR reactions with genomic DNA-specific primers. cDNA was prepared with SuperScript III reverse transcriptase (Thermo) using random Oligo(dT) primer. Primers for qPCR were validated with standard curves to ensure amplification efficiency and *R*^2^ values above 0.95. The following primers were used: FW-cdc-42: 5′-CGATTAAATGTGTCGTCGTAGG-3′, and RV-cdc-42: 5′-ACCGATCGTAATCTTCTTGTCC-3′, FW-klmt-1: 5′-CCAACTACTCCAGTGACATCC-3′ and RV-klmt-1: 5′-CCCTCTGAACTCAACAAATCG-3′. *cdc-42* was used as a housekeeping gene. Reverse transcription (RT)–qPCR reactions were prepared with the Luna Universal qPCR and RT–qPCR kit (NEB) and run with an annealing temperature of 58 °C. All samples had three biological and four technical replicates. We used the ∆∆Ct method to calculate the relative fold change.

### Heat shock of *C. tropicalis* early embryos

Heat-shock induction was performed on 5-cm plates with modified NGM. After dissecting gravid adults with insulin syringe needles (29 G) in a drop of M9, embryos were transferred to the plates. After drying for approximately 20 min, plates were transferred to a 37 °C incubator for 40 min, plates were kept with their lids on top as opposed to standard upside-down plate storage, to ensure even warming. The number of embryos was manually counted after heat-shock induction, and plates were kept in a 25 °C incubator overnight. The next day, the number of unhatched embryos was counted, as well as the number of affected L1s, while WT worms were at least L2 stage at this moment. In cases of toxin overexpression, the number of adult survivors was assessed on the second day. The percentage of affected worms was calculated using a sum of non-hatched embryos and affected L1s divided by the total number of embryos used for heat shock for each strain. Overexpression of KLMT-1 and PZL-1 results in mixed embryonic and larval arrest. All experiments were performed in at least triplicates. KLMT-1 heat-shock induction was confirmed by western blot; a weak KLMT-1 signal was still detected in non-induced worms due to leaky expression of the heat-shock promoter at 25 °C (Extended Data Fig. 8h).

### Heat shock of *C. tropicalis* early embryos for microscopy

Gravid adults were washed off from three 9-cm plates, and gravity sedimented twice to ensure no carry-over of laid embryos. Collected adults were bleached, and after two washes with M9 embryos were plated on non-seeded 5-cm plates. Heat-shock induction was performed no earlier than 1 h after collection to ensure that the embryos were completely dry after the washing steps and that the majority of embryos developed enough to have zygotic transcription. After heat-shock plates were incubated at 25 °C for 5.5–6 h, embryos were transferred to slides with agarose pads and imaged on an Axio Imager.Z2 (Zeiss), 20×/0.8 Plan-Apochromat objective. The filter settings used for mCherry were as follows: excitation 545/30 nm, emission 610/75 nm. All experiments were performed in at least duplicates.

### Immunohistochemistry

Gravid adults and laid embryos were washed off plates with M9 medium, followed by bleaching to extract embryos and remove bacteria. The embryo suspension was applied to poly-L-lysine-covered slides (Sigma-Aldrich, P8920). To achieve freeze-cracking of the shells, coverslips were applied, and slides were immersed in liquid nitrogen. Immediately after removal of the coverslips, slides were fixed in ice-cold methanol (15 min), followed by ice-cold acetone (10 min), and rehydrated in descending ethanol concentrations (95%, 70%, 50% and 30% ethanol). Fixed embryos were blocked for 1 h in 4% bovine serum albumin (VWR Life Science, 422351S) in PBS-T with 1% of Tween20 (Sigma-Aldrich, P1379) at room temperature. After blocking, samples were incubated overnight at 4 °C with the primary antibody of choice in blocking solution. The following dilutions were used for primary antibodies: anti-FLAG M2 1:3,000 (Sigma-Aldrich, F1804) and anti-KLMT-1 1:80 (Monoclonal Antibody Facility, Max Perutz Labs). After washing with PBS-T, secondary anti-mouse antibody Alexa Fluor 568 (ThermoFisher Scientific, A-11031) was applied for 1 h at room temperature, and the antibody was diluted 1:3,000 in the blocking buffer. Samples were washed four times with PBS-T. DAPI was added to the third wash (Merck, D9542, 6 ng ml^−1^). Processed embryos were mounted with ProLong Diamond Antifade Mountant (Invitrogen, P3696) and imaged at Axio Imager.Z2 (Zeiss) with a 40×/1.3 Plan-Apochromat Oil objective. The 20×/0.8 Plan-Apochromat objective was used for imaging of large numbers of embryos that were used for fluorescence quantification experiments. The following filter settings were used: DAPI excitation 406/15 nm, emission 457/50 nm, Alexa Fluor 568 excitation 545/30 nm, emission 610/75 nm.

### Time-lapse imaging for KLMT-1::mNG

Synchronized gravid adults were bleached as described earlier, and obtained embryos were immediately transferred to an imaging plastic chamber for inverted microscopy (Lab-TekNunc Chambered Coverglass, 155411). Celldiscoverer 7 (Zeiss) equipped with Hamamatsu Orca Flash 4.0 (pixel size 6.5 μm) camera, LED-module 470 nm (10% intensity), filter set 92 HE and Plan-Apochromat 20×/0.95 objective was used for imaging. Images with a *Z*-stack of 13.8 µm (11 slices) were taken every 5 min for a time course of 10 h. Stacks where the majority of embryos were in-focus throughout the whole time lapse were chosen for quantification. Fluorescence intensity quantification was performed as described below. Representative images of the three strains carrying the *klmt-1::mNG* allele (Extended Data Fig. 4a) were taken on an Axio Imager.Z2 (Zeiss) with the 20×/0.8 Plan-Apochromat objective. The following filter settings were used: excitation 480/40 nm, emission 510LP.

### Fluorescence intensity quantification

Raw 16-bit images were analysed in Fiji. The background was subtracted using a ‘rolling ball’ algorithm with 50 pixels of rolling ball radius for the whole dataset. Next, embryos were selected by the freehand tool, and the same selection mask was used to capture background fluorescence intensity for each embryo. To compare fluorescence intensities between strains, we used the corrected total cell fluorescence (CTCF) parameter (CTCF = integrated density − (area of selected cell × mean fluorescence of background readings)) normalized to the mean fluorescence value for the strain or sample with the lowest fluorescence intensities. Outliers were identified using the ROUT method (*Q* = 1%) and removed from the final graphs and statistical analysis. An outlier test was performed for all datasets apart from time-lapse imaging for KLMT-1::mNG. We chose statistical tests based on data distribution analysis and equality of variance tests. *P* values were adjusted for multiple comparisons if necessary. The half-life of KLMT-1::mNG was estimated on the basis of the average values obtained from all embryos in each dataset.

### KLMT-1 expression and purification

The pETM14 plasmid with an N-terminally 6xHis-tagged KLMT-1 with a 3C-PreScission cleavage site was transformed into the LOBSTR *Escherichia coli* expression strain. This strain was grown in 3 litres of lysogeny broth (LB) up to an optical density at a wavelength of 600 nm (OD_600_) of 0.6 – 0.9 at 37 °C, then put on 4 °C for 30 min and induced with 0.2 mM isopropyl β-D-1-thiogalactopyranoside for overnight expression at 18 °C. The cell pellets were lysed by sonication in denaturing lysis buffer (4 M guanidinium chloride, 50 mM Tris pH 8, 300 mM NaCl, 20 mM imidazole, EDTA-free cOmplete protease inhibitor cocktail (Roche) and in-house 1× benzonase (10,000×, 0.4 mg ml^−1^) (Molecular Biology Service, IMBA). The lysate was cleared by centrifugation at 31,000*g* for 35 min. The supernatant was then loaded onto a 5-ml HisTrap FF (Cytiva). On-column refolding was performed by washing with 30 column volumes (CV) of buffer A (50 mM Tris pH8, 300 mM NaCl and 20 mM imidazole) and an additional 5-CV wash step with 5% buffer B (50 mM Tris pH8, 300 mM NaCl, 500 mM imidazole). The protein was eluted in 50% buffer B. The fractions were checked by in-house Coomassie staining of sodium dodecyl sulfate–polyacrylamide gel electrophoresis (SDS–PAGE) gel. For ion exchange, fractions were pooled, diluted 1/10 in buffer IEX-A (50 mM Tris pH 8 and 0.2 mM dithiothreitol (DTT)) and loaded onto a 6-ml ResourceQ (Cytiva) column. After 30 CV wash in IEX-A, the protein was eluted over a 20 CV gradient to a 100% buffer IEX-B (50 mM Tris pH 8, 1 M NaCl and 0.2 mM DTT). The fractions after ion exchange were either directly run on a Superdex200 Increase 10/300 GL (Cytiva) in SEC buffer (100 mM NaPO4 pH 8, 100 mM NaCl, 5% glycerol, 50 mM arginine, 50 mM glutamate and 0.2 mM DTT) or flash frozen in liquid nitrogen before chromatography. KLMT-1 identity was confirmed by in solution mass spectrometry at 0.1 mg ml^−1^. Twenty microlitres of purified KLMT-1 solution (50 mM Tris pH 8 and 300 mM NaCl) were mixed with 20 µl of 10 M urea in 200 mM ammonium bicarbonate (ABC). Proteins were reduced with 10 mM DTT for 1 h at 37 °C, followed by alkylation with 20 mM Iodoacetamide for 30 min at room temperature in the dark and 30 min of quenching with another 5 mM DTT. Proteins were digested with 500 ng of lysyl endopeptidase (Lys-C, Fujifilm Wako Pure Chemical Corporation) for 2 h at 37 °C. Subsequently, the solution was diluted to 2 M urea with 100 mM ABC, and the proteins were digested with 500 ng trypsin (Trypsin Gold, Promega) at 37 °C overnight. The digest was acidified by addition of trifluoroacetic acid (Pierce) to 1%.

### Monoclonal antibody generation against KLMT-1

Mouse monoclonal antibody against full-length KLMT-1 (clone 1A3-3E5) was generated at the Max Perutz Laboratories Monoclonal Antibody Facility. In brief, BALB/c mice were immunized with recombinant, His-tagged KLMT-1. Splenocytes of the best-responding mouse according to serum screening by western blot were fused with X63-Ag8.653 mouse myeloma cells, and hybridoma clones were established by hypoxanthine-aminopterin-thymidine (HAT) selection. Eight days after fusion, hybridoma supernatants were screened by western blot for the presence of KLMT-1 specific antibodies, and antibody-secreting hybridoma single-clone 1A3-3A5 was established. The monoclonal antibody was validated using NIC203 (positive control) and EG6180 (negative control) protein lysates.

### Worm protein lysate preparation and western blot

Two mixed-stage plates were used to prepare protein lysate if not stated otherwise. For heat-shock induction verification (Extended Data Fig. 8h), two mixed-stage 9-cm plates were subjected to a 1-h heat shock at 37 °C, followed by a 3-h recovery at 25 °C before protein extraction. Worms were washed off plates with M9, washed twice to remove bacteria, pelleted and resuspended in ice-cold lysis buffer: 50 mM HEPES pH 7.4, 150 mM NaCl, 2 mM MgCl_2_, 0.05% IGEPAL, 10% glycerol, protease inhibitors (Roche, 11836153001) and in-house 1× benzonase (10,000×, 0.4 mg ml^−1^) (Molecular Biology Service, IMBA). After snap-freezing, samples were lysed by sonication using a Bioruptor (UCD-200, Diagenode) for three cycles of 30 s ON/30 s OFF over 8 min at high energy in an ice-water bath, followed by centrifugation to collect the clarified supernatant. The protein concentration was quantified using a Bradford assay (Thermo Scientific, 23238) and adjusted to 1.5 mg ml^−1^ with lysis buffer. Samples were incubated with an SDS loading buffer for 10 min at 95 °C and loaded onto NuPAGE Bis-Tris 4–12% gel (Invitrogen). After electrophoresis, samples were transferred to 0.45 µm polyvinylidene fluoride membrane (Thermo Scientific, 88518) and blocked with 4% non-fat milk in TBS-T with 1% of Tween20 (Sigma-Aldrich, P1379) for 1 h at room temperature. Blocked membranes were incubated with primary antibodies in blocking buffer overnight at 4 °C. Following primary antibody dilutions were used: anti-FLAG M2 1:2,000 (Sigma-Aldrich, F3165), anti-actin 1:3,000 (Abcam, ab13772), anti-tubulin 1:3,000 (Sigma-Aldrich, 05-829-AF647) or anti-KLMT-1 1:60 (Monoclonal Antibody Facility, Max Perutz Labs). After incubation, membranes were washed with TBS-T, followed by the incubation with respective HRP-conjugated secondary antibodies: anti-mouse (1:10,000, Invitrogen, G-21040) or anti-rabbit (1:10,000, Jackson Immuno, 111-035-045). Anti-α-tubulin antibody is conjugated with Alexa Fluor 647, so it does not require a secondary antibody. Substrate detection was performed using ECL reagent (Cytiva, RPN2106) and imaged with ChemiDoc MP (Bio-Rad). Membranes were stripped for 1 h before reprobing if necessary (Thermo Scientific, 21059). Alpha tubulin or actin staining was used as a loading control for every western blot.

### Co-IP–MS

Worm pellets were resuspended in 200 µl cold buffer A (50 mM HEPES pH 7.3, 150 mM NaCl, 10% glycerol, 0.05% NP-40, 1 mM EDTA; freshly supplemented with 2× cOmplete EDTA free (Roche) and 1 mM Tris(2-carboxyethyl)phosphine (TCEP)). Worms were lysed using a Bioruptor Plus (Diagenode) with the following settings: three repeats of 30 s ON/30 s OFF for 8 min at high energy in an ice-water bath. Lysates were clarified by centrifugation at 18,000*g* for 20 min at 4 °C. Total protein concentration was quantified and using the Bradford assay. At this stage, an input sample was collected in the SDS buffer for later analysis. Next, 1 mg of clarified protein extract was incubated with 15 µl of settled anti-FLAG M2 magnetic agarose beads (Millipore, M8823), previously equilibrated in buffer A. Incubation was carried out while rotating for 3 h at 4 °C. Subsequently, an unbound sample was collected in SDS buffer for later analysis. Beads were then washed four times in 1 ml buffer A, followed by six washes in buffer B (50 mM HEPES pH 7.3 and 150 mM NaCl). Twenty per cent of beads were collected in the SDS buffer for later analysis. The remaining 80% were snap-frozen and stored at −20 °C until further processing with on-bead digestion for mass spectrometry. The co-IP experiment was validated by analysing input, unbound and IP samples by silver staining as well as western blotting using standard protocols. For on-bead digestion, beads were resuspended in 50 µl of 100 mM ABC, supplemented with 400 ng of lysyl endopeptidase (Lys-C, Fujifilm Wako Pure Chemical Corporation) and incubated for 4 h on a thermo-shaker with 1,200 rpm at 37 °C. The supernatant was transferred to a fresh tube and reduced with 0.5 mM Tris 2-carboxyethyl phosphine hydrochloride (Sigma) for 30 min at 60 °C and alkylated in 3 mM methyl methanethiosulfonate (Fluka) for 30 min at room temperature, protected from light. Subsequently, the sample was digested with 400 ng trypsin (Trypsin Gold, Promega) at 37 °C overnight. The digest was acidified by addition of trifluoroacetic acid (Pierce) to 1%. A similar aliquot of each sample was analysed by liquid chromatography–tandem MS (LC–MS/MS).

### Nano LC–MS/MS

The nano HPLC system (either the UltiMate 3000 RSLCnano or the Vanquish Neo UHPLC system) was coupled to an Orbitrap Exploris 480 mass spectrometer, equipped with a FAIMS Pro interface and a Nanospray Flex ion source (all parts Thermo Fisher Scientific). Peptides were loaded onto a trap column (PepMap Acclaim C18, 5 mm × 300 μm inner diameter, 5-μm particles, 100 Å pore size, Thermo Fisher Scientific) at a flow rate of 25 μl min^−1^ using 0.1% trifluoroacetic acid as mobile phase. After loading, the trap column was switched in line with the analytical column (PepMap Acclaim C18, 500 mm × 75 μm inner diameter, 2 μm, 100 Å, Thermo Fisher Scientific). Peptides were eluted using a flow rate of 230 nl min^−1^, starting with the mobile phases 98% A (0.1% formic acid in water) and 2% B (80% acetonitrile, 0.1% formic acid) and linearly increasing to 35% B over the next 60 min (for in gel digests) or 120 min. This was followed by a steep gradient to 95% B within 1 min, held for 6 min, and then decreased over 2 min to the starting conditions of 98% A and 2% B, for equilibration at 30 °C. The Orbitrap Exploris 480 mass spectrometer was operated in data-dependent mode, performing a full scan (*m*/*z* range 350–1,200, resolution 60,000, normalized automatic gain control (AGC) target 300%) at three different compensation voltages (CV −45 V, −60 V and −75 V), followed by MS/MS scans of the most abundant ions for a cycle time of 0.9 s for each. MS/MS spectra were acquired using an isolation width of 1.2 *m*/*z*, normalized AGC target 200%, minimum intensity set to 25,000 or 50,000 (gel digests), higher-energy collisional dissociation (HCD) collision energy of 30%, maximum injection time of 100 ms and resolution of 30,000. Precursor ions selected for fragmentation (including charge states 2–6) were excluded for 10 s (gel digests) or 45 s. The monoisotopic precursor selection (MIPS) mode was set to peptide, and the exclude isotopes feature was enabled. Proteins with at least two peptides identified were used for volcano plots.

### Co-expression and purification of KSS-1::Strep–KLMT-1–SKR-1

The coding sequences were cloned from cDNA into a baculovirus expression vector pGB-Dest^58^. Sf9 cells were transfected and incubated at 27 °C for protein expression, and the cells were collected 4 days after proliferation arrest. Collected samples were stored at −70 °C until purification. One litre of cells were thawed on ice and resuspended in lysis buffer consisting of 1× PBS, 50 mM sodium citrate, 1× benzonase (10,000×, 0.4 mg ml^−1^) (Molecular Biology Service, IMBA), EDTA-free cOmplete protease inhibitor cocktail (Roche), 200 µl BioLock (IBA) and 0.5 mM TCEP. The cells were centrifuged for 40 min at 50,000*g* at 8 °C. For affinity purification, the supernatant was loaded onto a 5 ml StrepTrapXT (Cytiva). The column was washed with 20 CV of PBS supplied with 5 mM ATP and 10 mM MgCl_2_. To avoid copurification of Sf9 chaperones, an additional manual wash was carried out as follows: (1) the flow-through from the StrepTrapXT was retrieved, boiled at 95 °C for 5 min and spun down at 21,000*g* for 10 min; (2) the supernatant from this step was diluted to 1 mg ml^−1^ with wash buffer (PBS, 5 mM ATP and 10 mM MgCl_2_) and 3 CV were manually loaded on to the column; (3) the column was incubated with this solution at room temperature for 10 min to allow the chaperones to bind to misfolded proteins; and finally the column was reattached to the fast protein liquid chromatography (FPLC). After 15 CV of PBS wash, the complex was eluted with PBS and 50 mM biotin with 0.5 ml min^−1^ in the up-flow mode. Individual bands were digested in gel (see below), and their identity was confirmed by mass spectrometry. When examining the fractions from the affinity purification, we noticed that the first ATP/Mg^2+^ wash contained proteins washed off the column. Although present at low concentrations, this protein fraction appeared pure, with the two main bands corresponding KSS-1::Strep and SKR-1. The wash fraction, without carry-over from the flow-through, was dialysed in two steps: first against 1 litre of 20 mM Tris pH 7.5 with 150 mM NaCl, and then against 1 litre of 20 mM Tris pH 7.5 with 50 mM NaCl to remove ATP and NaCl before anion exchange. This sample was then loaded onto a 6-ml ResourceQ column (Cytiva) and eluted using increasing NaCl concentrations. Fractions containing both KSS-1::Strep and SKR-1 were then concentrated and loaded onto a Superdex 200 Increase 10/300 GL column (Cytiva) (Fig. 2g) using 20 mM Tris pH 7.5 and 150 mM NaCl as a running buffer. The main peak fractions were pooled again, concentrated and filtered for size exclusion chromatography with multi-angle light scattering (SEC-MALLS, Fig. 2g, inset) using the same running buffer.

### In-gel digest

Coomassie-stained gel bands were cut into 2–3 mm pieces, transferred to 0.6-ml tubes and incubated with different solutions by shaking for 10 min at room temperature, followed by removal of the supernatant as follows: gel pieces were washed with 200 µl 100 mM ABC, destained by two repeated rounds of shrinking in 200 µl 50% acetonitrile in 50 mM ABC and reswelling in 200 µl 100 mM ABC. Gel pieces were shrunk with 100 µl acetonitrile before being reduced with 100 µl of 6 mM DTT in 100 mM ABC by incubation at 57 °C for 30 min and alkylated with 100 µl of 28 mM methyl methanethiosulfonate in 100 mM ABC by incubation at room temperature for 30 min. Wash steps were repeated as described for destaining, and gel pieces were briefly dried in a SpeedVac after the final shrinking step. Gel pieces were soaked in 12.5 ng μl^−1^ trypsin in ABC for 5 min at 4 °C. Excess solution was removed, ABC was added to cover the pieces and samples were kept overnight at 37 °C. The supernatant containing tryptic peptides was transferred to a fresh tube, and gel pieces were extracted by addition of 20 µl 5% formic acid and sonication for 10 min in a cooled ultrasonic bath. This step was performed twice. All supernatants were unified. A similar aliquot of each digest was analysed by LC–MS/MS

### Proteomic data processing

For peptide identification, the RAW files were loaded into Proteome Discoverer (version 2.5.0.400, Thermo Scientific). All MS/MS spectra were searched using MSAmanda v2.0.0.19924^59^. The peptide mass tolerance was set to ±10 ppm and fragment mass tolerance to ±10 ppm; the maximum number of missed cleavages was set to 2, using tryptic enzymatic specificity without proline restriction. Peptide and protein identification was performed in two steps. For an initial search, the RAW files were searched against a combined database for *C. tropicalis* (UniProt and Wormbase; 27,687 sequences; 10,300,894 residues) or custom database containing predicted open reading frames of EG6180 isolate, based on Funannotate prediction (25,392 sequences; 9,472,164 residues); in addition, we searched against the UniProt reference database for *E. coli* (4,360 sequences; 1,354,438 residues), supplemented with common contaminants and sequences of tagged proteins of interest using β-methylthiolation of iodoacetamide derivative, respectively, on cysteine as a fixed modification. The result was filtered to 1% false discovery rate at the protein level using the Percolator algorithm^60^ integrated in Proteome Discoverer. A subdatabase of proteins identified in this search was generated for further processing. For the second search, the RAW files were searched against the created subdatabase using the same settings as above and considering the following additional variable modifications: oxidation on methionine, deamidation on asparagine and glutamine, phosphorylation on serine, threonine and tyrosine, glutamine to pyro-glutamate conversion at peptide N-terminal glutamine and acetylation on the protein N terminus. The localization of the post-translational modification sites within the peptides was performed with the tool ptmRS, based on the tool phosphoRS^61^. Identifications were filtered again to 1% false discovery rate at the protein and peptide-spectrum match (PSM) level; in addition, an Amanda score cut-off of at least 150 was applied. Match-between-runs was applied to peptides with high-confidence peak areas that were identified by MS/MS spectra in at least one run. Protein areas were computed in IMP-apQuant^62^ by summing up unique and razor peptides. Resulting protein areas were normalized using iBAQ^63^, and sum normalization was applied for normalization between samples. Proteins were filtered to be identified by a minimum of two PSMs in at least one sample, and quantified proteins were filtered to contain at least three quantified peptide groups. Statistical significance of differentially expressed proteins was determined using limma^64^. In cases of 3xFLAG::KSS-1 pull-down, we used a log_2_(fold change) cut-off equal to −4 for the volcano plot (Fig. 2h). As a result, EG-ORF005520 with log_2_(fold change) equal to −10.727 was removed from the volcano plot (10.2 kDa protein with 2 quantified peptides and no identified homology). The same protein was identified in the 3xFLAG::KSS-2 pull-down experiment, with log_2_(fold change) of −10.516 (Extended Data Fig. 3f).

### Annotation of KSL proteins and phylogenetic reconstruction

To annotate genes homologous to the *kss* antidotes across *Caenorhabditis* species, we first constructed a HMM to represent the conserved regions of a small hand-curated set of *C.tropicalis* KSS antidotes and homologous proteins from *C. tropicalis* and *C. wallacei*. Based on preliminary analyses, we prioritized species within the *Elegans* group, focusing on those with chromosome-level genome assemblies^65–68^. These included: *C. elegans* (N2, WBcel235), *C. tropicalis* (EG6180), *C. brenneri* (CFB2252), *C. briggsae* (AF16), *C. doughertyi* (JU1771, nxCaeDoug1.1), *C. inopinata* (NKZ35, Sp34_v7), *C. latens* (PX534, ASM225923v3), *C. remanei* (PX506, CRPX506), *C. sinica* (JU800, nxCaeSini1.1), *C. wallacei* (JU1904, nxCaeWall1) and *C. zanzibari* (JU2190, nxCaeZanz1.1). Also, we included *C. sulstoni* (JU2788, nxCaeSuls) and *C. japonica* (DF5081, nxCaeJapo1.1) as outgroups. We searched genomes with tblastn (BLAST v2.14.0), applying an e-value threshold of 1 × 10^−5^ to identify potential gene regions. After identifying these regions, we extended them by 2,000 nucleotides upstream and downstream, and merged overlapping regions into longer fragments using bedtools (v2.30.0). We predicted the gene structures with genewise (v2.4.1), using these fragments and the HMM. We named each gene on the basis of its chromosomal location in the format ctr-ksl-[number] (for example, *ctr-ksl-29*). Next, we generated a multiple sequence alignment from the predicted genes using mafft-linsi (v7.427), optimizing for sequences with large insertions and deletions. To maintain alignment consistency, we replaced stop codons with the placeholder amino acid ‘X’. We then generated nucleotide codon alignments informed by the protein alignment using revtrans (v1.4) with the -readthroughstop option. We filtered the resulting nucleotide multiple sequence alignment to retain only nucleotide positions covered by at least 10% or 80% of the sequences, and we replaced poorly covered positions with gaps. Finally, we constructed a maximum-likelihood phylogenetic tree using IQ-TREE (v2.3.6) with default parameters, allowing us to infer the evolutionary relationships among the *kss*/*ksl* gene family. An updated HMM including all 560 proteins identified in the first annotation was used to search for new KSL genes; however, only 7 additional genes were identified (not included), suggesting that the original search was largely exhaustive. The KSL tree in Fig. 5a was generated using a subset of hand-curated proteins that were most closely related to the KSS antidotes. A representative KSL from C. *doughertyi* (KSL-30) was used as an outgroup. Proteins were aligned using MAFFT v7 with default settings, and the phylogenetic tree was constructed using IQ-TREE (v2.3.6).

### Phylogenetic reconstruction of *fars-3* and its toxic paralogues

We retrieved and manually curated orthologues of *fars-3* from the Wormbase ParaSite WBPS19 (WS291) database^69,70^. To minimize artefacts in the multiple sequence alignment, we excluded regions of the toxin sequences that were predicted to be intrinsically disordered based on IUPred2A^71^, had low pLDDT scores based on AlphaFold2 predictions and showed poor sequence identity compared with *C. tropicalis fars-3*. If a region was predicted to be disordered but had substantial homology, it was kept for further analysis. Based on these criteria, we retained KLMT-1 [AAs: 49–404] and HYDE-1 [AAs: 49–567]. For PZL-1, a chimera consisting of three distinct gene regions—*mec-15*, *zyg-9.2* and *fars-3*—we included only the segment homologous to *fars-3*, identified through sequence conservation and structural homology predicted by AlphaFold2. This led to the inclusion of PZL-1 [AAs: 642–788]. All DNA sequences were aligned using MAFFT v7^72^ with default settings, and the resulting phylogenetic tree was constructed using IQ-TREE (v2.3.6)^73^. All pairwise alignments for *fars-3* and *fars-3*-derived toxins are coloured according to the BLOSUM 62 score with conservation colour increment 80 (Extended Data Figs. 1h and 6d).

### De novo protein structure prediction and analysis

We predicted protein structures using AlphaFold2^74^. AlphaFold2 and Alphafold2-multimer were run using the ColabFold (v1.4.0) notebook implementation in Google Colaboratory^75^. For multiple sequence alignment, we selected the MMseqs2 option. Models were ranked based on pLDDT, and only the best ranked out of five models was selected for further analysis. We searched for structurally homologous proteins in PDB using the DALI^15^ or Foldseek servers^76^. Graphics were generated using PyMol (The PyMOL Molecular Graphics System v2.5, Schrödinger). Protein alignment statistics for the selected pairs were generated using the super algorithm for protein pairs with high sequence identity and cealign for protein pairs with low sequence identity. We estimated and plotted the evolutionary conservation of *C. tropicalis* KSL residues using the ConSurf server^77^.

### Statistical analysis and reproducibility

GraphPad Prism 10 was used for data analysis and representation for all crosses, phenotyping, heat-shock overexpression experiments and fluorescence intensity quantification. Figures and figure legends provide descriptions of the statistical tests, error bars and sample sizes. For all tests, 0.05 was set as the significance threshold. For each experiment with multiple comparisons, we conducted the Shapiro–Wilk or Kolmogorov–Smirnov normality test. If the distribution was Gaussian, a Brown–Forsythe analysis of variance (ANOVA) was used; if not, a Kruskal–Wallis test was applied. Both were followed by appropriate multiple comparisons tests. Proteomic data and mRNA-seq processing was described earlier. Microscopy experiments where only representative microphotographs are shown were performed at least twice, with the following exceptions: in Fig. 1h, heat-shock experiments were performed multiple times with the identical outcome, but larvae were imaged once; smFISH gonad staining (Supplementary Fig. 2b) was also performed once as a complementary experiment to smFISH in embryos and KLMT-1::mNG localization in the adult germline. KLMT-1–KSS-1–SKR-1 and KSS-1–SKR-1 complexes were reproducibly purified (Fig. 2f,g). All western blots were performed at least twice with one exception—Extended Data Fig. 4f; in this case, samples were loaded in biological triplicates.

### Reporting summary

Further information on research design is available in the Nature Portfolio Reporting Summary linked to this article.

## Supplementary information

Supplementary Information Supplementary Note, Figs. 1–5, Methods and Tables 1–6.

Reporting Summary

Supplementary Data 1 In vivo interactome of KSS-1 revealed by IP–MS; data used for KSS-1 volcano plot.

Supplementary Data 2 In vivo interactome of KSS-2 revealed by IP–MS; data used for KSS-2 volcano plot.

Supplementary Data 3 EMS-derived mutations identified in suppressor lines.

Supplementary Data 4 Sequences of the 560 KSL proteins identified in different *Caenorhabditis* species.

Supplementary Data 5 *C. elegans* proteins with predicted structural homology to KSS-1.

Supplementary Data 6 Table containing raw data and summary for all genetic crosses; includes genotyping primers sequences.

Supplementary Data 7 List of candidate TA genes within the Chr. V NIL introgression strain (NIC203 to EG6180).

Supplementary Data 8 List of candidate TA genes within the Chr. II NIL introgression strain (NIC203 to EG6180).

Supplementary Data 9 Source data for Supplementary Figs. 1–3.

## Source data

Source Data Fig. 1 Raw data for heat shock and percentages used for statistical analysis.

Source Data Fig. 2 Raw data for heat shock and percentages used for statistical analysis; statistical source data for immunofluorescence quantification.

Source Data Fig. 2 Unmodified Coomassie-stained gels.

Source Data Fig. 3 Statistical source data for crosses and phenotyping.

Source Data Fig. 4 Statistical source data for crosses and immunofluorescence quantification.

Source Data Extended Data Fig. 1 Statistical source data for crosses.

Source Data Extended Data Fig. 1 Unmodified western blot membranes.

Source Data Extended Data Fig. 4 Statistical source data for crosses and qPCR.

Source Data Extended Data Fig. 4 Unmodified western blot membranes.

Source Data Extended Data Fig. 5 Statistical source data for immunofluorescence quantification.

Source Data Extended Data Fig. 6 Statistical source data for crosses.

Source Data Extended Data Fig. 8 Raw data for heat shock and percentages used for statistical analysis; statistical source data for immunofluorescence quantification and crosses.

Source Data Extended Data Fig. 8 Unmodified western blot membranes.

## Data Availability

mRNA-seq reads are available in the SRA database (PRJNA1159832). NIC203 and EG6180 genome annotation and predicted open reading frames, MS results for KLMT-1 and KLMT-1-KSS-1::Strep-SKR-1 complex purification as well as KSS-1::FLAG and KSS-2::FLAG co-IP/MS results are available via Zenodo at 10.5281/zenodo.17456948 (ref. ^78^). The Article is accompanied by Supplementary Information, Supplementary Data 1–9 and Source data files (source data for supplementary figures can be found in Supplementary Data 9 and Supplementary Fig. 5). Source data are provided with this paper.
