# Supplementary material for: Recurrent evolution of selfishness from an essential tRNA synthetase in Caenorhabditis tropicalis
Source: Nat Ecol Evol. 2025 Nov 17;9(12):2374–90. doi: 10.1038/s41559-025-02894-2 (PMC12680543; doi:10.1038/s41559-025-02894-2)
Supplement: Supplementary file 1 — Supplementary Note, Figs. 1–5, Methods and Tables 1–6. [file 41559_2025_2894_MOESM1_ESM.pdf]

nature ecology & evolution

Supplementary information https://doi.org/10.1038/s41559-025-02894-2

Recurrent evolution of selfishness from an essential tRNA synthetase in *Caenorhabditis tropicalis*

In the format provided by the authors and unedited

Table of Contents

Supplementary Notes
Supplementary Figures 1-5
Supplementary Methods
Supplementary Tables 1-6
Supplementary References

Supplementary Notes

Mapping of the Chr. V TA.

To facilitate the identification and characterization of NIC203 TAs, we took advantage of near-isogenic lines (NILs) each carrying a single TA (NIC203 introgression) in an otherwise EG6180 parental background. In crosses between the Chr. V TA NIL and the EG6180 parental line, ~25% of the F_2 progeny dies during embryonic development or shortly after hatching (L1 stage). In agreement with the TA model, the vast majority of these affected individuals were homozygous carriers of the EG6180 allele (91.3%, n=92; Supplementary Fig. 1a, records for all crosses available in Supplementary data 6). To identify the genes underlying this incompatibility, we leveraged Nanopore long-read de novo genome assemblies and RNA-seq expression data to define a list of candidate loci within the ~474 kb (1.26–1.74 Mb) NIC203 introgression. Among the 176 genes we found within this region, we sought gene pairs that were either (i) absent, mutated or divergent in EG6180 and (ii) in tight genetic linkage. These criteria were matched by one pair of genes: *NIC-ORF015419* and *NIC-ORF015420* (Supplementary data 7).

Since toxins are typically expressed at higher levels than antidotes (Supplementary Fig. 1b), we hypothesized that *ORF015419* is the toxin-encoding gene. To test this, we first generated an *ORF015419* knockout allele in the Chr. V NIL background using CRISPR/Cas. The mutation introduced a frameshift and premature stop codon resulting in a truncated protein (Extended Data Fig. 1c). *ORF015419(-)* mutants were phenotypically wild-type (95% WT, n=100) indicating that *ORF015419* is dispensable for the normal development of *C. tropicalis*. To test if *ORF015419* is the toxin, we crossed *ORF015419(-)* NIL hermaphrodites to EG6180 males and checked whether the TA was still active. We observed background levels of embryonic lethality among the F_2 progeny (2%, n=200) in contrast to the ~25% affected progeny in the wild type cross (31%, n=200) (Extended Data Fig. 1c). Furthermore, F_2 progeny homozygous for the susceptible allele (EG/EG) were recovered at the expected Mendelian ratio and were perfectly viable and fertile (26.5%, n=200). Thus, we concluded that *NIC-ORF015419* codes for the toxin and named the novel locus *klmt-1* (for *Killer of embryos and Larvae Maternal Toxin;* pronounced *klimt)*.

Next, we determined that the gene immediately upstream of *klmt-1*, *NIC-ORF015420*, encodes its antidote, which we named *kss-1* (for *Killer rescue by zygotically expreSSed gene*; pronounced *kiss*). If *klmt-1* and *kss-1* make up the TA, then we would expect the *klmt-1(-) kss-1(-)* double mutant haplotype to phenocopy a susceptible EG6180 allele, which lacks *klmt-1/kss-1* TA. To show this, we mutated *kss-1* in the background of the mutant toxin using CRISPR/Cas (Extended Data Fig. 1c) and then crossed *klmt-1(-) kss-1(-)* double mutant hermaphrodites to wild type NIL males. Consistent with *kss-1* being

the antidote, we observed ~25% embryonic/L1 lethality among the F_2 progeny (24.5%, n=200), and all genotyped individuals homozygous for the double mutant allele were embryonic or larval lethal (n=17). As a control, only background levels of lethality were observed in the double mutant NIL parental strain (4%, n=100). Thus, *klmt-1/kss-1* is a novel maternal-effect TA.

The susceptible strain, EG6180, carries remnants of *klmt-1/kss-1* TA. *EG-ORF014892* is a pseudogenized version of *klmt*-1. Using Mauve[56], we identified an inversion that beings within exon 3 of *klmt-1* likely resulting in a null allele (Supplementary Fig. 1c). *EG-ORF014894* codes for EG-KSS-1, which is predicted to be 387 amino acids long protein. EG-KSS-1 expression is lower compared to KSS-1 antidote in NIC203, moreover it has 15 substitutions, and 2 amino acids deletion compared to the NIC203 KSS-1 antidote, which renders EG-KSS-1 non-functional (Supplementary Fig. 1b-c). DNA and protein sequences for *EG-ORF014894* can be found in the Supplementary methods.

The *klmt-1* toxin mRNA and protein are expressed in the adult gonad and during embryogenesis.

In agreement with its role as a maternal-effect toxin, we detected *klmt-1* mRNA expression in NIC203 embryos shortly after fertilisation and before zygotic genome activation, which, based on *C. elegans* data, starts at the 4-cell stage[1] (Supplementary Fig. 2a). We also observed *klmt-1* expression in the adult gonad, in both cases using single-molecule fluorescent in situ hybridization (smFISH) (Supplementary Fig. 2b). The presence of the transcript both in the gonad and in the early embryos shortly after fertilization suggests that *klmt-1* mRNA is maternally loaded into the zygote. At the comma stage, when embryo starts to elongate and fold, *klmt-1* mRNA was only detectable in a pair of cells corresponding to the Z2 and Z3 germline precursors, which were identified by using *pgl-1*, marker of germ-cell-specific granules in nematodes[2] (Supplementary Fig. 2c). Quantification of transcript abundance also supports the observed pattern: mRNA molecules are abundantly present in early embryos and only limited number of transcripts is detected during elongation stages – final stages of embryonic development. As a negative control, we did not detect *klmt-1* mRNA in EG6180 embryos and gonad (Supplementary Fig. 2a-d).

To study the localization of KLMT-1 at the protein level, we introduced a C-terminal 3xFLAG tag at the *klmt-1* endogenous locus in the NIL background using CRISPR/Cas homology-mediated repair. The resulting KLMT-1::3xFLAG toxin could be readily detected by western blot and closely matched its predicted molecular weight of 59.3 kDa (Extended Data Fig. 1g). To test whether KLMT-1::3xFLAG was active, we crossed *klmt-1::3xflag* NIL hermaphrodites to EG6180 males and scored their F_2 progeny. In agreement with an active *klmt-1/kss-1*, we observed 24.5% of affected F_2 progeny (n=200) and the majority of the genotyped affected progeny were homozygous EG/EG embryos (87.5%, n=24), indicating that the tag does not interfere with the activity of the toxin (Supplementary Fig. 2e). Immunofluorescence staining revealed that KLMT-1::3xFLAG protein is maternally loaded into eggs

prior to fertilization, because, as *klmt-1* mRNA, it was detected at 2-cell stage prior to zygotic genome activation (Supplementary Fig. 2f). KLMT-1 protein levels quickly declined during embryogenesis and could only be detected in the germ cell precursor cells following the mid gastrulation stage (Supplementary Fig. 2g-h). We verified KLMT-1::3xFLAG expression patten using KLMT-1 monoclonal antibody, which recognised WT KLMT-1 as well as the tagged KLMT-1 (Supplementary Fig. 2i). We concluded that *klmt-1,* both as mRNA and protein, is maternally deposited into the embryos, and its expression is restricted to germ-cell precursors at later developmental stages.

Maternal KLMT-1 is sufficient to poison embryos

Since both *klmt-1* mRNA and protein are maternally loaded into eggs, we next asked whether maternal KLMT-1 protein is sufficient to poison embryos. To do this, we first expressed KLMT-1 in *E. coli*, purified it using a 6xHis-tag, and confirmed its identity using liquid chromatography mass spectrometry (LC-MS) (Supplementary Fig. 3a). We then co-injected the purified KLMT-1 protein and a plasmid-encoded fluorescent marker into the gonad of EG6180 hermaphrodites and characterized in detail their progeny following selfing. Analogously to the toxicity caused by the *klmt-1/kss-1* TA in genetic crosses, injection of purified KLMT-1—but not the vehicle buffer—caused high levels of embryonic and L1 lethality among their F_1 progeny (77.7%, n=628, Supplementary Table 6, Supplementary Fig. 3b). To test whether the toxicity was specifically caused by KLMT-1, we injected the purified toxin into a line overexpressing KSS-1 from a single copy transgene. We observed only background levels of embryonic lethality in the presence of its antidote (2.9%, n=239, Supplementary Table 6) indicating that KLMT-1 was responsible for the toxicity (Supplementary Fig. 3b). Despite the drastic difference in F_1 survival following KLMT-1 injection, injected mothers showed no obvious physiological problems or differences in the number of eggs laid (Supplementary Fig. 3b), suggesting that KLMT-1 does not affect the fertility of adult mothers. We conclude that maternally deposited KLMT-1 is sufficient to poison embryos.

On the evolution of *klmt-1/kss-1*, *pzl-1/kss-2*, and *hyde-3/kss-3.1,4* from *fars-3*

The conclusion that *pzl-1/kss-2* was the earliest toxin-antidote (TA) pair to emerge is based on three independent lines of evidence: First, the phylogeny of all *fars-3* paralogs and associated toxins indicates that the earliest duplication event involved *fars-3* and *pzl-1* (Fig. 3f). Second, an independent phylogeny of KSS antidotes and related F-box proteins indicates that the first duplication event gave rise to *kss-2* and the common ancestor of *kss-3* and *kss-1*, suggesting that *kss-2* is the oldest antidote (Fig. 5a). Third, *PZL-1* is the most derived toxin in terms of gene architecture, incorporating sequences from two genes (zyg-9 and *mec-15*) in addition to *fars-3*, which is consistent with a longer evolutionary history (Fig 1c,f). Contrast this with HYDE-1 that shows the highest structural and sequence similarity to FARS-3 and is also in tight genetic linkage to the *fars-3* locus, supporting its emergence through a more recent

duplication event (Fig 3). Together, these findings support the conclusion that *pzl-1/kss-2* represents the earliest TA pair to have emerged from the *fars-3* lineage.

We speculate that the ancestral KSS antidote (Anc-KSS) initially recognized a structured domain of FARS-3. After the duplication of the *fars-3*/Anc-KSS module, the binding interface of KSS-1 co-evolved with KLMT-1, both to accommodate changes that enhanced toxicity and to ensure specificity among related toxins. Since KSS antidotes act by recruiting the SCF complex to ubiquitinate their targets, the exact binding interface may not be critical, as long as the toxin is bound and marked for degradation. This functional flexibility likely allows binding interfaces to diverge and evolve over time, potentially explaninig how KSS-1 binds a disordered region in KLMT-1 that is not present in the ancestral FARS-3.

Supplementary Figures

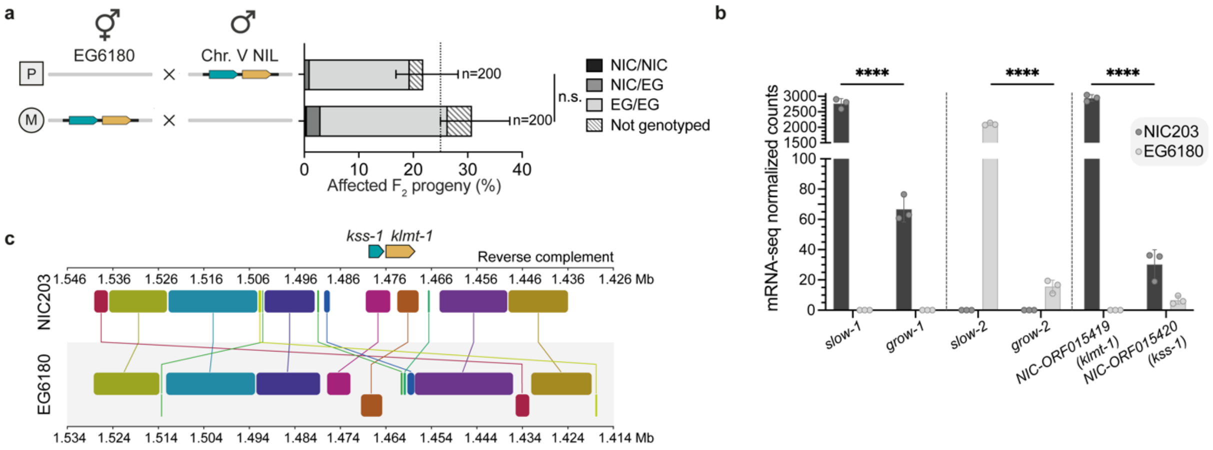

Supplementary Fig. 1. Additional information on genetic mapping of the *klmt-1/kss-1* toxin-antidote element. (a) The TA is equally active when inherited either maternally (M) or paternally (P) (P = 0.0538, two-sided Fisher's exact test). Error bars – mean with 95% CI, hybrid Wilson/Brown method. **(b)** Quantification of mRNA expression levels for two previously described *C. tropicalis* TAs, *slow-1/grow-1* and *slow-2/grow-2*, as well as a newly identified TA, *klmt-1/kss-1*. Samples correspond to mRNA-seq data from NIC203 and EG6180 gravid young adults in biological triplicates. Toxin coding genes have higher expression levels compared to antidote-coding genes (two-sided unpaired t-test; $P < 0.0001$ in all cases). Error bars – mean ± SD. **(c)** Similarity profile of NIC203 and EG6180 *klmt-1/kss-1* locus. Blocks located below the center line indicate inversed regions. NIC203 was used as a reference for Mauve alignment, alignment parameters – seed 15, LCB weight 388.

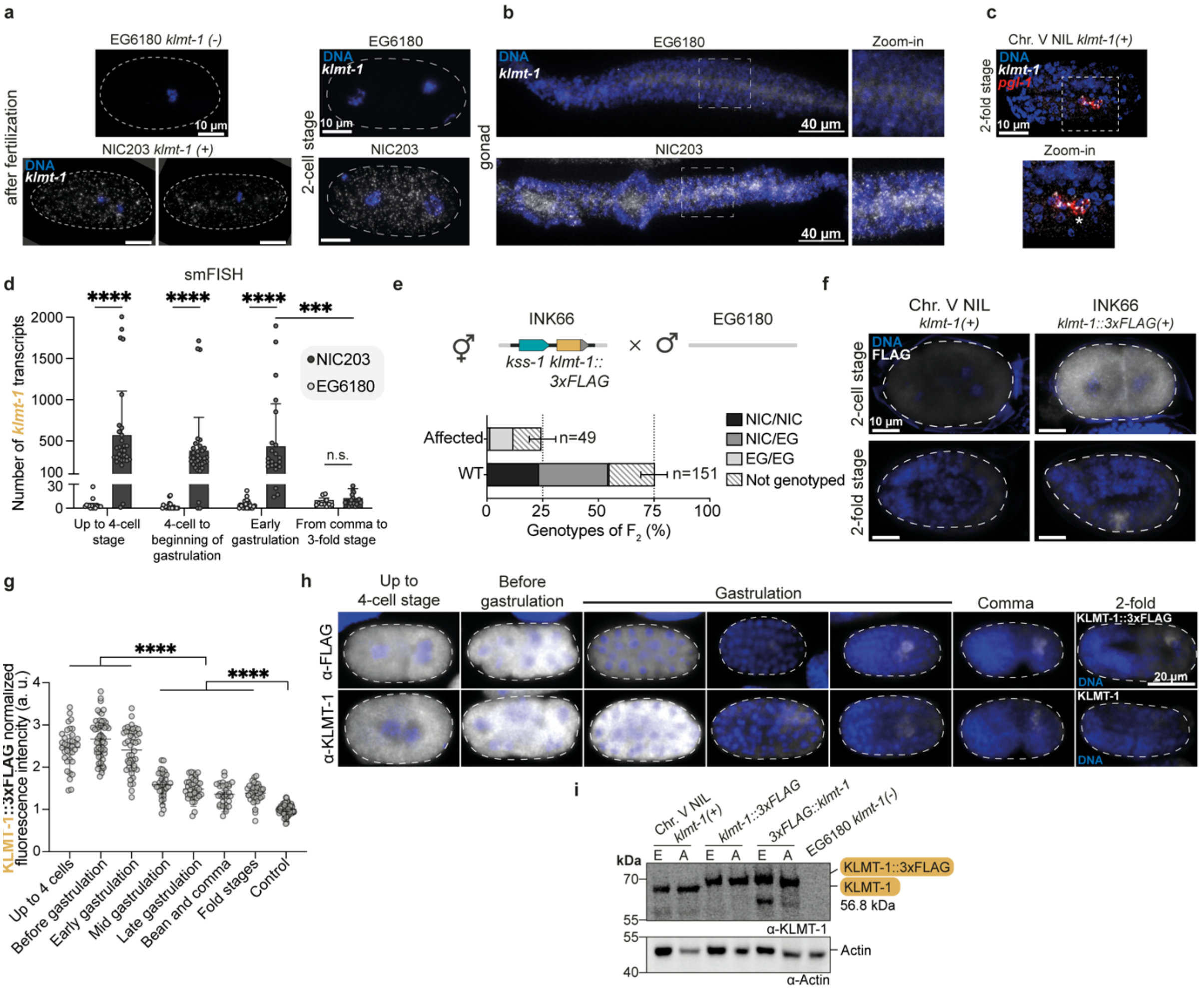

Supplementary Fig. 2. The *klmt-1* toxin is expressed in the adult gonad and during embryogenesis. **(a)** *klmt-1* expression prior to zygotic genome activation (prenuclear fusion stage) in NIC203 by smFISH. **(b)** *klmt-1* expression in the maternal gonad. EG6180 – negative control in (a) and (b). **(c)** *klmt-1* expression during late embryogenesis in Chr. V NIL; *pgl-1* is expressed in germ-line precursors. Chr. V NIL and NIC203 both carry a single copy of *klmt-1/kss-1* TA. **(d)** Quantification of *klmt-1* transcripts throughout embryonic development. NIC203 has significantly higher number of transcripts compared to EG6180 control in all stages before comma stage ($P_{adj} < 0.0001$ for up to 4-cell, up to beginning of gastrulation and early gastrulation stages; for comma stage: $P_{adj} > 0.9999$; NIC203 transcripts in early gastrulation versus comma stage: $P_{adj} = 0.0007$). Kruskal-Wallis test ($H(8) = 128.4$, $P < 0.0001$), followed by Dunn's post hoc test. 4-cell stage embryos were included in the "up to 4-cell stage" group. ≥11 embryos per group. **(e)** Genetic cross between the *klmt-1::3xflag* strain and EG6180 parental line. Sample sizes are shown for each phenotypic class. Error bars – mean with 95% CI, hybrid Wilson/Brown method. **(f)** Representative images of immunofluorescent staining of early and late embryo expressing KLMT-1::3xFLAG. Chr. V NIL – negative control. **(g)** KLMT-1::3xFLAG expression throughout embryogenesis. Embryos of early developmental stages have higher KLMT-1::3xFLAG expression compared to mid gastrulation and later developmental stages (for all pairwise comparisons between early and late stages $P_{adj} < 0.0001$). EG6180 mixed staged embryos – negative control. ≥30 embryos per group. Brown-Forsythe ANOVA test ($F^*(7, 222.6) = 167.9$, $P < 0.0001$), followed by Dunnett T3 post hoc test (for all stages versus control $P_{adj} < 0.0001$). Error bars – mean ± SD. **(h)** Representative embryos for KLMT-1::3xFLAG quantification. KLMT-1 monoclonal staining shows a similar pattern. **(i)** KLMT-1 monoclonal antibody recognizes KLMT-1, as well as 3xFLAG::KLMT-1 and KLMT-1::3xFLAG. Lysates were extracted from mixed embryos (E) or adult worms (A). EG6180 – negative control. Actin – loading control.

a

Mass-spectrometry of purified KLMT-1 protein sample

Description	Quantified peptides	MW (kDa)	Normalised area	Coverage (%)
KLMT-1 OS=*Caenohrabditis tropicalis*	79	58,94	5,05E+10	89,5
Protease 1 OS=*Achromobacter lyticus*	18	68,083	1,20E+10	20,5
30S ribosomal protein S15 OS=*E. coli*	7	10,263	5,37E+09	36,0
Trypsin OS=*Sus scrofa*	8	24,394	3,89E+09	43,3
Ferric uptake regulation protein OS=*E. coli*	17	16,784	1,32E+09	95,9
Regulator of sigma D OS=*E. coli*	8	18,231	7,94E+08	37,3
Elongation factor Tu 2 OS=*E. coli*	29	43,286	5,52E+08	72,8
50S ribosomal protein L28 OS=*E. coli*	10	9,001	2,47E+08	71,8
30S ribosomal protein S11 OS=*E. coli*	7	13,836	2,40E+08	49,6
50S ribosomal protein L13 OS=*E. coli*	11	16,009	1,97E+08	75,4

b

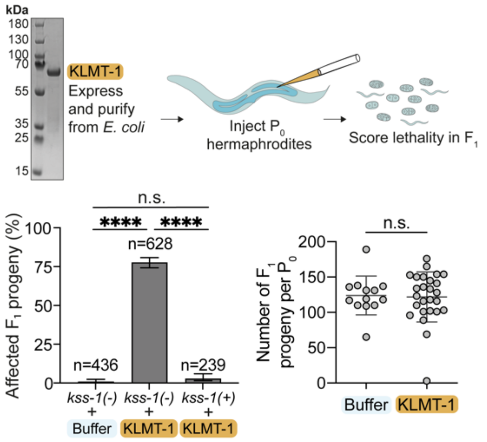

Supplementary Fig. 3. Maternal KLMT-1 is sufficient to poison embryos. (a) Confirmation that the purified protein is KLMT-1 by mass spectrometry. Ten proteins with the highest normalized area are listed in the table. **(b)** KLMT-1 protein purified from *E. coli* kills embryos when injected into the gonad of EG6180 hermaphrodites, but embryos expressing the KSS-1 antidote from a single copy transgene (INK563) are protected from KLMT-1 toxicity ($P < 0.0001$ for injection of KLMT-1 vs buffer, and for injection of KLMT-1 into EG6180 vs *kss-1(+)* strain; $P = 0.0597$ for buffer injection vs KLMT-1 injection into *kss-1(+)* strain, two-sided Fisher's exact test). At least 8 hermaphrodites were injected per condition. Error bars – mean with 95% CI, hybrid Wilson/Brown method (left). KLMT-1 does not affect the fecundity of injected mothers (two-sided unpaired t-test; $P = 0.8542$, n.s. – not significant). Error bars – mean ± SD.

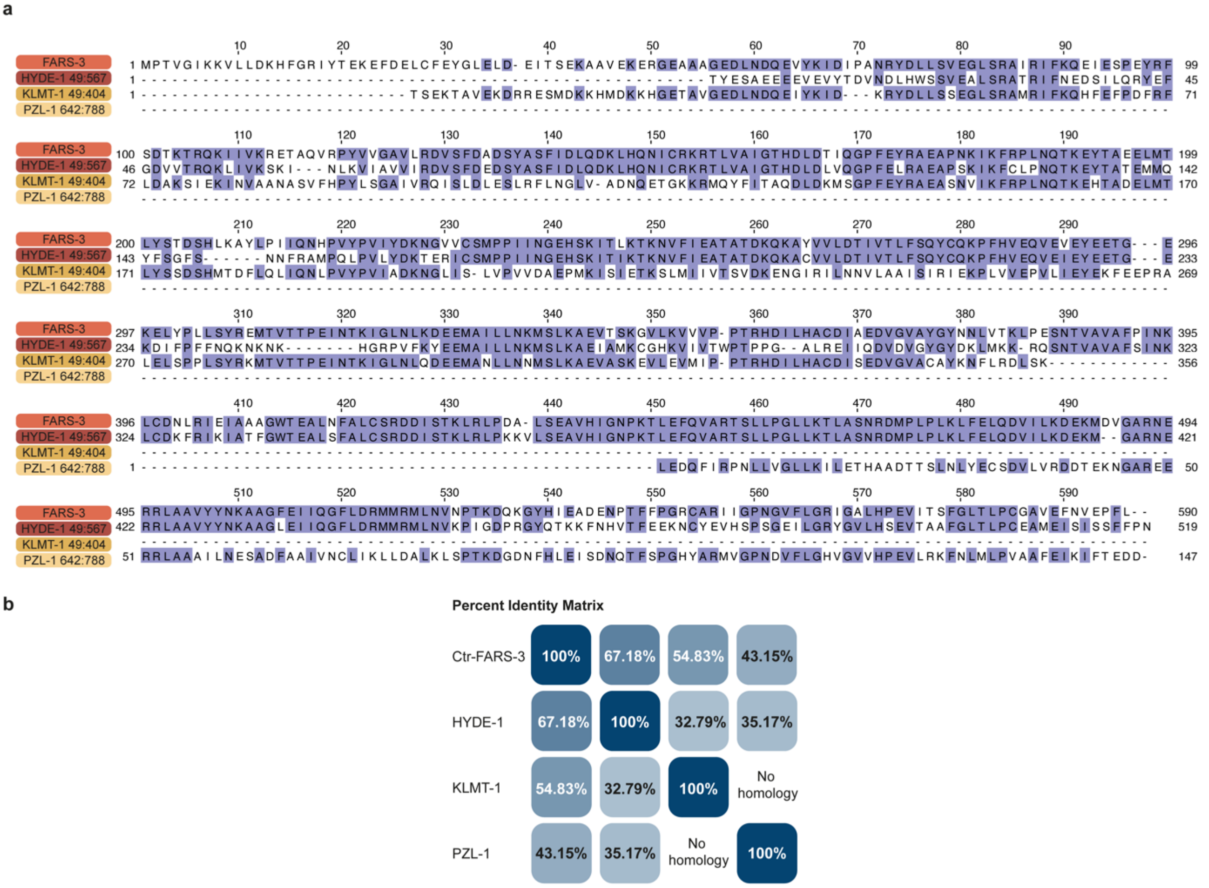

Supplementary Fig. 4. Pairwise alignments of FARS-3-derived toxins and *C. tropicalis* FARS-3. **(a)** CLUSTAL Omega multiple sequence alignment, colored according to BLOSUM 62 score, 50% of identity threshold. **(b)** Percent identity matrix for FARS-3-derived toxins. Only parts identified as derived from *fars-3* were used in this alignment (same sequences that were used for constructing the phylogenetic tree).

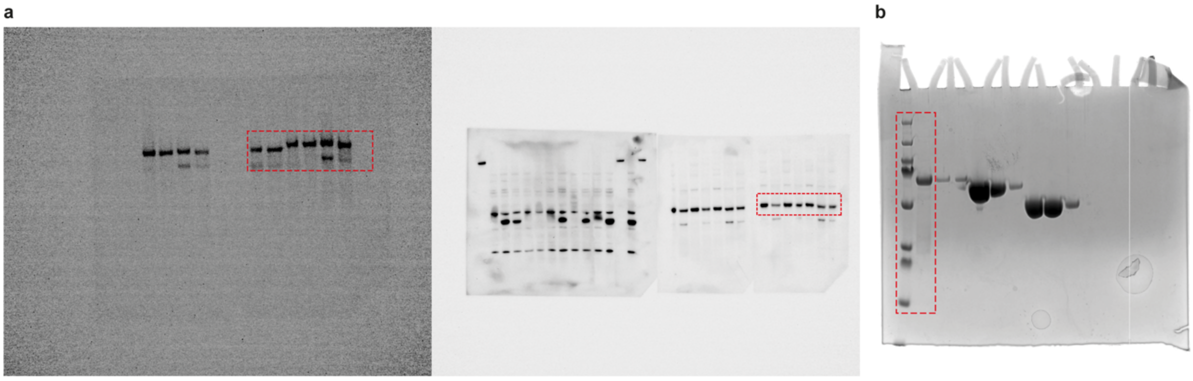

Supplementary Fig. 5. Unprocessed blots and gels used in Supplementary Figures. (a) Uncropped membrane used for Supplementary Fig. 2i. Membrane was stained with anti-FLAG antibody first (left), and then stripped and stained with anti-Actin antibody (right, right membrane). (b) Uncropped Coomassie stained gel used for Supplementary Fig. 3b. Regions shown in the Supplementary figures are marked with red dotted outline.

Supplementary Methods

Single molecule in situ hybridization.

Stellaris FISH probes targeting *klmt-1* and *pgl-1* were designed using the Stellaris RNA FISH Probe Designer (Biosearch Technologies). The probes were labeled with CAL Fluor Red 610 and Quasar 670, respectively (Biosearch Technologies). Embryos were processed according to adapted Raj et al. protocol[3], described in more detail by the probe manufacturer available at www.biosearchtech.com. Processed embryos were stained with DAPI (Merck, D9542, 6 ng/ml), mounted with Fluorshield (Sigma-Aldrich, F6182), and imaged at Axio Imager.Z2 (Zeiss) equipped with Hamamatsu Orca Flash 4 camera (pixel size: 6.5 µm) and HBO lamp. For imaging 63×/1.4 Plan-Apochromat Oil DIC objective was used, Z-stack images with 40 slices (step size 0.2 µm) were acquired. Filters used were: DAPI excitation 406/15 nm, emission 457/50 nm, CAL Fluor Red 610 excitation 545/30 nm, emission 610/75 nm, Quasar 670 excitation 620/60 nm, emission 700/70 nm. Quantification was performed using a MATLAB script from the Raj lab available at http://bitbucket.org/arjunrajlaboratory. Image of 2-fold stage embryo (Supplementary Fig. 2c) was deconvolved with Huygens Professional version 24.04.0p4 (Scientific Volume Imaging, The Netherlands, http://svi.nl).

KLMT-1 injection into gonads of hermaphrodites.

For each injection, a freshly prepared batch of KLMT-1 was used. Protein was concentrated and buffer was exchanged to injection buffer (50 mM Tris pH8 and NaCl 300 mM) using Vivaspin 500 Centrifugal Concentrator with 10 kDa cut-off. Final KLMT-1 concentration used for injection was 2.7 mg/ml. 0.75 µl (100 ng/µl) of co-injection marker pCFJ90-mSI (*myo-2p::mScarlet-I::unc-54 5'UTR*) was added to 30 µl protein injection mix to be able to visually distinguish the offspring that received an injection dose. After injection into both arms of the gonad hermaphrodites were kept on a recovery plate for 2 hours and replated on fresh plates. Next day, the worms were transferred to fresh plates again. The offspring from each plate was screened under stereomicroscope Axio Zoom.V16 (Zeiss), and the phenotypes were counted separately for the offspring with and without co-injection marker. Only offspring that received a co-injection marker were used to assess the proportion of affected offspring. Raw data is available in Supplementary Table 6.

Identification of *kss-2* coding variants

Aligned sequencing reads for 691 wild isolates of *Caenorhabditis tropicalis* were downloaded from CaeNDR. Files with aligned sequencing reads were converted to .fastq using SAMtools (version 1.9), and then aligned to NIC203 *kss-2* genomic region using BWA (version 0.7.17). Variants were called using the GATK HaplotypeCaller (version 4.0.1.2) with default settings. Variant effects were revealed

using SnpEff (version 4.3t), using a custom NIC203 *kss-2* database, and moderate-/high-effect homozygous variants were extracted for further analysis.

DNA and protein sequences of FARS-3, toxins, and antidotes identified in this study.

For genomic sequences exons are marked in uppercase. EG-PZL-1 and NIC-HYDE-1 do not act as toxins and EG-KSS-1 does not protect from KLMT-1 toxicity. Their protein sequences can be found at the end of the list.

```
>EG6180-fars-3
ATGCCAACCGTGGGAATCAAGAAAGTGCTTCTCGACAAACATTTCGGCCGAATTTATAgtaagttatcgatttttcgcggaaaataggttcgcc
agcaaaatctgcgattttgatgagaaaacttgggtttttcaaggttttaactattgaattcgagttctccgtattttaattcactttttacat
gtttatttcacgaaaaaatctatttttcaaataaaaattaatttttccaaaaaaaaattttttttgcaaaaaacctctgaaaaaagacaattt
cgataggaaaaactaggatttcctgtcaatttcaatcaagaaatgcatattttcagCGGAAAAGGAGTTCGACGAGCTCTGCTTCGAATATGGC
CTCGAGTTGGACGAAATTgtaggtttctcgagtcggcaaacccggttttcgcagatttcagtgcggaaatgcgtggaaaatcgctgatttcagc
gaattttgaaagaaaaccgctattattttcaagcggaattattatttactttattatttaatttttaattcttaatttttatttttaatggcga
aaaccggggattttccattgttttcgaccggaaaacctcttattttaggttttcagcgagaaaaacgctattttcaacgcaaaaatttagtag
ttttgaattctgaacagaagcgctatttttgggagaaaaacgaatttttgaatttaaaatagctatttttgacctaaaaatctattttaatag
ctgaaaaaaatgctttttttgacctaaaaccctattatgaccgctcaaaaatgctattttcggttatttctcacctaaaatcactaatttcgag
cgctgtacctaaacgcattctattagacaaaaaaaccgctattttcgactattttcaactcaaaaccacctttcaaacacattttaaacgaaaa
aacagctattttaacggccaaaacccccatttttccttcagACATCCGAAAAAGCGGCGGTCGAAAAGGAGCGCGGAGAAGCTGCCGCCGGAGA
GGATCTCAACGACCAGGAGGTCTACAAAATCGACATTCCCGCAAATCGATACGATTTGTTGAGTGTGGAGGGGCTCTCTAGAGCAATTCGAATC
TTCAAACAAGAGATCGAATCCCCAGAGTACCGATTCTCGGATACCAAGACCCGTCAGAAGATCATTGTGAAGCGCGAGACTGCGCAGGTCCGTC
CCTACGTCGTCGGCGCCGTTTTAAGAGACGTTAGCTTCGACGCGGATAGCTACGCGTCGTTCATCGATCTCCAGGATAAACTCCACCAGAATAT
CTGTCGGAAACGAACGCTCGTCGCCATCGGAACCCATGACTTGGATACAATTCAAGGGCCATTTGAGTATAGAGCAGAGGCTCCAAACAAAATT
AAATTCCGTCCGTTAAACCAGACGAAGGAGTACACGGCGGAAGAGCTAATGACGTTGTACTCGACTGACAGCCATCTGAAGGCCTACCTCCCCA
TCATCCAAAACCATCCGGTCTACCCGGTTATCTATGATAAAAATGGAGTGGTATGCTCAATGCCGCCTATCATCAACGGAGAGCACTCGAAGAT
CACTCTCAAGACCAAAAACGTGTTCATCGAGGCGACGGCGACGGATAAGCAGAAGGCGTACGTTGTCCTTGACACAATCGTCACCCTCTTCTCC
CAATACTGCCAAAAACCGTTCCATGTCGAACAGGTGGAGGTAGAATACGAGGAGACCGGAGAAAAGGAGCTCTATCCGCTTCTCTCCTATCGAG
AAATGACTGTGACGACGCCGGAGATCAACACGAAGATCGGATTGAACCTCAAAGATGAGGAGATGGCGATTCTACTAAACAAAATGTCCCTGAA
AGCGGAAGTCACATCGAAAGGAGTCCTCAAAGTGGTGGTTCCGCCAACTCGACACGATATTCTGCACGCATGTGACATCGCCGAAGATGTTGGA
GTGGCTTACGGGTACAATAACCTGGTCACAAAGCTTCCTGAGgtacgttttgaaaggagaactctattttcgagctcaaaatagggtttttga
gtcagaaaccattgtttttcagcttgaaatccgtctaaaaccccctttcaagcttaaaaaacctatgttctcagccttaaaagcacattttta
cttgaaaatgatgattttcgactcagaaacccctatttttggctctaaaaatcagttttgacgtggaaagtccagttttcattcagaaagcctt
tttaagctcagaaaaagatctattttaacttaaaaaccccatttccacccgaaaagccctcttttccaagagaaaaacccatttttttagtc
ttaggatcccctcttccaccctatggatcccttttccggagctgaaaaccctattttccaccttaaaacctttttttttctagcggaaatcccta
tttttccaccacaaaagccccttttccaagagaaaaatcccatttccacctctggggtcccctttttcctagtagaaatcctcgttttccactc
aaaaagccccttttctagtggaaatcactattttcgactcttgggatcccctcttccactctcagaatcctttctcctccccaaagtccctt
tttcctccccaaaatcctcctttcttccagTCGAACACCGTCGCCGTCGCCTTCCCAATCAACAAGCTCTGCGACAATCTCCGTATCGAAATCG
CTGCCGCCGGATGGACAGAAGCCCTCAACTTTGCTCTCTGCTCCCGAGACGACATCTCGACGAAGCTCCGCCTCCCCGATGCTCTCTCAGAAGC
TGTTCACATTGGAAATCCGAAGACACTGGAATTCCAGGTCGCTCGGACGTCTCTACTCCCTGGTCTTCTGAAGACCCTGGCCTCCAATCGGGAT
ATGCCTCTGCCACTGAAGCTCTTCGAACTCCAGGATGTCATTCTAAAGGATGAGAAGATGGATGTGGGAGCAAGAAACGAGAGACGGTTGGCAG
CTGTCTACTACAACAAAGCGGCCGGATTCGAGATTATCCAAGGATTTTTGGATCGAATGATGAGGATGCTGAATGTGAATCCGACGAAAGATCA
GAAAGGATATCATATTGAAGCGGATGAGAgtgagtttttatgtttttgattcgaaaggatccttggaattccgtcaattccatccaggatcccc
ctgaattcccctagatccttccctggatcctcttctagaacatggggatactataaggatcctatagatccttcaaatccctaaaaagatccct
atggatccctaaaactcccagaatcccctgaattccattgatctcacccccctggatccctcctagatcttcagaattccataaatttcttggga
tactccctagaatctcctgaacctccaggatccctagaatctccaggatccccgggatcctcctccctctcctctccttaatccattcatccat
tcttccagATCCGACGTTCTTCCCTGGCCGTTGTGCCCGAATCATTGGTCCAAATGGCGTCTTCCTCGGACGGATTGGAGCCCTCCATCCGGAG
GTCATCACCTCTTTCGGACTCACCCTTCCATGTGGAGCCGTGGAATTCAACGTGGAACCGTTCCTCTAA

>EG6180-FARS-3
MPTVGIKKVLLDKHFGRIYTEKEFDELCFEYGLELDEITSEKAAVEKERGEAAAGEDLNDQEVYKIDIPANRYDLLSVEGLSRAIRIFKQEIES
PEYRFSDTKTRQKIIVKRETAQVRPYVVGAVLRDVSFDADSYASFIDLQDKLHQNICRKRTLVAIGTHDLDTIQGPFEYRAEAPNKIKFRPLNQ
TKEYTAEELMTLYSTDSHLKAYLPIIQNHPVYPVIYDKNGVVCSMPPIINGEHSKITLKTKNVFIEATATDKQKAYVVLDTIVTLFSQYCQKPF
HVEQVEVEYEETGEKELYPLLSYREMTVTTPEINTKIGLNLKDEEMAILLNKMSLKAEVTSKGVLKVVVPPTRHDILHACDIAEDVGVAYGYNN
LVTKLPESNTVAVAFPINKLCDNLRIEIAAAGWTEALNFALCSRDDISTKLRLPDALSEAVHIGNPKTLEFQVARTSLLPGLLKTLASNRDMPL
PLKLFELQDVILKDEKMDVGARNERRLAAVYYNKAAGFEIIQGFLDRMMRMLNVNPTKDQKGYHIEADENPTFFPGRCARIIGPNGVFLGRIGA
LHPEVITSFGLTLPCGAVEFNVEPFL

>klmt-1
ATGTCCGAAAATCAGCGACTTTCGAACAACGGTTCTGTAGAAGAATTTTATACGgtaaaaatggtttttttaaaaagacaaagtgctttttga
gatagttctaagcggttttatgcgaaaacataatatttgcggcgattattaggttgaaaaaacaaattttccatggaaaaatgctattttcaag
aaaaaaaatttgtttcttcttttccagcgaataccaacagaaaagtagcaattttaatggcgaaaaccattattttctataatttttcacctga
aaatattaaattgtcccgtttgctaacccattttgcatttgaaaaacataaaatcccattcgaaatctcaaaaatatttattttcagGCTCCGG
ACGAATACGAAAAGACAACCACTGCATCTAAGCAGTTGCCGCCAAGTTTCAATGCTCCAGACCTTCCTCCAACTACTCCAGTGgtacgccatta
cgtttcttttatttttttttatattaaaaaataacaatttaaacgacttttcgccagaaaaaccacattttcgacataaaataactaaaaataat
tttttgacatgaaaatagtttttttaaataaaaaaatacgattttcagactaaaaaaatcatttccaatctgaaaattcctattttcccttgtcc
```

gaatctttttccatcttcaaaaacccatagtttgcaaaccacaattttccgagccgaaaactctacttcccgctgaaaaactgcattctcctca
ttaaaaaatttatttagctcaaaaaccctattttcgcccgagaaatcaccatttttctttttgaaaattctgtttcgacctaaaagacaccc
atttctttgaacattgaaatcctacttttccaaaactaaaaaccataattgcagcttgaaaatgatgaaaatgtggtttctccggacaaaaaa
ccatatcagtttctattttctatttttatggcaagaataacataattttctgttaatttttcgctgaataacccaatttttctttcagACATCC
GAAAAAACGGCGGTGGAAAAGGATCGCAGAGAATCTATGGACAAGAAGCATATGGACAAGAAGCACGGAGAGACGGCGGTCGGCGAGGATCTCA
ACGATCAGGAGATTTACAAAATCGACAAACGATACGATTTGTTGAGTTCAGAGGGGCTCTCCAGAGCTATGAGAATCTTCAAGCAACACTTCGA
ATTCCCGGACTTTCGTTTCCTAGATGCAAAGTCGATTGAGAAGATAAATGTGGCTGCGAATGCATCGGTCTTCCACCCATATTTATCCGGTGCG
ATTGTTCGCCAGATTTCATTAGATTTGGAATCTCTCAGATTTTTGAATGGATTGGTAGCTGATAACCAGGAGACGGGCAAGAAAAGAATGCAAT
ATTTCATCACAGCACAGGATTTGGACAAAATGAGTGGACCGTTTGAATATCGGGCCGAAGCTTCAAACGTAATCAAATTCCGTCCGTTAAATCA
GACGAAGGAGCACACGGCGGATGAGCTGATGACGTTGTACTCATCTGACAGCCATATGACAGATTTCCTTCAACTCATTCAAAATCTTCCTGTC
TACCCGGTAATCGCTGATAAGAATGGACTGATATCCTTGGTGCCAGTAGTCGATGCGGAACCTATGAAGATATCAAtcgagacgaagagtttga
tgattatcgtaacatcagttgataaagagaacggcatccgaatcctcaacaacgtcctcgccgctatttcgattcgaattgaaaaaccacttgt
cgttgagcccgtTTTGATTGAATACGAGAAATTCGAGGAACCGAGAGCCTTAGAGCTCTCCCCACCGCTCTCTTATCGCAAGATGACAGTGACG
ACGCCGGAGATCAACACGAAGATTGGATTGAATCTTCAAGATGAAGAGATGGCTAATCTACTAAATAATATGTCTCTGAAAGCGGAGGTCGCCT
CGAAAGAAGTCCTCGAAGTGATGATACCGCCCACTCGACACGATATTCTACACGCGTGTGACATTTCAGAAGACGTCGGAGTCGCGTGTGCCTA
CAAAAATTTCCTAAGGGATTTGTCAAAGgttcgttttcatttttccacatttaaaactagaaatttttcaaaacttgtttttgaaccaaaacc
cctgtttttaccacccaaaatcacctttctccgctccaagaactccttttcgacctcaaacacgtcacctttgatttgaagaatgaaaaaccaa
ctcctagaattttcatcttgagaaaatttggtttgaaaataaaatacggatttcagGAGCCACCAAACATCACAAATTTTAATCGGATCTCCTC
AACGCTTTCGAAGCTTCTGGAACTCGATGAAACTAATCCGGACAGTGTCTTCTCGAGAAAACACTCTTCCGAACTGAAGAAAGTGACTGCCAAA
GTGCTCACCCTAATGAAAGGAGAAGTCAAAAGAGCTGAGAAGAATTTGTTAATGATTCAGAGCAAAGTTGGATTGGAGATGAGgtattttcgca
cttcgcaacaaaacagcttcatttcgatttttcagTGACAGTGAGACGGAGAAAGCCGCCGACGAACAATCCGTGGAGAAAAAGAGAAACTC
CTCGTCACAGAATCCAATGGAAAGTTAA

>KLMT-1
MSENQRLSNNGSVEEFYTAPDEYEKTTTASKQLPPSFNAPDLPPTTPVTSEKTAVEKDRRESMDKKHMDKKHGETAVGEDLNDQEIYKIDKRYD
LLSSEGLSRAMRIFKQHFEFPDFRFLDAKSIEKINVAANASVFHPYLSGAIVRQISLDLESLRFLNGLVADNQETGKKRMQYFITAQDLDKMSG
PFEYRAEASNVIKFRPLNQTKEHTADELMTLYSSDSHMTDFLQLIQNLPVYPVIADKNGLISLVPVVDAEPMKISIETKSLMIIVTSVDKENGI
RILNNVLAAISIRIEKPLVVEPVLIEYEKFEEPRALELSPPLSYRKMTVTTPEINTKIGLNLQDEEMANLLNNMSLKAEVASKEVLEVMIPPTR
HDILHACDISEDVGVACAYKNFLRDLSKEPPNITNFNRISSTLSKLLELDETNPDSVFSRKHSSELKKVTAKVLTLMKGEVKRAEKNLLMIQSK
VGLEMSDSETEKAADEQSVEKKRNSSSQNPMES

>kss-1
ATGGAATTCCCCCTTATTCGACTCCCAGACGTTCCAAAAATTCAAATTATCCAATTGATGTCTATTGGGGAGCGgtaagtactccgaaagggtt
ttccaggacattcattaattttcagAATCAAACTGGCTCTTTCCTCTCGAAAAATGGAGAACTATCTGGCGTGGGTGTTCAAAAATCGCACTCT
AGATGCGGTTTATACTATTGAATTGCGAGGCGACACCTCTTTCATCGGTATCGACACAAAAACATGGGTACTGTGTATGAACCATTTCAAACCA
GAATGTAAAAAGCCGGGTTATATTTCAGTGGAAGAATTGAAACCGTGgttagttatctagagaaattcagtgaaagaatacggtgtttcagGAT
AGACGAGAAGTGGACAATGATGGAGAGAACTTTCAATGTGTTCATACGTCTTCAAAAGGTTCTTCCGTGTGTAATGACCAATTTATGCGTAACT
CTGAAGACCGAACCGACGCCGATTCTAAAAATCCTAAGTTATCCGTGCGTCGCGAACTTGACAGGTGTGCATTTCTATGGAGGAACAGTAGAAA
GAAACGAGTTGGATGCAATTATGGAATGGAGAAAGGAGAACACGATTCAGTATATTTCGGTCACGGATAACGAAATTCCATTGGATTATAGACA
TCCGAATgtgagttttcttttgaatagttccactttatcagaaaaaaatgtgcagaatattacatcaatgatttcagGCCTTTCGTTTCACTGG
AGTAGTGTATGGTGATGCTCGATGGATAAGTCTCGAAGATTTACTCTCGATGAGAAACTTCTATCGACCGATTTTCGGGAAAAATAGATTCACT
ACGAAGGATCTGAACACATTTATTAAATATTGGATCAACTGTGATGAGAATATGTTCACTTATCTAGTAATTGATTACAAAGATATCAATATAT
CTGAATTGATGAAGGATATTACTGTTCTCAAGGGTTTCCGATCCGAAAAACCATTTTATCTGATgttagttgcttcttttttttatttagagga
ttattagcaataattaattgatcaaaagaaaaaagaaatagttttcagAGCATCGAATTCAACTGAGAATCAAATTCTATTGACAATTCAATTG
ATAGAACACACTGAACCTCAAATACGAAATGTGCTCGACTTCTCGATTCAGTATGCTAATGAACCACATAAATTCAGGAGAGGCGGCACAGTGC
CTGCATGGACTCGAGAATACCGTATTCTTGGCCGATTGGCGAGAAAACGTTGGTTGGAAGAGGAGATGATTACTGTAGGAATATCGATTGAAGA
AATAAAAGAACTCGATGATTTGGAGGAGGAGTTGACTGTAGAGGAAGTTCGGATCATTGATGGAATAATGACTGTTCAAGAGAAACTGTAG

>KSS-1
MEFPLIRLPDVPKIQIIQLMSIGERIKLALSSRKMENYLAWVFKNRTLDAVYTIELRGDTSFIGIDTKTWVLCMNHFKPECKKPGYISVEELKP
WIDEKWTMMERTFNVFIRLQKVLPCVMTNLCVTLKTEPTPILKILSYPCVANLTGVHFYGGTVERNELDAIMEWRKENTIQYISVTDNEIPLDY
RHPNAFRFTGVVYGDARWISLEDLLSMRNFYRPIFGKNRFTTKDLNTFIKYWINCDENMFTYLVIDYKDINISELMKDITVLKGFRSEKPFYLI
ASNSTENQILLTIQLIEHTEPQIRNVLDFSIQYANEPHKFRRGGTVPAWTREYRILGRLARKRWLEEEMITVGISIEEIKELDDLEEELTVEEV
RIIDGIMTVQEKL

>pzl-1
ATGTGGTCATCGACAACTTCATCCCACCAAAATCAACAAAAAATTCATGAGAAAGTATATgtaagtgatcctttccaattccaatttcaaaaaa
aactttgatttttcacgttaaaatttttctgccctacaccaggccctaaacaattgaaaaactcgactatcaatccaatttattttgttagaa
acttatttctagcataataatatcctttctgataacaaacgaacaacatttttactgttttgaattaaaaactatctttcagccgcttatttg
aaaatccgcttttctcagttttgaaagtcaaaaactattgtttttctccagtttttttccagtttgcaaacggaataccacttttagctgtaa
aaacaatttttctaaaatctatcaaaatgttttcaaaaatttaccaaaacatatacaatttgcagAAAAACGAACATCAAGTCTGTCTTCCGC
CATGTGAGCTGAAAGACGAGGAATATGAGCCGAAAAAGTCGTTCTACGAAATGCATCAGCAAACGAAGCGGTGGACGAATTGGGAATCGCAACG
AATTGTAACTGCTCCGGGACACTGTGCAACAGTAGACAGTGTTCTTCTATTCGAAAAAAACGACAATAAATTCTGTCTCTCTGAAAGTCGTGAT
CGAGTGATTCGTTTTTGGGACGTCGATAACGTTGAACGAGGAGTTGATTCAGTGGCAAATCCATGGACAGTGGCACAAGATGATATGGCACAAT
TGGAATGGAGTTGGAATATGGCTCGTGATTCGAACACTGATAGATTCTACTCAACCTCATGGGATTCAACTGTTAAAAGCTGGGCGATCACCGA
CAACGGAGCCATTCAGAATCTGAATACAGTAAACGTCGGATCAGCAGTTCAAGCGGTTTCCTGTTCTGGAAATGAAAACGAAATCGTTTGTACT
ACTTTCGCAAAAAGAACAGCTGTAATTGATTCGAGAACTTTCGGAATCGTAGCTGAACACAGTCTTCATAAACGAGCGGTCATAGGATTAGCTG
TCAAGGAGAATAAGATATTCACGAGTGGAGAGGATCGATTGATGATGATGGTTGATCGAAGGAATATGAGTAGACCTGTTCTTTTTgtaagtga
acatctttataatgaactacagtagttttcagGAATACTGTCAAGACGCTTACAAATCCTCTCTATCTCTTCAACGGAATCAATTGCTCACTTC
AACTTCAGACGGAAAAGTCAAACTGTATGATGCGACGAATTTCCATGATTTTCAGACATATTCAgtgaggaatgaaacagttacagtaatccaa
tattctcttatcagGTCGGCGCCTTCACCCGTCAATCGCTTCTTCAACACGGAGCCCATTTCGTTATGGCTCGTTGTAAAAGAGGATATTATAA
ATTCCTTgtaagtgatcaactccctgaaaatgaacttgatccctcgagaaacacattttcacgatgcttcgaaagcatgtcacaagttctgatt
tttcagATGAACAGTCCTGGAATTCGGAGTCTAAAGAGATGTTCATCACATCAACTGACTGCTAAACCAGCTAAATTTGATTACTCgttagttt
gccatgaataccgtaacccaaacattcatttttcagATCCGAAATGAAGACACTTACAATTGGAAATTCGATGGATAGAAGTGAAAGACGATCC
ATGATTgtaagtaagaaactccctaataataaaaaaaacgaacttgatcccttgagaaacatggaacactttgttgaaaaagcaatcatttata
agaaaaatagcaatttcagaaaaataatatataacattttaaaagcattcgtcgctaatatcgctcatgctgtcgctttctccctctttttgt
tcacatcaatatcgtcctcatcacttcccgacctcaccacagaaaatccttcattaagatttccctctttgttcatttcgtgaaagttaaattc

ttcctctttggcgaagtttttctctttggcactctattgtccgtatttggtttcagagtgtgctcaaatagttttcgtgcgaccttcgcggga
aaacctctcaagaatcgaagagttctatctctaactttaagaaaaatttttgaaatggaaaactcgaaatttatgtacaatagtttgctaccc
gtatttccttctcttctcaaataattcttcgttttagaccacactaattcaatagttgcaaaacacagtggtactgcggcaagcaaagaatacg
aacacgacgcgtttggttaattcgtcgatctcgtagacagtgaacgccctgtttagcattttcagaaaaacgggacgtgtgaaagcagagtaa
taaagaacattatgatcatctagaaactatttaatatcagcgttgctcctgatagaattcaaaggctgaaataagcatgaagctataatagaag
aatctagattgattttcggcacttattcgatatgttttcctgttttatccgcttccgactcaaacagaggcaactcttctcatagaaagatca
taatttttcgcttttatgtcatggtggttattggttttctaatcttccacctttcaacccgacacaatgaattccgccgagtctttcggaattc
catccagtgtgacaacagcaatgacgattcctagcctcccttttttcttttccccttcttggacctgccatcaccgtttccatgtccaccaatct
tgatttttttataatttgtcaaatttttgcgttgctaaaatctttgtcattcctttatgtgcccatgtctcgtctaaatttccaaaatagggtga
acatttgtctcactcctctttctctcttccatacgcttgtctacgatatatcatagatttattgtcgattttcgatgttaaaggggtataccgc
taattctctcgttgtgcacgcgaaaaattgaataaatacgtgaatctttttttgaagaattaaagagggccagtaggaaaagtggatataggctc
cgataaagtgccagaaaaacactgaatggtagaaaaaaataatgcacccatgagtcagttgataatataggtcatcaggtaacatatatgtcat
ttttgtcttctaattagtacagaaactactgaaaaccttgtttatgtttttttagaacccgaaaattccagttgctctcactgacctacttattt
gaccaactagttagctatcattatcttcatcctttcctggctcccttttttcagctatttactctgatttcggctaaaacagtatgacctctatg
ttacatgatgacctatattgccagctgccttagaggtaacttcttttgtcctcctcgagattttataatgtttccagggcctttattcacttta
ctaatcactattcatgtctcttttattcaacttcaaaaaagaattcacggggttattcatttttcgcgtgcacaacgaggacattttatactgt
aaaatgcacaacgaaaccccttttttggcactttgagcgcttatatctcggttcagagacgtttatggaaaaagtgaagtatttactagagttc
gatcacgctccgcgagctgaatcgaatcatataagacttaagaaaatttgagctaattttgaattagcgtattacccctttaatatgtcagaaa
tagaagggttttaatagttcacgattacatgacaagataaagttaggaatggaaatcaggctattttctttcttcttcctcctgtcggtatccc
gtttgcctcttccatctttaaactggttcaattataataattcgatcctcatcttctgcttataccatatttttttcttggtatcctccggtagt
gtttcttccctttttttgaccctcatctttctccggggtcattctgctccatctccttgctgatttattttcgttcatgatatccccaatagttg
tttgatctttttccatgtctgcttcgtcatagtcacgacactccttgcggccacaaacgattaagctgtatggtaataaggggggataaggtaat
tgagactaatatgatacgctgttggctgagtatggtatgttgttagctaaaggtgatggcgtgggtacatgggaatatgctgttgctgcgtata
ctgtgtttggtggcattgtgggcaattgtggctcatctttgaactgaaggagtgttattcttttttcttttcctttctcgatctctgttatttc
taatgatattaattgttcttggtttgataatatttcggttgttgttttaagttctactgtgctgatttcctcatctttgccatgtttcgctcat
gtattttatcgataatcccgttttctaggtgtgcctttccgtagccctaccatttgaaaagctttttccatgtctcgcttattatctatgtttt
tgtgctaattcttaatagtatttctagcggttctggtattagtggtgggccttgatatactgcttggcttagactaatttctcctctgactgca
ttgctgtcctatgttgttactgtcactatgcatgtgaatcttttgactgtttatgcatagattaaagtttcctgtcgcgaagatgacgtgccgg
aaaattcatgaatcagatagagctactctactctgtttattgtgtttttcatgcatgtcctctactgattatcagatatttcccattccatcac
ccaggattatcagttggcagtacattgtcaatgatcatgatccactttaagttggctgatattagaaatgaacaaagaaaatttggttttaatt
ttttttcaaagtgctgcaaacggttagactcaacctaatattctcgctcatttttctccgttggggcttctttgatgttatttggtctttccc
tgtatctgagattactaaggttttggccatgttgtcctggctctgtttcctattgttcttatggtttctcttgttccctacagttatctcattg
gttacattaatcttgtgtttcctgtagcgaccaagttgattatactgtaagtgtgttttctaccagcattgtatcctcttttgttattagttta
tcgtcgctctccgtaggagaattatcggtatttgttttgaggtataaacctcgtgaggggctatgagcggttagagctcaactcgattttttttt
gttttcaatgtgtaaccttaccaacagcgacgcactaaatcatcatgcttgtaagacaagtggagattattggttcaaccttctgtgttagcat
cttggaggttaattatttccaccgaagaaagctatgcctacccccttatcaaaaacatggttttaaacgttccctctttgcaagctaggactc
ataacttgactaaaaccggatttatctttataaaattcacacactggtcagaaactactcaagtaatgaaatttgagaattgaagacttcaaaa
agaactaaaactttgtttaaatgcacctaaactgaagtaaacttgattacctgaagttctgcgccaccttgtaccactcgctctgcttctatct
tttacaatctctcactggggggttgtttcttcagcagttgtaacacagctcattttcttagaagaaaaacttactctcatatagccggctaccgt
cctgcatttcttggattggtggttcctttttttacgaaataaggaagctctggtgcgtcttgggcggtgaatcctgataaatgtttgatgagtgg
gatctgtttcgttcttgagtgtaatgccctctcacatgcagtctcgtcttttcgttcaaaggtgtgttctctgtctgtctctctctctctgtct
ctctctctctctctctctctctgttttgctttcttggttgttattactgaaaaacacttgatgatctgtctttttatagattcctgtcatgtct
ttctctatgcgttctctctctatccctgtttttctgctctcttttctggttatccgtcaaattctggtatcagtgatgttttattaaaaattat
cgctcttcccactttcttttgatcatatgtcccaattcttctagtttctgatttgcctctgctagtctgccttgtgtgtcttcagtgttttcta
tagttgcgagtacgtcatctgttactcttcctcctaattcttcacacgctgtttaggtttttttgttcgtggtttttctcggatttctttagatt
acctaggtttctctcttagctaccgtaattaaatttgattttccccgatcagtcactggtttctctagtttactctagttaccctttgctttg
tttttgtgattacttttttttatttcttaaattaaactaacatagtatattctctttggtcgatgcaccactttgacacttcgcgagcggttga
atattttatcaatttgttctcgatggtattaggtcggtacataagtcctgacggatttgctggtcgaacttgttgaatgtatttcactgttca
aacttaagagagacggctaagtttctgtcccgtgaaaattcaaagcttcctcttctctttcatttttagaaatttcatcatatcattttatttg
tactcctatatggcgtggtattcattgaaacgagttttagtttagtggttattaacaacggacaaacactattttaaaacagttagcgatgcca
gtttcaatcgactagaaatagggagttatccatggatttccatccccctgcaaaaagaatgatgtcttcaaagttgagtcaattgtaaaatgag
atcgaaagagattcatttcaaattttgtgttaaggatttcgggtatctggtgtatgcatttgagtaaaaaaccaaaaaaaaaggattgaaaaaat
tcaccagagacaaaaactcaaaagatgtgactcacccttttttgtgactccacacaattccgaaacatgggagaccaccaggttgaatctttttct
tttcttccgatacaggaatcagtggttcaaataatttttggtggtaataaacgagtaatgaatggttgttatgagcaaggttcttagtttgccg
cttcaccctattagccgtttgatgtcattattcatgaaattcatgatgtggtcagtgtgacgcttttccagaaaagtggtgtactaggagaaca
aagatgatagtatcacaatcatgcccatagtctatatttctcagaacagagacaaatgctcctgtattcatctccattcttgagaaatgtctaa
attttatttggagtttgttggtattcagcatactctcgaagaaaggatcgacataaaactcactggatgtgatttgacaatgactttaaattta
tcatgcagatcattgttagaagagttgattaacaagtttgcaattaccagtcgaaagttcaagatggaaaaaattcaactcagaccgtatatta
aaaaagtagtgacttcgtatttcgagtcatctctataaaagttctaaaggcagagacaaatgtatgatggggggtgcattaagagacacttgtgg
atgaacatggacaatatttgcaatttagctattagctctgtggcaagttacacgtgaaccttgtcgacccaaacatccgtcaggacttatgggc
caacctaataatatattaataatataggctaggttcaaatatttctcattttggagtgaaagaaataagaaacagaatgagtagatggttcagc
aaaccttgaattttcgtgattaatacagaaggagccagctttgtggtgtcaggctgaaaaaaagtaaagtcagcttacttacttcactcaaaaa
tatgaaattttgaaattagtcccttattatatgtcagaaatagaacggttttaatagttcacgattacatgacaagataaagtttggaatggga
atcaggctattttctttcttcttcctcctgtcggtattctgattgctcctcccatctttagactggttcaattaatttgatcctcatcttctgc
tcataccatatttttttcttgatatcctccggaagcgtttcttcctttttttgaccctcctctttctccgtggtcattctgctatctctaagctt
catctccctgctaatttatttccgtcatgatatcctccgatgttcctctttctctgatcctttttcttatctgctccgccgtagtcaagacagg
aattacccggagaatcatttttttttccactaaaaaccttctgaattcatccattttcaacttaaaaagacattttttcgaatgagtcattcagt
ttttaaaaagcgtcaaatccttgagaacctacaataatttcatttactgttcccgagagtctttttttttgtggaaaaacgtcattttaatccca
ttcttttcctaagaattgtcgaaaactactcttaagtctcgaaatttacaatttccatttgatttccctctcaaaaaatagttttcaaatttaa
aaatgcagtttctgacaaaaacttgcttctttcgaaatttcaatttaacaaaccaatttttatattatttttcttctgaaaactctcttaaaac
atataatttcacaacattcatgctctttttctttctcaaaatcggatgaatcccataaagaaccgtcaaatactctcttaaccctctaatttca
cggtcgttgatcgtttgataactgtaaacctctccaagtcctattttcaaccaacaatctcatttttttcagTCAGAAGATCTCGATGAGAAATT
CGAAGTCGAAGTGGATACATGTCCACATTTGCCCACAAACTTTGAAACAATGAAATGGCAGGAGCGGCGAAGATCCTTGGAATCCTTCCTCCAA
ACTTTAACTGGAAAGAAATGTATCTTGTCTAACATTTCTTACGATGCATCGATTAAAAAACTGCAAAAGATAATTCGGAACGACGCCAACATAT
TTTGTCAAGTGTTGGCAATCAGAAGCGTTACATGGGTTGCCACGGAACTCGGCGCGGAATTCTCCAAATTCTCTGTTTCTCTTCTACCAGATCT
GTTGGAAAAGATGAAAGAGAAAAAACAGATATTGAGAAAGCCCCTTATTCGATGTACTTTGGAGGTTGGAAAGACGTTGCCGTTGGAAGATGGA
ATTCCAGTGATTTTATCGGCTCTCTCCATGGCAAATCCGGAGATAAAGAAGCAAACGATGTTATTTGTGGTTCAACAGCTTGAGGCGATGCAAT

TGGAGAGATTAAAAGGATTCATTCCATCATTACTTCCAGTTCTTGTTGAGCTTACAAAAAATGCTACTCAAGATGTCCGTGAAGTCGCTGTCCA
CGCCCTCGAATCAATTTACTGGAAGATAGGCCGATGTCGAATGCAATCTTTGTTATCAAAGTGTAGACATCTATTACCCAAAAGACACCCTTGG
CATTGGATTAAAAAAGGATCTGACGAAAAAAAGAAAACAGAAAAACCGCCTTCTTCTTTTTTTCATGAAGCTATTGGCTCCATCATTTACTCCG
GAGAAGAACTTCATGCCGCCAGATTCAGCAGATCCAACTGTATCCGGGTCGATCTGGAGGATCAGTTTATCCGACCGAATCTTCTTGTGGGACT
TTTGAAGATCCTGGAAACTCATGCAGCCGACACTACATCTTTGAATCTGTACGAGTGCAGTGATGTTTTGGTGAGAGATGATACGGAGAAAAAC
GGAGCAAGAGAAGAGCGACGTTTGGCTGCTGCCATTCTCAATGAATCGGCCGATTTCGCTGCAATAGTGAACTGCCTCATCAAGCTGTTGGATG
CGTTAAAACTGAGTCCGACGAAAGACGGCGATAATTTTCATTTGGAAATATCGGATAgtgagttcttgttcgaaacctcaaacccttggaatg
atcattgaatttggggatttttacctagtttccattcgagttttctttatactccctaaaaacatctgattccatgaatgctctcatgaatatt
atgaatcctcagaatatcaatgaagatcttttagaagcctagaatctcattcctctagaatctttttccatcttaaattccaagaacccttta
atgaattctcttagatgctactttgaattcctctagaatccctgaaattccatgaacctccatcctgaatcttctgaatctcctttctctctt
tcagATCAAACCTTCTCGCCTGGACACTACGCGCGAATGGTTGGCCCGAACGACGTATTTCTCGGCCATGTTGGAGTCGTTCATCCGGAGGTAC
TCAGGAAATTCAACCTCATGTTGCCGGTTGCCGCCTTCGAGATCAAAATCTTTACGGAGGACGACTAA

>PZL-1
MWSSTTSSHQNQQKIHEKVYKNEHQVCLPPCELKDEEYEPKKSFYEMHQQTKRWTNWESQRIVTAPGHCATVDSVLLFEKNDNKFCLSESRDRV
IRFWDVDNVERGVDSVANPWTVAQDDMAQLEWSWNMARDSNTDRFYSTSWDSTVKSWAITDNGAIQNLNTVNVGSAVQAVSCSGNENEIVCTTF
AKRTAVIDSRTFGIVAEHSLHKRAVIGLAVKENKIFTSGEDRLMMMVDRRNMSRPVLFEYCQDAYKSSLSLQRNQLLTSTSDGKVKLYDATNFH
DFQTYSVGAFTRQSLLQHGAHFVMARCKRGYYKFLMNSPGIRSLKRCSSHQLTAKPAKFDYSSEMKTLTIGNSMDRSERRSMISEDLDEKFEVE
VDTCPHLPTNFETMKWQERRRSLESFLQTLTGKKCILSNISYDASIKKLQKIIRNDANIFCQVLAIRSVTWVATELGAEFSKFSVSLLPDLLEK
MKEKKQILRKPLIRCTLEVGKTLPLEDGIPVILSALSMANPEIKKQTMLFVVQQLEAMQLERLKGFIPSLLPVLVELTKNATQDVREVAVHALE
SIYWKIGRCRMQSLLSKCRHLLPKRHPWHWIKKGSDEKKKTEKPPSSFFHEAIGSIIYSGEELHAARFSRSNCIRVDLEDQFIRPNLLVGLLKI
LETHAADTTSLNLYECSDVLVRDDTEKNGAREERRLAAAILNESADFAAIVNCLIKLLDALKLSPTKDGDNFHLEISDNQTFSPGHYARMVGPN
DVFLGHVGVVHPEVLRKFNLMLPVAAFEIKIFTEDD

>kss-2
ATGGAATTCTCTCTTATTCGACTGCCAGATGTTCCCAAAATTCAAATTATTCAATTGATGACGATTGGCGAGAGgtgagtatctcgaggttttc
tttttgttatatttgttttacagAATTAAACTGGTCATCTCTTCTCGAAGAATGGAGAACTATTTGTCCAGGGTATTCAAGAAGCCCAATACA
AATTCCGATTATAATATGAACTTGAAAGGCAAATTTTCTTTCATTTCTATCGGAGACGACGAGTGGAGAGTGTTTCTGGGTCCTTCGAAGATGG
AGTATAGTAAGCCGGATAATATCTCAGAGGAGGATGTGAAACCATGgtgagttcccagaaatctcgagtaatgtactcaaagaaaacggagatt
cagGATAAACGAAAAGTGTACAGTGATCGAAAATACTCTTAACGTGTTCGTACGTCTCCAAAACGTGTTCCCTTGTGGGACTACCAACTTGTGC
GTAGATTTGAACAAAATCGATCCGATGCCGATTCCGAATATACTGAGTTATCCCTGTGTCGCAAATTTGACAGGTATTCATTTCTACGGAGGAA
CAGTACAGAAATACGAGTTGGATGCAATTATGGAGTGGAGAAAAGAGAATACGATTCAGTTCATTACAATTTCAGATAACAAAATTCCATTGGA
CTATAGACACCCAAACgtgagtcaactttcaaacatttatatcttaattgccagtggagacattgggaaatggtcaacttcgaagttgctcatc
gtaaaatttagttgatcaaaataaacagaacgacgctgttatttttaatcacgttgtttcacctcttctgtaagagctggcaatatcggaaccc
ttaggacacgcccactattatgccaatctcgaaaaatggcagttctggccgaagatccgacaagttcttccacgtgaggtcgggaagcgaaaat
aaaattggtacagtaagaagttcaaaccataacccatgcgcctttaaggttttcaatagagagcgttaaaggcgcacgacaaaaagtaaaaagt
tcttactgtaccaattttattttcgctccccgacctcacgtggaagaactgtcggatcttcggccagaactgccatttttcgagattggcataa
tattggagaggaggaggaaggagaggggagaattgtggtagttgaggctgcgcggagaagagggagatggtgacccttctatactcctttatat
tttatacttcttatgataataaataatgatagagtggaaccaagacttagtttgaactcaagtatcaagggtctgaagtataataggtactcgc
ctattacacttccaatatttacaaaaaacaactcggcatcgataaagcgttgaccactttctgatatctacactgcagaccaagatgcatcgat
attcagGCTCTTCGCTTCTCCGGGGTGATGTACAACGACGCTCGATGGGTACGAATCGAAGACTTACTATCAATGAGAAACGTGATCCGATCGT
TTTTCGTAGACAATAATTTCAGTATGCAAGATCTGAACACATTTATCAAATATTGGACCAACTGCGACGAAAATATGTTTACATATCTCTATAT
TGGATACAAAAGTTTAGATATGTTAGAATTGATGAAGGATATCACTGTTCTCAAGGGTTTCCGATCCAAAAAATCATTTTATCTCATgttagtc
cttttattttataacgagaaacctcgagatttcaatgataaataagccataaattccaaaaaaaaaatttccagAGCATCGAATACAACTGAG
AACCAACTTTTGATGACGGTTAGATTGGTATCTTATCCTGCCGAACCCGGACGACGAGAGCTCGAGTTCTACGTTCTGTCTGCCAACACACCTC
ATCATGTACAGGGTGGCAGCACGGAACCCGCGTGGACTCGAGAGTATCGCATTTTGCGACGATTGGCGAGAAAACGTTGGCTGGATGAGAAGAG
GGTTACGGTAGAAGAGGCAAAGGAACTGGATGATTTGGAGAAGGAGTTGGCTGCTGAGGGAGTTCGGATGATTGATGGAATAATGACTGTAGAA
CAGAAACTGTAA

>KSS-2
MEFSLIRLPDVPKIQIIQLMTIGERIKLVISSRRMENYLSRVFKKPNTNSDYNMNLKGKFSFISIGDDEWRVFLGPSKMEYSKPDNISEEDVKP
WINEKCTVIENTLNVFVRLQNVFPCGTTNLCVDLNKIDPMPIPNILSYPCVANLTGIHFYGGTVQKYELDAIMEWRKENTIQFITISDNKIPLD
YRHPNALRFSGVMYNDARWVRIEDLLSMRNVIRSFFVDNNFSMQDLNTFIKYWTNCDENMFTYLYIGYKSLDMLELMKDITVLKGFRSKKSFYL
IASNTTENQLLMTVRLVSYPAEPGRRELEFYVLSANTPHHVQGGSTEPAWTREYRILRRLARKRWLDEKRVTVEEAKELDDLEKELAAEGVRMI
DGIMTVEQKL

>EG-kss-2
ATGGAATTCTCTCTTATTCGACTGCCAGATGTTCCCAAAATTCAAATTATTCAATTGATGACGATTGGCGAGAGgtgagtatctcgaggttttc
tttttgttatatttgttttacagAATTAAACTGGTCATCTCTTCTCGAAGAATGGAGAACTATTTGTCCAGGGTATTCAAGAAGCCCAATACA
AATTCCGATTATAATATGAACTTGAAAGGCAAATTTTCTTTCATTTCTATCGGAGACGACGAGTGGAGAGTGTTTATGGGTCCTTCGAAGATGG
AGTATAGTAAGCCGGATAATATCTCAGAGGAGGATGTGAAACCATGgtgagttcccagaaatctcgagtaatgtactcaaagaaaacggagatt
cagGATAAACGAAAAGTGTACAGTGATCGAAAATACTCTTAACGTGTTCGTACGTCTCCAAAACGTGTTCCCTTGTGGGACTACCAACTTGTGC
GTAGATTTGAACAAAATCGATCCGATGCCGATTCCGAATATACTGAGTTATCCCTGTGTCGCAAATTTGACAGGTATTCATTTCTACGGAGGAA
CAGTACAGAAATACGAGTTGGATGCAATTATGGAGTGGAGAAAAGAGAATACGATTCAGTTCATTACAATTTCAGATAACAAAATTCCATTGGA
CTATAGACACCCAAACgtgagtcaactttcaaacatttatatcttaattgccagtggagacattgggaaatggtcaacttcgaagttgctcatc
gtaaaatttagttgatcaaaataaacagaacgacgctgttatttttaatcacgttgtttcacctcttctgtaagagctggcaatatcggaaccc
ttaggacacgcccactattatgccaatctcgaaaaatggcagttctggccgaagatccgacaagttcttccacgtgaggtcgggaagcgaaaat
aaaattggtacagtaagaagttcaaaccataacccatgcgcctttaaggttttcaatagagagcgttaaaggcgcacgacaaaaagtaaaaagt
tcttactgtaccaattttattttcgctccccgacctcacgtggaagaactgtcggatcttcggccagaactgccatttttcgagattggcataa
tattggagaggaggaggaaggagaggggagaattgtggtagttgaggctgcgcggagaagagggagatggtgacccttctatactcctttatat
tttatacttcttatgataataaataatgatagagtggaaccaagacttagtttgaactcaagtattaagggtctgaagtataataggtactcgc
ctattacacttccaatatttacaaaaaacaactcggcatcgataaagcgttgaccactttctgatatctacactgcagaccaagatgcatcgat
attcagGCTCTTCGCTTCTCCGGGGTGATGTACAACGACGCTCGATGGGTACGAATCGAAGACTTACTATCAATGAGAAACGTGATCCGATCGT
TTTTCGTAGACAATAATTTCAGTATGCAAGATCTGAACACATTTATCAAATATTGGACCAACTGCGACGAAAATATGTTTACATATCTCTATAT
TGGATACAAAAGTTTAGATATGTTAGAATTGATGAAGGATATCACTGTTCTCAAGGGTTTCCGATCCAAAAAATCATTTTATCTCATgttagtc
cttttattttataacgagaaacctcgagatttcaatgataaataagccataaattccaaaaaaaaaatttccagAGCATCGAATACAACTGAG
AACCAACTTTTGATGACGGTTGGATTGGTATCTTATCCTGCCGAGCCAGGACGACGAGAGCTCGAGTTCTACGTTCTGTCTGCCAACACACCTC

ATCATGTACAGGGTGGCAGCACGGAACCCGCGTGGACTCGAGAGTATCGCATTTTGCGACGATTGGCGAGAAAACGTTGGCTGGATGAGAAGAG
GGTTACGGTAGAAGAGGCAAAGGAACTGGATGATTTGGAGAAGGAGTTGGCTGCTGAGGGAGTTCGGATGATTGATGGAATAATGACTGTAGAA
CAGAAACTGTAA

>EG-KSS-2
MEFSLIRLPDVPKIQIIQLMTIGERIKLVISSRRMENYLSRVFKKPNTNSDYNMNLKGKFSFISIGDDEWRVFMGPSKMEYSKPDNISEEDVKP
WINEKCTVIENTLNVFVRLQNVFPCGTTNLCVDLNKIDPMPIPNILSYPCVANLTGIHFYGGTVQKYELDAIMEWRKENTIQFITISDNKIPLD
YRHPNALRFSGVMYNDARWVRIEDLLSMRNVIRSFFVDNNFSMQDLNTFIKYWTNCDENMFTYLYIGYKSLDMLELMKDITVLKGFRSKKSFYL
IASNTTENQLLMTVGLVSYPAEPGRRELEFYVLSANTPHHVQGGSTEPAWTREYRILRRLARKRWLDEKRVTVEEAKELDDLEKELAAEGVRMI
DGIMTVEQKL

>hyde-1
ATGAGTAACGCTCCCAGCTCGACAGACGCACCGTTAGCGgtatgttcatattaagctggtttttagtgaaaactggccacttttcacacatttt
aataaagaaaaaaaaaacagtttttcacataaaatgctattttcaaataaaaataacatttctcagtcgaaatacgctgtttgcttctgaaaagt
tgttctaatgaaaaggttactaatttgaattcaataatttttttccagcgaattttaacagaaaaaagctattttgctacaaaaagcgtgatttc
ctacaattttcaccttaaaacccaaactgcgcactttttttaagctagtttgcatttataaaacgcctaatcctataataaacaataatttttt
gcagGAGAACGACTACGACGACCTTTTTGCCTACGGTCAGCGACCGCCAACACCTCAACTTCCACGCCCAgtacgtttatttctttttttaaccg
aataatattttcaattatgattttcagGCTACCCCAATCGACTCTGGCCCCGAGTTGGACGAGATTgtaggtttttttaatccggccgacgctgt
tttcgctgttttcactgcagaaaacgcgttccgtgcctgagaaatcgctgtttctagcgaatttcgaaagaaacccgctattttctagcggaat
tatcatttatttttattatttaaattttttaatttaaattttttcatgacggaaaccataattttcattcttttcgaccgtaaaacctc
tttttttcgaggggtttaactggaaaaacgctatttttaactcaaaaaattttttgtatcttcgaattccgaacagaaacgtcgtttttgggggga
aaaacgaatttttcaaatttaaaatagctatttttttacctaaagtcctggttttacagctgacaatgctattttcgattattttcgatctaaaa
ccctctttttacgggtaaaaacgcttctttcggttattttttcatctaaaaaccctcatttcaagcttggtaccggaaaacgtcatttttagac
aaaaaacggttatttttactgttttgaacctagaaccctcttaaaacactcaaaaaaaatctattttttgactattttcgaccttgacccgtctt
tttacagctgaaaagtgctactttcgggtgtttttttacctaaaaacccttcagttcaagcgatgtaccggaaaagttcatttttagaaaaaaaa
cgttatttttactgttttgaacctaaaaccctcttaaaacactcaaaaaaaaacctatttttgactattttcgaccttgaaccgtctttttacag
ctgaaaagtgctactttcgggtgtttttcacctaaaaaccccgtagttcaagcgatgtaccgaaaaacttcatttttagagaaaaaccgttatt
tttactgttttgaacctaaaaccctcttaaaacactcaaaaaaaaacctatttttgactattttcgaccttgacccgtctttttacagctgaaa
agtgctactttcgggtgtttttttacctaaaaacccttcagttcaagcgatgtaccggaaaacttcatttttagaaaaaaaaacgttatttttact
gttttgaacctaaaaccctcttaaaacactcaaaaaaaaacctatttttgactattttcgaccttgaaccgtctttttacagctgaaaagtgcta
ctttcgggtgtttttcacctaaaaaccccgtagttcaagcgatgtaccgaaaaacttcatttttagagaaaaaccgttatttttactattttga
acctaaaaccctctttaaaacacatctgaaacaaaaatatctgttttgacagctaaaaaccctatattttccttcagACATATGAATCAGCAGA
GGAAGAAGAGGTGGAGGTTTATACGGATGTAAACGATCTTCATTGGTCAAGCGTTGAGGCGCTCTCCAGAGCTACCCGCATCTTCAACGAAGAC
AGTATATTGCAACGATACGAGTTCGGAGACGTAGTGACCCGACAGAAGCTGATTGTAAAGTCAAAGATCAATCTCAAAGTTATCGCCGTTGTTA
TTCGCGATGTTAGCTTCGACGAGGATAGCTACGCGTCGTTCATCGATCTCCAGGACAAACTCCATCAGAATATCTGTCGGAAACGAACGCTGGT
CGCCATCGGAACCCATGACTTGGATTTAGTTCAAGGTCCGTTCGAATTGCGGGCAGAAGCCCCAAGTAAGATTAAATTCTGTCTTCCGAATCAG
ACGAAGGAGTACACGGCGACTGAGATGATGCAGTACTTCAGCGGCTTCTCTAATAATTTCCGAGCCATGCCACAGCTCCCTGTTTTATATGATA
AAACAGAACGGATCTGCTCCATGCCGCCTATTATCAATGGAGAACACTCGAAGATCACTATCAAGACCAAGAACGTGTTCATTGAGGCGACGGC
GACGGATAAGCAGAAGGCGTGCGTGGTCCTTGACACCATCGTCACCCTCTTCTCCCAATACTGCCAAAAACCGTTCCATGTCGAACAGGTGGAG
ATAGAATACGAGGAGACTGGAGAAAAGGATATTTTTCCTTTTTTCAATCAAAAAAATAAAAATAAACATGGAAGACCAGTCTTTAAATACGAGG
AAATGGCGATTCTCCTAAACAAGATGTCCCTGAAAGCGGAGATCGCAATGAAATGCGGGCACAAAGTAATTGTGACGTGGCCCACTCCTCCTGG
AGCGCTCCGTGAAATTATCCAAGACGTCGACGTTGGGTACGGCTACGACAAACTGATGAAAAAGCGTCAGgtgcgggtttaaagattatttttc
cctatttgtagcacaaaaatgctggtttcaacttgaaaaccctatttaccataaaaaaaactcttttccatcttgaaaaaccccctattttaatt
tggaaacccgtttttcggccttcaaagctcctttttcaagagaaaaacctcattttccactcgtaaaatcccatttttcaccccttggatccct
ttttctaaggagaaatcctctttttccactcaaaaggccccttttccaatagaaaaccccatttttccagtcttaggatccctttctcctagtg
gaaatcctcttttctacctctaaaatccccatttccaccccttaggatcctttttcttccccctagaatcctcttttctcctccccaaaatcctcct
tttcttccagTCGAACACTGTCGCCGTCGCCTTCTCAATCAACAAGCTCTGCGACAAATTCCGGATCAAAATCGCCACATTCGGATGGACAGAG
GCCCTCAGCTTCGCCCTCTGCTCCCGAGACGACATCTCGACGAAGCTGCGCCTCCCGAAAAAAGTACTCTCGGAAGCCGTCCACATTGGAAATC
CGAAGACACTGGAATTCCAGGTCGCTCGGACCTCACTTCTTCCGGGTCTTCTGAAGACGTTGGCTTCAAATCGTGATATGCCTCTTCCCCTGAA
GCTCTTCGAGCTCCAGGATGTCATTCTGAAAGATGAAAAGATGGGAGCAAGAAACGAGAGACGACTCGCCGCTGTCTACTACAACAAAGCTGCC
GGATTAGAGATTATCCAAGGATTTTTGGATCGAATGATGAGGATGCTGAATGTGAAGCCGATAGGTGATCCTCGAGGATATCAGACTAAAAAGT
TTAATCgtgagtttttactttatgatcttcagggatttccctagatccttcctccccccccaagagatcccatggattcttggaatccctaaaaa
tatccccagaatttcatgggtcctcctccctttctcccatctccttaatccatcctctcttcttccagATGTGACATTCGAGGAAAAGAATTGC
TACGAAGTCCATTCCCCCAGCGGCGAAATCCTCGGACGATACGGAGTGCTCCATTCAGAGGTCACCGCCGCTTTCGGACTCACCCTTCCATGTG
AAGCAATGGAGATTAGCATCTCCTCATTTTTCCCAAACTAA

>HYDE-1
MSNAPSSTDAPLAENDYDDLFAYGQRPPTPQLPRPATPIDSGPELDEITYESAEEEEVEVYTDVNDLHWSSVEALSRATRIFNEDSILQRYEFG
DVVTRQKLIVKSKINLKVIAVVIRDVSFDEDSYASFIDLQDKLHQNICRKRTLVAIGTHDLDLVQGPFELRAEAPSKIKFCLPNQTKEYTATEM
MQYFSGFSNNFRAMPQLPVLYDKTERICSMPPIINGEHSKITIKTKNVFIEATATDKQKACVVLDTIVTLFSQYCQKPFHVEQVEIEYEETGEK
DIFPFFNQKNKNKHGRPVFKYEEMAILLNKMSLKAEIAMKCGHKVIVTWPTPPGALREIIQDVDVGYGYDKLMKKRQSNTVAVAFSINKLCDKF
RIKIATFGWTEALSFALCSRDDISTKLRLPKKVLSEAVHIGNPKTLEFQVARTSLLPGLLKTLASNRDMPLPLKLFELQDVILKDEKMGARNER
RLAAVYYNKAAGLEIIQGFLDRMMRMLNVKPIGDPRGYQTKKFNHVTFEEKNCYEVHSPSGEILGRYGVLHSEVTAAFGLTLPCEAMEISISSF
FPN

>kss-3.1
ATGGAATTCCCTCTTATCCAACTGCCAGATCTCCCCAAAATTCAAATTATTCAATTGATGTCTATTGGGGAGAAgtaagtattactagagagtt
tcccaagacactcatttatttccagAATCAAACTGGCTCTTTCTTCTCGAAAAATGGAGAACTATCTGGCGTGGGTGTTCAAAAATCATATGCT
CGATGTGGATTGTTTCATTTTTTTGCGAGGCTACCAGTCTATTATCAATATAGATAACAACGAATGGAAACTGTGTTTGAACCGTTTAAAAAAG
GGTTGTGAAAATCCAGGTTATATTAAAGTGGAAGAATTAGAACCGTGgtgagttctgagaagtttcaacaaaattcagtaaaagaatacataat
ttcagGATAAACGATGAGTGGACAATGGTAGAGAAAACTTTCAATGTGTTCATACGTCTTCAAAAAGTTCTTCCGTGCCAGTTTACCCATTTGT
GCGTAATTCTGAATGTCGAACCGACGCCGATTATAGAAATTCTGAGTTATCCGTGCGTCGCGAACTTGACAGGTGTGCATTTCTATGGAGGAAC
AGTTCAGAGGAACGAGTTGGATGCAATTATGGAATGGAGAAAAGAGAACACGATGCAGTATATTTCGGTTACAGATAACGAAATTCCATTGGAT
TATAGACATCCGAATgtgagccttcttttgaatagttatatcctatcaaaacacaaaaagtgcagaatgtttcgctgagcaatttcttagttga
ccgtattctaatatctccactcttaaccaaaatacataaatagattcagGCATTTCGTTTCACTGGAGTAGTGTATGGCGATGCTCGATGGATA
CGTCTCGAAGATTTACTCTCGATGAGAAACTTCTATCGACCGATTTTCAGTGTGAATAATTTCAGTATGAAGGATCTGAACACATTTATTAAAT
ATTGGATCAACTGCGATGAGAATATGTTCACGTATCTAGTTATTGATTATAAAAATATCAATTTATCAGAATTAATGAAGGATATCACTGTTCT

CAAGGGATTCCGATCAGAAAAACCATTTTATCTGATgttagttccttctttttatattcagagaatgtttaagactaattaattgggctcccg
gacatttcgaaacacaacaacattcgagttttcaaatgtcctgtttcgaaaagtgaaggtacgaacgagatcaaaggggttcgagatgtccggg
agccactaattgatcaaaagaaaaatggtttccagAGCATCGAATTCAACTGAAAACCAACTCCTATTGACAATTCAATTGATAGAACACACTG
AATCTCGAAGACGAAATTCGCTTGACTTCTCGATTCAGTATGCCAATGAACCTCATAAATTCATAAACGACGACACACTACCTGCATGGACTCG
AGAATACCGTATTCTGGGACGATTGGCGAGAAAACGTTGGTTGGAAGAGGAGATGATTACTGTAGGAATATCGATTGAAGAAATAAAAGAACTC
GATGATTTGGAGGAGGAGTTGACAGTGGATGAAGTTCGGATCATTGATGGAATAATGACTGTTCAAGAGAAATTGTAA

>KSS-3.1
MEFPLIQLPDLPKIQIIQLMSIGEKIKLALSSRKMENYLAWVFKNHMLDVDCFIFLRGYQSIINIDNNEWKLCLNRLKKGCENPGYIKVEELEP
WINDEWTMVEKTFNVFIRLQKVLPCQFTHLCVILNVEPTPIIEILSYPCVANLTGVHFYGGTVQRNELDAIMEWRKENTMQYISVTDNEIPLDY
RHPNAFRFTGVVYGDARWIRLEDLLSMRNFYRPIFSVNNFSMKDLNTFIKYWINCDENMFTYLVIDYKNINLSELMKDITVLKGFRSEKPFYLI
ASNSTENQLLLTIQLIEHTESRRRNSLDFSIQYANEPHKFINDDTLPAWTREYRILGRLARKRWLEEEMITVGISIEEIKELDDLEEELTVDEV
RIIDGIMTVQEKL

>kss-3.4
ATGGAATTCCCTCTTATCCAACTGCCAGATCTCCCCAAAATTCAAATTATTCAATTGATGTCTATTGGGGAGAAgtaagtattactagagagtt
tcccaagacactcatttatttccagAATCAAACTGGCTCTTTCTTCTCGAAAAATGGAGAACTATCTGGCGTGGGTGTTCAAAAATCATATGCT
CGATGTGGATTGTTTTATTTTTTTGCGAGGATACCAGTCTATTATCAATATAGATAACAACGAATGGAAACTGTGTTTGAATCGTTTAAAAAAG
GGTTGTGAAAATCCAGGCTATATTAAAGTGGAAGAATTAGAACCGTGgtgagttctgagaagtttcaacaaaattcagtaaaaaaatacggtgt
tttagGATAAACGATGAGTGGACAATGGTAGAGAAAACTTTCAATGTGTTCATACGTCTTCAAAAAGTTCTTCCGTGCCAGTTTACCCATTTGT
GCGTAATTCTGAATGTCGAACCGACGCCGATTATAGAAATTCTGAGTTATCCGTGCGTCGCGAACTTGACAGGTGTGCATTTCTATGGAGGAAC
AGTTCAGAGGAACGAGTTGGATGCAATTATGGAATGGAGAAAAGAGAACACGATGCAGTATATTTCGGTTACAGATAACGAAATTCCATTGGAT
TATAGACATCCGAATgtgagccttcttttgaatagttatatcctatcaaaacacaaaaagtgcagaatgtttcgctgagcaatttcttagttga
ccgtattctaatatctccactcttaaccaaaatacataaatagattcagGCATTTCGTTTCACTGGAGTAGTGTATGGCGATGCTCGATGGATA
AGTCTCGAAGATTTACTCTCGATGAGAAACTTCTATCGACCGATTTTCAGTGTGAATAATTTCAGTATGAAGGATCTGAACACATTTATTAAAT
ATTGGATCAACTGCGATGAGAATATGTTCACGTATCTAGTTATTGATTATAAAAATATCAATTTATCAGAATTAATGAAGGATATCACTGTTCT
CAAGGGTTTCCGATCAGAAAAACCATTTTATCTGATgttagttcattctttttattttcagagaatacttaataataattaattttttttca
aacttttctgtctgaacctgaggtgccaaacatcctgaaaaggtccaataagagcgagatgctcatggtcatgttactaaatgttaacaaacaa
tgcaccgaacccaggatcctctaatcctactctgtaataattaattgatcaaaataagaaagaaatggtttccagAGCATCGAATTCAACTGAA
AACCAACTCCTATTGACAATTCAATTGATAGAACACACTGAATCCCGAAGACGAAATTCGCTTGACTTCTCGATTCAGTATGCCAATGAACCTC
ATAAATTCATAAACGACGACACACTACCTGCATGGACTCGAGAATACCGTATTCTGGGACGATTGGCGAGAAAACGTTGGTTGGAAGAGGAGAT
GATTACTGTAGGAATATCGATTGAAGAAATAAAAGAACTCGATGATTTGGAGGAGGAGTTGACAGTGGATGAAGTTCGGATCATTGATGGAATA
ATGACTGTTCAAGAGAAATTGTAA

>KSS-3.4
MEFPLIQLPDLPKIQIIQLMSIGEKIKLALSSRKMENYLAWVFKNHMLDVDCFIFLRGYQSIINIDNNEWKLCLNRLKKGCENPGYIKVEELEP
WINDEWTMVEKTFNVFIRLQKVLPCQFTHLCVILNVEPTPIIEILSYPCVANLTGVHFYGGTVQRNELDAIMEWRKENTMQYISVTDNEIPLDY
RHPNAFRFTGVVYGDARWISLEDLLSMRNFYRPIFSVNNFSMKDLNTFIKYWINCDENMFTYLVIDYKNINLSELMKDITVLKGFRSEKPFYLI
ASNSTENQLLLTIQLIEHTESRRRNSLDFSIQYANEPHKFINDDTLPAWTREYRILGRLARKRWLEEEMITVGISIEEIKELDDLEEELTVDEV
RIIDGIMTVQEKL

>NIC-kss-3.4
ATGGAATTTTCTTTTATTCGACTCCCAGATGTTCCAAAAATTCAAATTATCCAATTGATGACTATTGGGGAGAAgtaagtatctctagagaggt
tcccaagacactcatttattttcagAATCAAACTGGCCCTTTCCTCTCGAAAAATGGAGAACTATCTGGCGTGGGTGTTCAAAAATCAAGTGCT
CAATGTGGATTGTTTCATTTTTTTGCGAGGCTACCAGTCTATTATCAATATAGATAACAACGAATGGAAACTGTGTTTGAACCGTTTAAAAAAG
GGTTGTGAAAATCCAGGTTATATTAAAATGGAAGAATTGAAACCGTGgtgagcaatatcgagaaattcagtgaaagaatacggtgttttagGAT
AAACGATGAGTGGACAATGATAGAGAAAACTTTCAATGTGTTCATACGTCTCCAAAAGGTTCTTCCGTGCCACTTTACCCATTTGTGCGTAATT
CTGAATGTCGAACCGACGACGATTCTAGAAATTCTGAGTTATCCGTGCGTCGCGAACTTAAAAGGTGTGCATTTCTATGGAGGAAAAGTGAAAA
GAAACGAGTTGGATGCAATTATGGAATGGAGAAAAGAGAATACGATTCAGTATATTTCAGTTACAGATAACGAAATTCCATTGGATTATAGACA
TCCGAATgtgagcctttttttcaaatagttacatcctatcagttaaaaaaatgtgcagaatgttccgctgagcaatttcttagttgaccgtattc
tattatcttcattcttaaccgaaatacataaatatattcagGCATTTCGTTTCACTGGAGTTGTGTATGGTGATGCTCGATGGATACGTCTCGA
AGATTTACTCTCGATGAGAAACTTCTATCGACCGATTTTCGGAAAAAATAATTTCAGTATGAAGGATCTGAACACATTTATTAAATATTGGATC
AACTGCGAAGAGAATATGTTCACGTATCTAGTTATTGATTACGAAAATATCAATTTATCAGAATTAATGAAGGATATCACTGTTCTCAAGGGTT
TCCGATCAGAAAAACCATTTTATCTGATgttagtttcttctttttatattcagataatacttattattaattaattgatcaaaagaaaaaaga
aacaattttcagAGCATCGAATTCAACTGAAAACCAACTCTTATTGACAATTCAATTAATAGAACACACTGAATCCCGAAGACGAAATTCGCTT
GACTTCTCGATTCAGTATGCTAACAAACCACATAAATTCATAAACGACGACACACTACCTGCATGGACTCGAGAATACCGTATTCTGGGGCGAT
TGGCGAGAAAACGTTGGTTAGAAGAGGAAATGATTACTGTAGGAATATCCATTGAAGAAATAAAAGAACTCGATGATTTGGAGGAGGAGTTGAC
TGTGGAGGAAGTTCGGATGATTAATGGAATAATGACTGTCAAAGAGAAATTGTAA

>NIC-KSS-3.4
MEFSFIRLPDVPKIQIIQLMTIGEKIKLALSSRKMENYLAWVFKNQVLNVDCFIFLRGYQSIINIDNNEWKLCLNRLKKGCENPGYIKMEELKP
WINDEWTMIEKTFNVFIRLQKVLPCHFTHLCVILNVEPTTILEILSYPCVANLKGVHFYGGKVKRNELDAIMEWRKENTIQYISVTDNEIPLDY
RHPNAFRFTGVVYGDARWIRLEDLLSMRNFYRPIFGKNNFSMKDLNTFIKYWINCEENMFTYLVIDYENINLSELMKDITVLKGFRSEKPFYLI
ASNSTENQLLLTIQLIEHTESRRRNSLDFSIQYANKPHKFINDDTLPAWTREYRILGRLARKRWLEEEMITVGISIEEIKELDDLEEELTVEEV
RMINGIMTVKEKL

>EG-KSS-1
MEFPLIRLPDVPKIQIIQLMSIGERIKLALSSRKMENYLEWVFKNRALDAVYTIELRGDTSFIGIDTKTWVLCMNHFKPECKKPGYISVEELKP
WIDEKWTMMERTFNVFIRLQKVIPCELTQLFVTLKTEPTPILEILSYPCVANLTGVHFFGGTVQRKELDAIMEWRKENTIQYISVTDNEIPLDY
RHPNAFRFTGVVYGDARWISLEDLLSMRNFYRPIFGKNRFSMKDLNTFIKYWINCDENMFTYLVIDYKDINISELMKDITVLKSFRSEKPFYLI
ASNSTQILLTIQLIEHTEPQIRNVLDFSIQYANEPHKFRRGGTVPAWTREYRILGRLARKRWLEEEMITVGISIEEIKELDDLEEELTVEEVRM
IDGIMTVQEKL

>EG-PZL-1
MWSSTTSSHQNQQKIHEKVYKNEHQVRVPPCELKREEYEPKKSFYEMHQQTKRWTNWESQRIVTAPGHCATVDSVLLFEKNDNKFCLSESRDRV
IRFWDVDNVERGVDSVANPWTVAQDDMAQLEWSWNMARDSNTDRFYSTSWDSTVKSWAITDNGAIQNLNTVNVGSAVQAVSCSGNENEIVCTTF
AKRTAVIDSRTFGIVAEHSLHKRAVIGLAVKENKIFTCGEDRLMMMVDRRNMSRPVLFEYCQDAYKSSLSLQRNQLLTSTSDGKVKLYDATNFH

```
DFQTYSVGAFTRQSLLQHGAHFVMARCKRGYYKFLMNSPGIRSPKRCSSHQLTAKPAKFDYSSEMKTLTIGNSMDRSERRSMISEDLDEKFEVE
VDTCPHLPTNFETMKWQERRRSLESFLQTLTGKKCILSNISYDASIKKLQKIIRNDANIFGQVLAIRSVKWVATELGAEFSKFSVSLLPDLLEK
MKEKKQILRKPLIRCTLEVGKTLPLEDGIPVILSALSMANPEIKKQTMLFVVQQLEAMQMERLKGFIPSLLPVLVELTKNATQDVREVAVHALE
SIYWKIGRCRMQSLLSKCRHLLPKRHPWHWIKKGSDEKKKTEKPPSSFFHEAIGSIIYSGEELHAARFSRSNCIRVDLEDQFIRPNLLVGLLKI
LETHAADTTSLNLYECSDVLVRDDTEKNGAREERRLAAAILNESADFAAIVNCLIKLLDALKLSPTKDGDNFHLEISDNQTFSPGHYARMVGPN
DVFLGHVGVVHPEVLRKFNLMLPVAAFEIKIFTEDD

>NIC-HYDE-1
MAERRIYENVVGDIGNLYDEIAHVRLPAAPIPTPRLNLPTVPLPANRYDLLSVEGLSRAIRIFKQEVESPEYRFSDTKTRQKIIVKRETAQVRP
YVVGVILSDVCFDEDSYASFIRIQDKLHQNICRKRTLVAIGTHDLDTIQGPFEYRAEAPNKIKFRPLNQTKDYTAEELMTLYSKDKNMKDTIKT
FKKKSLVPVIYDKNGVVCSMPPFISGAHSEITQKTKNVFIEATATDKQKAYVVLDTIVTLFSQYCQKPFHVEQVEVEYEDKNEKEYFPFTCSKK
MKNNTPEIRTKIVLNFKDEEMAILLNKMSLKAEVASKGGLKVLVPTTRHDILQACDLGEDVGVAYGRKFFVTKLHESNSVAVAVTSPFNYLCDN
LRIKLSVFGWTEALNFALCSRDDISTKLRLPDALSEAVHTLEIRRHWNSKSLGPLFFRVF
```

Protein sequence alignments of FARS-3-derived toxins to FARS-3 (local alignment)

KLMT-1 vs FARS-3

```
Query: KLMT-1 Query ID: lcl|Query_6638871 Length: 503
>EG6180-FARS-3
Sequence ID: Query_6638873 Length: 590
Range 1: 38 to 381

Score:359 bits(922), Expect:1e-122,
Method:Compositional matrix adjust.,
Identities:200/360(56%), Positives:252/360(70%), Gaps:21/360(5%)

Query  48   VTSEKTAVEKDRRESMDKKHMDKKHGETAVGEDLNDQEIYKID---KRYDLLSSEGLSRA  104
            +TSEK AVEK+R             GE A GEDLNDQE+YKID    RYDLLS EGLSRA
Sbjct  38   ITSEKAAVEKER-------------GEAAAGEDLNDQEVYKIDIPANRYDLLSVEGLSRA  84

Query  105  MRIFKQHFEFPDFRFLDAKSIEKINVAANASVFHPYLSGAIVRQISLDLESL-RFLNGLV  163
            +RIFKQ  E P++RF D K+ +KI V    +   PY+ GA++R +S D +S   F++
Sbjct  85   IRIFKQEIESPEYRFSDTKTRQKIIVKRETAQVRPYVVGAVLRDVSFDADSYASFIDLQD  144

Query  164  ADNQETGKKRMQYFITAQDLDKMSGPFEYRAEASNVIKFRPLNQTKEHTADELMTLYSSD  223
              +Q   +KR    I   DLD + GPFEYRAEA N IKFRPLNQTKE+TA+ELMTLYS+D
Sbjct  145  KLHQNICRKRTLVAIGTHDLDTIQGPFEYRAEAPNKIKFRPLNQTKEYTAEELMTLYSTD  204

Query  224  SHMTDFLQLIQNLPVYPVIADKNGLI-SLVPVVDAEPMKISIETKSLMIIVTSVDKENGI  282
            SH+  +L +IQN PVYPVI DKNG++ S+ P+++ E  KI+++TK++ I  T+ DK+
Sbjct  205  SHLKAYLPIIQNHPVYPVIYDKNGVVCSMPPIINGEHSKITLKTKNVFIEATATDKQKAY  264

Query  283  RILNNVLAAISIRIEKPLVVEPVLIEYEKFEEPRALELSPPLSYRKMTVTTPEINTKIGL  342
             +L+ ++   S   +KP  VE V +EYE+  E    EL P LSYR+MTVTTPEINTKIGL
Sbjct  265  VVLDTIVTLFSQYCQKPFHVEQVEVEYEETGEK---ELYPLLSYREMTVTTPEINTKIGL  321

Query  343  NLQDEEMANLLNNMSLKAEVASKEVLEVMIPPTRHDILHACDISEDVGVACAYKNFLRDL  402
            NL+DEEMA LLN MSLKAEV SK VL+V++PPTRHDILHACDI+EDVGVA  Y N +  L
Sbjct  322  NLKDEEMAILLNKMSLKAEVTSKGVLKVVVPPTRHDILHACDIAEDVGVAYGYNNLVTKL  381
```

PZL-1 vs FARS-3

```
Query: PZL-1 Query ID: lcl|Query_6971809 Length: 788
>EG6180-FARS-3
Sequence ID: Query_6971811 Length: 590
Range 1: 445 to 586

Score:127 bits(319), Expect:7e-35,
Method:Compositional matrix adjust.,
Identities:63/142(44%), Positives:87/142(61%), Gaps:0/142(0%)

Query  642  LEDQFIRPNLLVGLLKILETHAADTTSLNLYECSDVLVRDDTEKNGAREERRLAAAILNE  701
            LE Q  R +LL GLLK L ++      L L+E  DV+++D+    GAR ERRLAA   N+
Sbjct  445  LEFQVARTSLLPGLLKTLASNRDMPLPLKLFELQDVILKDEKMDVGARNERRLAAVYYNK  504

Query  702  SADFAAIVNCLIKLLDALKLSPTKDGDNFHLEISDNQTFSPGHYARMVGPNDVFLGHVGV  761
            +A F  I   L +++  L ++PTKD   +H+E  +N TF PG  AR++GPN VFLG +G
Sbjct  505  AAGFEIIQGFLDRMMRMLNVNPTKDQKGYHIEADENPTFFPGRCARIIGPNGVFLGRIGA  564

Query  762  VHPEVLRKFNLMLPVAAFEIKI  783
            +HPEV+  F L LP  A E  +
Sbjct  565  LHPEVITSFGLTLPCGAVEFNV  586
```

HYDE-1 vs FARS-3

```
Query: HYDE-1 Query ID: lcl|Query_6745795 Length: 567
>EG6180-FARS-3
Sequence ID: Query_6745797 Length: 590
Range 1: 32 to 590

Score:633 bits(1633), Expect:0.0,
Method:Compositional matrix adjust.,
Identities:359/561(64%), Positives:402/561(71%), Gaps:39/561(6%)

Query  42   GPELDEITYESA----EEEEVEVYTDVND------------LHWSSVEALSRATRIFNED  85
            G ELDEIT E A    E  E     D+ND                SVE LSRA RIF ++
Sbjct  32   GLELDEITSEKAAVEKERGEAAAGEDLNDQEVYKIDIPANRYDLLSVEGLSRAIRIFKQE  91

Query  86   SILQRYEFGDVVTRQKLIVK---SKINLKVIAVVIRDVSFDEDSYASFIDLQDKLHQNIC  142
                 Y F D  TRQK+IVK   +++   V+  V+RDVSFD DSYASFIDLQDKLHQNIC
Sbjct  92   IESPEYRFSDTKTRQKIIVKRETAQVRPYVVGAVLRDVSFDADSYASFIDLQDKLHQNIC  151

Query  143  RKRTLVAIGTHDLDLVQGPFELRAEAPSKIKFCLPNQTKEYTATEMMQYFSGFSN-----  197
            RKRTLVAIGTHDLD +QGPFE RAEAP+KIKF   NQTKEYTA E+M  +S  S+
Sbjct  152  RKRTLVAIGTHDLDTIQGPFEYRAEAPNKIKFRPLNQTKEYTAEELMTLYSTDSHLKAYL  211

Query  198  -NFRAMPQLPVLYDKTERICSMPPIINGEHSKITIKTKNVFIEATATDKQKACVVLDTIV  256
               +  P  PV+YDK   +CSMPPIINGEHSKIT+KTKNVFIEATATDKQKA VVLDTIV
Sbjct  212  PIIQNHPVYPVIYDKNGVVCSMPPIINGEHSKITLKTKNVFIEATATDKQKAYVVLDTIV  271

Query  257  TLFSQYCQKPFHVEQVEIEYEETGEKDIFPFFNQKNKNKHGRPV-------FKYEEMAIL  309
            TLFSQYCQKPFHVEQVE+EYEETGEK+++P  + +        +        K EEMAIL
Sbjct  272  TLFSQYCQKPFHVEQVEVEYEETGEKELYPLLSYREMTVTTPEINTKIGLNLKDEEMAIL  331

Query  310  LNKMSLKAEIAMKCGHKVIVTWPTPPGALR--EIIQDVDVGYGYDKLMKKR-QSNTVAVA  366
            LNKMSLKAE+  K   KV+V  PT    L   +I +DV V YGY+ L+ K  +SNTVAVA
Sbjct  332  LNKMSLKAEVTSKGVLKVVVP-PTRHDILHACDIAEDVGVAYGYNNLVTKLPESNTVAVA  390

Query  367  FSINKLCDKFRIKIATFGWTEALSFALCSRDDISTKLRLPKKVLSEAVHIGNPKTLEFQV  426
            F INKLCD  RI+IA  GWTEAL+FALCSRDDISTKLRLP   LSEAVHIGNPKTLEFQV
Sbjct  391  FPINKLCDNLRIEIAAAGWTEALNFALCSRDDISTKLRLP-DALSEAVHIGNPKTLEFQV  449

Query  427  ARTSLLPGLLKTLASNRDMPLPLKLFELQDVILKDEKM--GARNERRLAAVYYNKAAGLE  484
            ARTSLLPGLLKTLASNRDMPLPLKLFELQDVILKDEKM  GARNERRLAAVYYNKAAG E
Sbjct  450  ARTSLLPGLLKTLASNRDMPLPLKLFELQDVILKDEKMDVGARNERRLAAVYYNKAAGFE  509

Query  485  IIQGFLDRMMRMLNVKPIGDPRGYQTKKFNHVTFEEKNCYEVHSPSGEILGRYGVLHSEV  544
            IIQGFLDRMMRMLNV P  D +GY  +   + TF    C  + P+G  LGR G LH EV
Sbjct  510  IIQGFLDRMMRMLNVNPTKDQKGYHIEADENPTFFPGRCARIIGPNGVFLGRIGALHPEV  569

Query  545  TAAFGLTLPCEAMEISISSFF  565
              +FGLTLPC A+E ++  F
Sbjct  570  ITSFGLTLPCGAVEFNVEPFL  590
```

Protein sequences of *C. tropicalis* FARS-3 and FARS-3-derived regions of KLMT-1, PZL-1 and HYDE-1 used for phylogenetic tree in Fig. 3f and for pairwise alignments in Supplementary Fig. 4.

```
>Ctr_FARS-3
MPTVGIKKVLLDKHFGRIYTEKEFDELCFEYGLELDEITSEKAAVEKERGEAAAGEDLNDQEVYKIDIPANRYDLLSVEGLSRAIRIFKQEIES
PEYRFSDTKTRQKIIVKRETAQVRPYVVGAVLRDVSFDADSYASFIDLQDKLHQNICRKRTLVAIGTHDLDTIQGPFEYRAEAPNKIKFRPLNQ
TKEYTAEELMTLYSTDSHLKAYLPIIQNHPVYPVIYDKNGVVCSMPPIINGEHSKITLKTKNVFIEATATDKQKAYVVLDTIVTLFSQYCQKPF
HVEQVEVEYEETGEKELYPLLSYREMTVTTPEINTKIGLNLKDEEMAILLNKMSLKAEVTSKGVLKVVVPPTRHDILHACDIAEDVGVAYGYNN
LVTKLPESNTVAVAFPINKLCDNLRIEIAAAGWTEALNFALCSRDDISTKLRLPDALSEAVHIGNPKTLEFQVARTSLLPGLLKTLASNRDMPL
PLKLFELQDVILKDEKMDVGARNERRLAAVYYNKAAGFEIIQGFLDRMMRMLNVNPTKDQKGYHIEADENPTFFPGRCARIIGPNGVFLGRIGA
LHPEVITSFGLTLPCGAVEFNVEPFL

>KLMT-1
TSEKTAVEKDRRESMDKKHMDKKHGETAVGEDLNDQEIYKIDKRYDLLSSEGLSRAMRIFKQHFEFPDFRFLDAKSIEKINVAANASVFHPYLS
GAIVRQISLDLESLRFLNGLVADNQETGKKRMQYFITAQDLDKMSGPFEYRAEASNVIKFRPLNQTKEHTADELMTLYSSDSHMTDFLQLIQNL
PVYPVIADKNGLISLVPVVDAEPMKISIETKSLMIIVTSVDKENGIRILNNVLAAISIRIEKPLVVEPVLIEYEKFEEPRALELSPPLSYRKMT
VTTPEINTKIGLNLQDEEMANLLNNMSLKAEVASKEVLEVMIPPTRHDILHACDISEDVGVACAYKNFLRDLSK

>PZL-1
LEDQFIRPNLLVGLLKILETHAADTTSLNLYECSDVLVRDDTEKNGAREERRLAAAILNESADFAAIVNCLIKLLDALKLSPTKDGDNFHLEIS
DNQTFSPGHYARMVGPNDVFLGHVGVVHPEVLRKFNLMLPVAAFEIKIFTEDD
```

>HYDE-1
TYESAEEEEVEVYTDVNDLHWSSVEALSRATRIFNEDSILQRYEFGDVVTRQKLIVKSKINLKVIAVVIRDVSFDEDSYASFIDLQDKLHQNIC
RKRTLVAIGTHDLDLVQGPFELRAEAPSKIKFCLPNQTKEYTATEMMQYFSGFSNNFRAMPQLPVLYDKTERICSMPPIINGEHSKITIKTKNV
FIEATATDKQKACVVLDTIVTLFSQYCQKPFHVEQVEIEYEETGEKDIFPFFNQKNKNKHGRPVFKYEEMAILLNKMSLKAEIAMKCGHKVIVT
WPTPPGALREIIQDVDVGYGYDKLMKKRQSNTVAVAFSINKLCDKFRIKIATFGWTEALSFALCSRDDISTKLRLPKKVLSEAVHIGNPKTLEF
QVARTSLLPGLLKTLASNRDMPLPLKLFELQDVILKDEKMGARNERRLAAVYYNKAAGLEIIQGFLDRMMRMLNVKPIGDPRGYQTKKFNHVTF
EEKNCYEVHSPSGEILGRYGVLHSEVTAAFGLTLPCEAMEISISSFFPN

Supplementary Table 1. Raw data for all phenotyping of lines used in this study.

Strain	Short name	Mothers screened	Total number of embryos	Embryonic lethal	Larval arrest	Delay	Arrest	Sterile	Other	Wild-type (WT)	% of WT	Comments
**QX2341**	Chr. II NIL	10	100	4	0	2	1	0	0	93	93,0	
**QX2343**	Chr. V NIL	10	100	1	0	0	0	0	0	99	99,0	
**INK303**	NIC-ORF015419(-) = *klmt-1(-)*	10	100	2	0	3	0	0	0	95	95,0	
**INK324**	NIC-ORF006816 (-) = *pzl-1(-)*	10	100	0	2	2	3	0	0	93	93,0	
**INK422**	NIC-ORF015419(-) NIC-ORF015420(-) = *klmt-1(-) kss-1(-)*	10	100	4	0	0	0	0	0	96	96,0	
**INK485**	NIC-ORF006816 (-) NIC-ORF006815 (-) = *plz-1(-) kss-2(-)*	10	100	1	1	1	0	0	0	97	97,0	
**INK722**	FARS-3::mScarlet	8	119	0	0	1	1	1	0	116	97,5	
**INK793**	*kss-3.4(-)*	8	80	1	1	0	0	0	0	78	97,5	
**INK801**	*kss-3.3(-) kss-3.4(-)*	10	100	1	0	6	2	0	0	91	91,0	
**INK951**	*kss-3.1(-) kss-3.2(-)*	11	110	1	0	0	0	0	0	109	99,1	
**INK957**	*kss-3.2(-) kss-3.3(-) kss-3.4(-)*	8	80	3	0	0	0	0	0	77	96,3	
**INK1164**	*hyde-1(-) kss-3.1-3.4(-)*	10	100	4	1	3	0	0	0	92	92,0	
**INK1088**	mScarlet::ZYG-9.2	10	100	2	0	3	0	0	0	95	95,0	
**INK1169**	*kss-3.1(-) kss-3.2(-) kss-3.3(-)*	10	100	1	1	1	0	0	3	94	94,0	Other = worms not found
**INK169**	KLMT-1::mNeonGreen = KLMT-1::mNG	10	100	2	0	1	0	1	2	94	94,0	Other = worms not found
**INK206**	KLMT-1::mNeonGreen *kss-1(-)*	10	100	1	0	2	0	1	2	94	94,0	Other = worms not found

**INK950**	*kss-3.1-3.4(-)* FARS-3::mScarlet	18	180	62	41	23	3	10	0	41	22,8	Day 1 - 52 affected larvae, day 4 - 11/52 wild type worms
**INK1196**	*kss-3.1(-) kss-3.4(-)*	10	100	69	15	2	3	0	0	11	11,0	Day 1 - 24 affected larvae, day 4 - 4/24 wild type worms, 5/24 delay and arrest
**INK1172***	*kss-3.1(-) kss-3.2(-) kss-3.4(-)*	4	32	-	-	-	-	-	-	-	-	See footnote
**INK1091**	EMS-2	4	60	8	2	0	2	not screened	0	48	80,0	Revertant, sequenced
**INK1092**	EMS-3; *hyde-1(abu529[E277K]) II*	4	61	5	0	0	2	not screened	0	54	88,5	Revertant, sequenced
**INK1095**	EMS-6; *hyde-1(abu532[P286L]) II*	4	61	2	0	1	0	not screened	0	58	95,1	Revertant, sequenced
**INK1097**	EMS-8	4	60	5	2	0	0	not screened	0	53	88,3	Revertant, sequenced
**INK1100**	EMS-11	4	60	5	0	1	0	not screened	0	54	90,0	Revertant, sequenced.
**EG6180**	EG6180	10	100	2	0	0	1	0	0	97	97,0	Wild type control
**INK950**	*kss-3.1-3.4(-)* FARS-3::mScarlet	18	180	62	41	23	3	10	0	41	22,8	Day 1 - 52 affected larvae, day 4 - 11/52 wild type worms

*We aimed to knock-out *kss-3.4* in the backrground of *kss-3.1(-) kss-3.1(-)* using CRISPR/Cas. After injection we selected 4 heterozygous for *kss-3.4(-)* hermaphrodites, propagated them and singled 32 offspring on individual plates, expecting to recover ~25% of desired genotype according to Mendelian segregation. 4 days later we genotyped 32 lines: 21 were heterozygous for *kss-3.4(-)* allele, 11 homozygous for wild type allele, and we did not recover *kss-3.4(-)* homozygous offspring, which suggested that this allele in homozygous state was detrimental. We decided not to proceed with isolating affected offspring as described in 'Generation of *C. tropicalis* transgenic lines' Methods section.

Supplementary Table 2. List of all *C. tropicalis* strains used in this study. All strains except QX2341, QX2343, NIC203 and EG6180 were generated for this study.

Strain	Short name	Genotype	Description
**QX2341**	Chr. II NIL	*qqIR45 (II:8.0-8.8 Mb; NIC203 > EG6180); EG6180 Mito*	NIL carrying NIC203 TA element on Chr. II in a EG6180 background. Source - Ben-David et al (2021)
**QX2343**	Chr. V NIL	*qqIR47 (V:1.3-1.8 Mb; NIC203 > EG6180); EG6180 Mito*	NIL carrying NIC203 TA element on Chr. V in a EG6180 background. Source - Ben-David et al (2021)
**NIC203**	NIC203	wild type	Wild isolate from Capesterre Belle-Eau, Guadeloupe. Lat 16.05 Lon -61.63. Found in rotting flowers by N. Poullet and C. Braendle. Source - Christian Braendle
**EG6180**	EG6180	wild type	Wild isolate from El Yunque, Puerto Rico. Lat 18.3 Lon -65.8. Found in rotting fruit by M. Ailion and E. Jorgensen. Source - Christian Braendle
**INK285**	Chr. IV SLP	*abuSi9[dpy-10 & sup-35 gRNA targets::(HygR(p.52-341)::rps-20 3' UTR::LoxP, IV:6.9Mb]*	Chr. IV synthetic landing pad with split hygromycine resistance, EG6180 background. Permissible only for somatic expression
**INK943**	Chr. I SLP	*abuSi33[dpy-10 & sup-35 gRNA targets::(HygR(p.52-341)::rps-20 3' UTR::LoxP, I:7.5Mb]*	Chr. I synthetic landing pad with split hygromycine resistance, EG6180 background. Permissible only for somatic expression
**INK63**	3xFLAG::KLMT-1	*klmt-1(abu32[3xFLAG::klmt-1]) V; qqIR47*	N-terminal 3xFLAG tagged KLMT-1, QX2343 background
**INK66**	KLMT-1::3xFLAG	*klmt-1(abu33[klmt-1::3xFLAG]) V; qqIR47*	C-terminal 3xFLAG tagged KLMT-1, QX2343 background
**INK169**	KLMT-1::mNeonGreen = KLMT-1::mNG	*klmt-1(abu107[klmt-1::2xTy1::mNeonGreen]) V; qqIR47*	C-terminal mNeonGreen tagged KLMT-1 (with 2xTy1 linker), QX2343 background
**INK206**	KLMT-1::mNeonGreen; *kss-1(-)*	*kss-1(abu125[p.R7Ffs*28]) V; klmt-1(abu107[klmt-1::2xTy1::mNeonGreen]) V; qqIR47*	*kss-1* knockout and C-terminal mNeonGreen tagged KLMT-1 (with 2xTy1 linker), QX2343 background
**INK277**	ΔN-KLMT-1::3xFLAG	*klmt-1(abu173[klmt-1::3xFLAG(p.S2_T45del)]) V; qqIR47*	Deletion of N-terminal IDR (internally disordered region) of KLMT-1. N-terminal truncation was chosen based on ANCHOR2 prediction and is 24 amino acids shorter than N-terminal IDR sequence fused to mCherry (INK874)

**INK303**	*NIC-ORF015419(-) = klmt-1(-)*	*klmt-1(abu191[p.E74Gfs*50]) V; qqIR47*	*klmt-1* knockout, QX2343 background
**INK310**	heat-shock inducible mCherry	*abuSi11[hsp-16.11p::mCherry::tbb-2 3' UTR + HygR(+); abuSi9] IV*	Heat-shock inducible expression of mCherry, Chr. IV landing pad, EG6180 background
**INK318**	heat-shock inducible KLMT-1	*abuSi19[hsp-16.11p::klmt-1::tbb-2 3' UTR + HygR(+); abuSi9] IV*	Heat-shock inducible expression of KLMT-1, Chr. IV landing pad, EG6180 background
**INK322**	heat-shock inducible ΔNC-KLMT-1	*abuSi34[hsp-16.11p::klmt-1(p.1M; p.K70_R459)::tbb-2 3' UTR + HygR(+); abuSi9] IV*	Heat-shock inducible expression of KLMT-1 without N- and C-terminal IDRs, Chr. IV landing pad, EG6180 background
**INK324**	*NIC-ORF006816 (-) = pzl-1(-)*	*pzl-1(abu205[p.K446_W447delinsT*]) II; qqIR45*	*pzl-1* knockout, QX2341 background
**INK336**	heat-shock inducible IDR-mCherry-IDR	*abuSi36[hsp-16.11::KLMT-1(p.M1_D69)::mCherry::KLMT-1(p.A460_S503)::tbb-2 3' UTR + HygR(+); abuSi9] IV*	Heat-shock inducible expression of mCherry fused with N- and C-IDRs of KLMT-1
**INK422**	*NIC-ORF015419(-) NIC-ORF015420(-) = klmt-1(-) kss-1(-)*	*klmt-1(abu191[p.E74Gfs*50]) V; kss-1(abu192[p.T229Efs*8]) V; qqIR47*	*klmt-1* and *kss-1* knockout, QX2343 background
**INK438**	heat-shock inducible KSS-1	*abuSi40[hsp-16.11::kss-1::tbb-2 3' UTR + HygR(+), abuSi33] I*	Heat-shock inducible expression of KSS-1, Chr. I landing pad, EG6180 background
**INK444**	3xFLAG::KSS-1	*kss-1(abu278[3xFLAG::kss-1]) V; qqIR47*	N-terminal 3xFLAG tagged KSS-1, QX2343 background
**INK485**	*NIC-ORF006816 (-) NIC-ORF006815 (-) = plz-1(-) kss-2(-)*	*kss-2(abu291[p.Y81*]) II; pzl-1(abu205[p.K446_W447delinsT*]) II; qqIR45*	*pzl-1 and kss-2* knockout, QX2341 background
**INK505**	FARS-3::3xFLAG	*fars-3(abu302[fars-3:3xFLAG]) II*	C-terminal 3xFLAG tagged FARS-3, EG6180 background
**INK512**	heat-shock inducible KSS-1; heat-shock inducible KLMT-1	*abuSi40[hsp-16.11::kss-1::tbb-2 3' UTR + HygR(+), abuSi33] I; abuSi19[hsp-16.11::klmt-1::tbb-2 3' UTR + HygR(+); abuSi9] IV*	Heat-shock inducible expression of KSS-1, Chr. I landing pad, and KLMT-1, Chr. IV landing pad, EG6180 background. Obtained by crossing INK438 to INK318
**INK563**	3xFLAG::KSS-1 (*Ctr-rpl-36p*)	*abuSi53[rpl-36p::3xFLAG::kss-1::rpl-36 3' UTR + HygR(+), abuSi33] I*	Ribosomal promoter driven expression of N-terminal 3xFLAG tagged KSS-1, EG6180 background

**INK581**	KLMT-1-ΔC	*klmt-1(abu346[3xFLAG::klmt-1(p.A460_S503del)]) V; qqIR47*	Deletion of C-terminal IDR of KLMT-1
**INK586**	heat-shock inducible KSS-2	*abuSi56[hsp-16.11::kss-2::tbb-2 3' UTR + HygR(+), abuSi33] I*	Heat-shock inducible expression of KSS-2, Chr. I landing pad, EG6180 background
**INK590**	EG-KSS-2[G297R]	*kss-2(abu335[p.G297R]) II*	EG6180 KSS-2 with glycine 297 changed to arginine, EG6180 background
**INK629**	heat-shock inducible KSS-2; heat-shock inducible KLMT-1	*abuSi56[hsp-16.11::kss-2::tbb-2 3' UTR + HygR(+), abuSi33] I; abuSi19[hsp-16.11::klmt-1::tbb-2 3' UTR + HygR(+); abuSi9] IV*	Heat-shock inducible expression of KSS-2, Chr. I landing pad and KLMT-1, Chr. IV landing pad, EG6180 background. Obtained by crossing INK586 to INK318
**INK631**	3xFLAG::KSS-1 *(Ctr-rpl-36p)*; QX2343	*abuSi53[rpl-36p::3xFLAG::kss-1::rpl-36 3' UTR + HygR(+), abuSi33] I; qqIR47*	Ribosomal promoter driven expression of N-terminal 3xFLAG tagged KSS-1, Chr. I landing pad, QX2343 background. Obtained by crossing INK563 to QX2343
**INK680**	heat-shock inducible PZL-1	*abuSi66[hsp-16.11::pzl-1::tbb-2 3' UTR + HygR(+); abuSi9] IV*	Heat-shock inducible expression of PZL-1, Chr. IV landing pad, EG6180 background
**INK722**	FARS-3::mScarlet	*fars-3(abu397[FARS-3::mScarlet]) II*	C-terminal mScarlet tagged FARS-3, EG6180 background. Used as a background for INK793, INK801, INK919, INK921, INK950, INK951, INK957
**INK726**	heat-shock inducible KSS-2; heat-shock inducible PZL-1	*abuSi56[hsp-16.11::kss-2::tbb-2 3' UTR + HygR(+), abuSi33] I; abuSi66[hsp-16.11::pzl-1::tbb-2 3' UTR + HygR(+); abuSi9] IV*	Heat-shock inducible expression of KSS-2, Chr. I landing pad, and PZL-1, Chr. IV landing pad, EG6180 background. Obtained by crossing INK586 to INK680
**INK732**	heat-shock inducible KSS-1; heat-shock inducible PZL-1	*abuSi40[hsp-16.11::kss-1::tbb-2 3' UTR + HygR(+), abuSi33] I; abuSi66[hsp-16.11::pzl-1::tbb-2 3' UTR + HygR(+); abuSi9] IV*	Heat-shock inducible expression of KSS-1, Chr. I landing pad, and PZL-1, Chr. IV landing pad, EG6180 background. Obtained by crossing INK438 to INK680
**INK767**	3xFLAG::KSS-1 *(Ctr-rpl-36p)*; heat-shock inducible IDR-mCherry-IDR	*abuSi53[rpl-36p::3xFLAG::kss-1::rpl-36 3' UTR + HygR(+), abuSi33] I; abuSi36[hsp-16.11::KLMT-1(p.M1_D69)::mCherry::KLMT-1(p.A460_S503)tbb-2 3' UTR + HygR(+); abuSi9] IV*	Heat-shock inducible expression of mCherry fused with N- and C-IDRs
**INK777**	3xFLAG::KSS-1(INDDTL) *(Ctr-rpl-36p)*	*abuSi69[rpl-36p::3xFLAG::kss-1(R323_V328delinsINDDTL)::rpl-36 3' UTR + HygR(+), abuSi33] I*	Ribosomal promoter driven expression of N-terminal 3xFLAG tagged KSS-1 mutant (323RRGGTV>INDDTL), Chr. I landing pad, EG6180 background
**INK783**	3xFLAG::KSS-1(INDDTL) *(Ctr-rpl-36p)*; QX2343	*abuSi69[rpl-36p::3xFLAG::kss-1(R323_V328delinsINDDTL)::rpl-36 3' UTR + HygR(+), abuSi33] I; qqIR47*	Ribosomal promoter driven expression of N-terminal 3xFLAG tagged KSS-1 mutant (323RRGGTV>INDDTL), Chr. I landing pad, Qx2343 background. Obtained by crossing INK777 to QX2343
**INK785**	3xFLAG::KSS-1 *(Ctr-rpl-36p)*; KLMT-1::mNeonGreen; *kss-1(-)*	*abuSi53[rpl-36p::3xFLAG::kss-1::rpl-36 3' UTR + HygR(+), abuSi33] I; kss-1(abu125[p.R7Ffs*28]) V; klmt-1(abu107[klmt-1::2xTy1::mNeonGreen]) V; qqIR47*	Heat-shock inducible expression of KSS-1 (Chr. I landing pad), endogenous kss-1 knockout and C-terminal mNeonGreen tagged KLMT-1 (with 2xTy1 linker), Qx2343 background. Obtained by crossing INK563 to INK206

**INK793**	*kss-3.4(-)*	*kss-3.4(abu411[p.W100*]) II; fars-3(abu397[fars-3::mScarlet]) II*	*kss-3.4* knockout, C-terminal mScarlet tagged FARS-3, EG6180 background
**INK801**	*kss-3.3(-) kss-3.4(-)*	*kss-3.3(abu416[p.S99*]) II; kss-3.4(abu411[p.W100*]) II; fars-3(abu397[fars-3::mScarlet]) II*	*kss-3.3* and *kss-3.4* knockout, C-terminal mScarlet tagged FARS-3, EG6180 background
**INK806**	3xFLAG::KSS-1(INDDTL) *(Ctr-rpl-36p)*; heat-shock inducible IDR-mCherry-IDR	*abuSi69[rpl-36p::3xFLAG::kss-1(R323_V328delinsINDDTL)::rpl-36 3' UTR + HygR(+), abuSi33] I; abuSi36[hsp-16.11::KLMT-1(p.M1_D69)::mCherry::KLMT-1(p.A460_S503)tbb-2 3' UTR + HygR(+); abuSi9] IV*	Ribosomal promoter driven expression of N-terminal 3xFLAG tagged KSS-1 mutant (323RRGGTV>INDDTL), Chr. I landing pad, and heat-shock inducible expression of mCherry fused with N- and C-IDRs
**INK808**	3xFLAG::KSS-1 *(Ctr-rpl-36p)*; heat-shock inducible mCherry	*abuSi53[rpl-36p::3xFLAG::kss-1::rpl-36 3' UTR + HygR(+), abuSi33] I; abuSi11[hsp-16.11::mCherry::tbb-2 3' UTR + HygR(+); abuSi9] IV*	Ribosomal promoter driven expression of N-terminal 3xFLAG tagged KSS-1, Chr. I landing pad, heat-shock inducible expression of mCherry, Chr. IV landing pad, EG6180 background. Obtained by crossing INK563 to INK310
**INK854**	heat-shock inducible mCherry-IDR	*abuSi71[hsp-16.11::mCherry::KLMT-1(p.A460_S503)::tbb-2 3' UTR + HygR(+); abuSi9] IV*	Heat-shock inducible expression of mCherry fused with C-IDR of KLMT-1
**INK874**	heat-shock inducible IDR-mCherry	*abuSi82[hsp-16.11p::KLMT-1(p.M1_D69)::mCherry::tbb-2 3' UTR + HygR(+)] IV*	Heat-shock inducible expression of mCherry fused with N-IDR of KLMT-1
**INK877**	heat-shock inducible KSS-3.1	*abuSi85[hsp-16.11::kss-3.1::tbb-2 3' UTR + HygR(+), abuSi33] I*	Heat-shock inducible expression of KSS-3.1, Chr. I landing pad, EG6180 background. Used for getting INK919, INK996
**INK880**	heat-shock inducible KSS-3.3	*abuSi88[hsp-16.11::kss-3.3::tbb-2 3' UTR + HygR(+), abuSi33] I*	Heat-shock inducible expression of KSS-3.3, Chr. I landing pad, EG6180 background. Used for getting INK921, INK1011
**INK892**	3xFLAG::KSS-1 *(Ctr-rpl-36p)*; heat-shock inducible mCherry-IDR	*abuSi53[rpl-36p::3xFLAG::kss-1::rpl-36 3' UTR + HygR(+), abuSi33] I; abuSi71[hsp-16.11::mCherry::KLMT-1(p.A460_S503)::tbb-2 3' UTR + HygR(+); abuSi9] IV*	Ribosomal promoter driven expression of N-terminal 3xFLAG tagged KSS-1, Chr. I landing pad, heat-shock inducible expression of mCherry fused with C-IDR. Obtained by crossing INK563 to INK854
**INK894**	3xFLAG::KSS-1 *(Ctr-rpl-36p)*; heat-shock inducible IDR-mCherry	*abuSi53[rpl-36p::3xFLAG::kss-1::rpl-36 3' UTR + HygR(+), abuSi33] I; abuSi82[hsp-16.11p::KLMT-1(p.M1_D69)::mCherry::tbb-2 3' UTR + HygR(+)] IV*	Ribosomal promoter driven expression of N-terminal 3xFLAG tagged KSS-1, Chr. I landing pad, heat-shock inducible expression of mCherry fused with N-IDR. Obtained by crossing INK563 to INK874.
**INK919**	heat-shock inducible KSS-3.1; FARS-3::mScarlet	*abuSi85[hsp-16.11::kss-3.1::tbb-2 3' UTR + HygR(+), abuSi33] I; fars-3(abu397[FARS-3::mScarlet]) II*	Heat-shock inducible expression of KSS-3.1, Chr. I landing pad, C-terminal mScarlet tagged FARS-3, EG6180 background. Obtained by crossing INK722 to INK877
**INK921**	heat-shock inducible KSS-3.3; FARS-3::mScarlet	*abuSi88[hsp-16.11::kss-3.3::tbb-2 3' UTR + HygR(+), abuSi33] I; fars-3(abu397[FARS-3::mScarlet]) II*	Heat-shock inducible expression of KSS-3.3, Chr. I landing pad, C-terminal mScarlet tagged FARS-3, EG6180 background. Obtained by crossing INK722 to INK880
**INK950**	*kss-3.1-3.4(-)* FARS-3::mScarlet	*kss-3.1-3.2(abu490[g.11323197_11326206del]) II; kss-3.3(abu416[p.S99*]) II; kss-3.4(abu411[p.W100*]) II; fars-3(abu397[fars-3::mScarlet]) II*	Knockout of *kss-3.1*, *kss-3.2*, *kss-3.3*, *kss-3.4*, C-terminal mScarlet tagged FARS-3, EG6180 background

**INK951**	*kss-3.1(-) kss-3.2(-)*	*kss-3.1-3.2(abu492[g.11323197_11326206del]) II; fars-3(abu397[fars-3::mScarlet]) II*	Knockout of *kss-3.1*, *kss-3.2*, C-terminal mScarlet tagged FARS-3, EG6180 background
**INK957**	*kss-3.2(-) kss-3.3(-) kss-3.4(-)*	*kss-3.2(abu498[p.W47*]) II; kss-3.3(abu416[p.S99*]) II; kss-3.4(abu411[p.W100*]) II; fars-3(abu397[fars-3::mScarlet]) II*	Knockout of *kss-3.2*, *kss-3.3*, *kss-3.4*, C-terminal mScarlet tagged FARS-3, EG6180 background
**INK976**	3xFLAG::KSS-2 *(Ctr-rpl-36p)*	*abuSi96[rpl-36p::3xFLAG::kss-2::rpl-36 3' UTR + HygR(+), abuSi33] I*	Ribosomal promoter driven expression of N-terminal 3xFLAG tagged KSS-2, Chr. I landing pad, EG6180 background
**INK996**	heat-shock inducible KSS-3.1; *kss-3.1-3.4(-)*	*abuSi85[hsp-16.11::kss-3.1::tbb-2 3' UTR + HygR(+), abuSi33] I; kss-3.1-3.2(abu490[g.11323197_11326206del]) II; kss-3.3(abu416[p.S99*]) II; kss-3.4(abu411[p.W100*]) II; fars-3(abu397[fars-3::mScarlet]) II*	Heat-shock inducible expression of KSS-3.1, Chr. I landing pad, knockout of *kss-3.1, kss-3.2, kss-3.3, kss-3.4*, C-terminal mScarlet tagged FARS-3, EG6180 background. Obtained by crossing INK877 to INK950
**INK1011**	heat-shock inducible KSS-3.3; *kss-3.1-3.4(-)*	*abuSi88[hsp-16.11::kss-3.3::tbb-2 3' UTR + HygR(+), abuSi33] I; kss-3.1-3.2(abu490[g.11323197_11326206del]) II; kss-3.3(abu416[p.S99*]) II; kss-3.4(abu411[p.W100*]) II; fars-3(abu397[fars-3::mScarlet]) II*	Heat-shock inducible expression of KSS-3.3, Chr. I landing pad, knockout of *kss-3.1, kss-3.2, kss-3.3, kss-3.4*, C-terminal mScarlet tagged FARS-3, EG6180 background. Obtained by crossing INK880 to INK950
**INK1030**	heat-shock inducible PZL-1::mCherry	*abuSi124[hsp-16.11p::pzl-1::mCherry::tbb-2 3' UTR + HygR(+)] IV*	Heat-shock inducible expression of PZL-1 tagged with mCherry on C-terminus, Chr. IV landing pad, EG6180 background
**INK1053**	3xFLAG::KSS-2 *(Ctr-rpl-36p)*; heat-shock inducible PZL-1::mCherry	*abuSi96[rpl-36p::3xFLAG::kss-2::rpl-36 3' UTR + HygR(+), abuSi33] I; abuSi124[hsp-16.11p::pzl-1::mCherry::tbb-2 3' UTR + HygR(+)] IV*	Ribosomal promoter driven expression of N-terminal 3xFLAG tagged KSS-2, Chr. I landing pad, heat-shock inducible expression of PZL-1 tagged with mCherry on C-terminus, Chr. IV landing pad, EG6180 background. Obtained by crossing INK976 to INK1030
**INK1064**	Quadruple toxin knock-out	*pzl-1(abu276[p.Q123 *]) II; slow-1(abu277[p.K123_H124delinsND*]) III; klmt-1(abu274[p.M68Tfs*15]) V; NIC-ORF019770(abu275[p.T43Nfs*3]) V;*	Knock-out of four NIC203 toxins, *klmt-1*, *pzl-1*, *slow-1* and *NIC-ORF019770* (not published). NIC203 background
**INK1091**	EMS-2	*EMS mutagenesis line 2*	Revertant line derived from EMS-induced mutagenesis of INK950, EG6180 background
**INK1092**	EMS-3	*hyde-1(abu529[p.E277K]) II; kss-3.1-3.2(abu490[g.11323197_11326206del]) II; kss-3.3(abu416[p.S99*]) II; kss-3.4(abu411[p.W100*]) II; fars-3(abu397[fars-3::mScarlet]) II*	Revertant line derived from EMS-induced mutagenesis of INK950, EG6180 background
**INK1095**	EMS-6	*hyde-1(abu532[p.P286L]) II, kss-3.1-3.2(abu490[g.11323197_11326206del]) II; kss-3.3(abu416[p.S99*]) II; kss-3.4(abu411[p.W100*]) II; fars-3(abu397[fars-3::mScarlet]) II*	Revertant line derived from EMS-induced mutagenesis of INK950, EG6180 background
**INK1097**	EMS-8	*EMS mutagenesis line 8*	Revertant line derived from EMS-induced mutagenesis of INK950, EG6180 background
**INK1100**	EMS-11	*EMS mutagenesis line 11*	Revertant line derived from EMS-induced mutagenesis of INK950, EG6180 background

**INK1088**	mScarlet::ZYG-9.2	*zyg-9.2(abu458[mScarlet:zyg-9.2]) II*	N-terminal mScarlet tagged ZYG-9.2, EG6180 background. Used as a background for INK1164, INK1169, INK1172 and INK1196
**INK1164**	*hyde-1(-) kss-3.1-3.4(-)*	*hyde-1(abu517[P203_L205delinsTN*]) II; kss-3.1-3.2(abu490[g.11323197_11326206del]) II; kss-3.3(abu416[p.S99*]) II; kss-3.4(abu411[p.W100*]) II; fars-3(abu397[fars-3::mScarlet]) II*	*kss-3.1, kss-3.2, kss-3.4* knockout, N-terminal mScarlet tagged ZYG-9.2, EG6180 background
**INK1169**	*kss-3.1(-) kss-3.2(-) kss-3.3(-)*	*zyg-9.2(abu458[mScarlet:zyg-9.2]) II; kss-3.1-3.2(abu522[g.11323197_11326206del]) II; kss-3.3(abu462[p.S99*]) II*	*kss-3.1, kss-3.2, kss-3.3* knockout, N-terminal mScarlet tagged ZYG-9.2, EG6180 background
**INK1172**	*kss-3.1(-) kss-3.2(-) kss-3.4(-)*	*kss-3.1-3.2(abu525[g.11323197_11326206del]) II; kss-3.4(abu460[p.W100*]) II;zyg-9.2(abu458[mScarlet:zyg-9.2]) II*	Knockout of *hyde-1,* knockout of *kss-3.1 to kss-3.4,* C-terminal mScarlet tagged FARS-3, EG6180 background
**INK1196**	*kss-3.1(-) kss-3.4(-)*	*kss-3.1(abu546[g.11323196_11324454del]) II; kss-3.4(abu460[p.W100*]) II; zyg-9.2(abu458[mScarlet:zyg-9.2]) II*	*kss-3.1* and *kss-3.4* knockout, N-terminal mScarlet tagged ZYG-9.2, EG6180 background
**INK1255**	PZL-1::mScarlet	*pzl-1(abu269[pzl-1::mScarlet]) II; qqIR45*	C-terminal mScarlet tagged PZL-1, GGGS linker, QX2341 background

Supplementary Table 3. Figures and corresponding strains used in this figure.

Figure	Strains used
**Fig. 1b**	EG6180, INK169, INK1255
**Fig. 1g**	EG6180, INK318, INK680
**Fig. 1h**	EG6180, INK318, INK680
**Fig. 2b**	EG6180, INK318, INK438, INK512, INK586, INK629, INK680, INK726, INK732
**Fig. 2h**	QX2343, INK563
**Fig. 2i**	INK169, INK206
**Fig. 2j**	INK169, INK206
**Fig. 2k**	INK1030, INK1053
**Fig. 3a**	EG6180, INK793, INK801, INK950, INK951, INK957, INK1169, INK1172, INK1196
**Fig. 3b**	EG6180, INK950
**Fig. 3c**	INK950, INK996, INK1011, EG6180, INK1091, INK1092, INK1095, INK1097, INK1100, INK1164
**Fig. 3d**	EG6180, INK1095
**Fig. 4d**	EG6180, QX2343, INK563, INK631, INK777, INK783
**Fig. 4f**	INK310, INK336, INK767, INK806, INK808
**Fig. 4g**	INK336, INK767, INK854, INK874, INK892, INK894
**Ext. data Fig. 1b**	INK303, INK422
**Ext. data Fig. 1c**	QX2343, EG6180, INK303, INK422
**Ext. data Fig. 1e**	INK324, INK485
**Ext. data Fig. 1f**	QX2341, EG6180, INK324, INK485
**Ext. data Fig. 1g**	QX2343, QX2341, INK66, INK577
**Ext. data Fig. 3f**	QX2341, INK976
**Ext. data Fig. 4a**	INK169, INK206, INK785
**Ext. data Fig. 4b**	EG6180, INK169

**Ext. data Fig. 4c**	INK169, INK206
**Ext. data Fig. 4d**	INK563, INK631
**Ext. data Fig. 4e**	EG6180, INK505, INK563
**Ext. data Fig. 4f**	INK444
**Ext. data Fig. 4g**	INK169, INK206
**Ext. data Fig. 5e**	INK722, INK950, INK919, INK921
**Ext. data Fig. 6a**	INK1164
**Ext. data Fig. 6b**	EG6180, INK1092, INK1164
**Ext. data Fig. 6c**	EG6180, INK1095
**Ext. data Fig. 6h**	INK1064, INK1092
**Ext. data Fig 8a**	EG6180, INK505, INK563, INK777, INK806
**Ext. data Fig 8d**	QX2341, INK590
**Ext. data Fig 8h**	EG6180, INK318, INK322, INK336
**Ext. data Fig 8j**	QX2343, INK63, INK66, INK277, INK581
**Ext. data Fig 8k**	EG6180, INK63, INK66, INK277, INK581
**Ext. data Fig 8l**	INK66, INK277, INK581

Supplementary Table 4. List of gRNAs and primers used in this study.

Lines generated	Modification	gRNA sequence 5'-3'	Repair template sequence	Genotyping primer - 1	Genotyping primer - 2	Amplicon size, bp
**INK303**	*klmt-1* knock-out	CCGTCTCTCCGTGCTTCTTG	no	TGCAGCTTGAAAATGATGAAA	CGATTACCGGGTAGACAGGA	707
**INK63**	*3xFLAG::klmt-1*	CTGATTTTCGGACATTTCTC	tttgtttttccgtggtttttctttgctattttcgaatttaaattaaaaatacggctaataatctcaatattccagagaaatggattacaaagaccatgatggtgactataaggatcatgatattgactataaagaccatgactccgaaaatcagcgactttcgaacaacggttctgtagaag	ATCGCATGCGCCTTAAATACCG	TTTTCGTATTCGTCCGGAGCCT	605
**INK66**	*klmt-1::3xFLAG*	GAATGTATTTAACTTTCCAT	tcagtgacagtgagacggagaaagccgccgacgaacaatccgtggagaaaaagagaaactcctcgtcacagaatccaatggaaagtgattacaaagaccatgatggtgactataaggatcatgatattgactataaagaccatgactaaatacattctttccccccttttccccgcttcatcaatg	ACTGCCAAAGTGCTCACCCTAA	GGGCTTAGGCGGCAAATTAAAT	542
**INK169**	*klmt-1::mNeonGreen*	GAATGTATTTAACTTTCCAT	acgactcactatagggcgaattggccaagaactccttttcgacctcaaacacgtcacctttgatttgaagaatgaaaaaccaactcctagaattttcatcttgagaaaatttggtttgaaaataaaatacggatttcaggagccaccaaacatcacaaattttaatcggatctcctcaacgctttcgaagcttctggaactcgatgaaactaatccggacagtgtcttctcgagaaaacactcttccgaactgaagaaagtgactgccaaagtgctcaccctaatgaaaggagaagtcaaaagagctgagaagaatttgttaatgattcagagcaaagttggattggagatgaggtattttcgcacttcgcaacaaaacagcttcattttcgatttttcagtgacagtgagacggagaaagccgccgacgaacaatccgtggagaaaaagagaaactcctcgtcacagaatccaatggaaagtggtaccgtctccaagggagaggaggacaacatggcctccctcccagccacccacgagctccacatcttcggatccatcaacggagtcgacttcgacatggtcggacaaggaaccggaaacccaaacgacggatacgaggagctcaacctcaagtccaccaaggtaagtttaaacatatatatactaactaaccctgattatttaaattttcagggagacctccaattctccccatggatcctcgtcccacacatcggatacggattccaccaatacctcccatacccagacggaatgtccccattccaagccgccatggtcgacggatccggataccaagtccaccgtaccatgcaattcgaggacggagcctccctcaccgtcaactaccgttacacctacgagggatcccacatcaaggtaagtttaaacagttcggtactaactaaccatacatatttaaattttcagggagaggcccaagtcaagggaaccggattcccagccgacggaccagtcatgaccaactccctcaccgccgccgactggtgccgttccaagaagacctacccaaacgacaaggtaagtttaaacatgattttactaactaactaatctgatttaaattttcagaccatcatctccaccttcaagtggtcctacaccaccggaaacggaaagcgttaccgttccaccgcccgtaccacctacaccttcgccaagccaatggccgccaactacctcaagaaccaaccaatgtacgtcttccgtaagaccgagctcaagcactccaagaccgagctcaacttcaaggagtggcaaaaggccttcaccgacgtcatgggaatggacgagctctacaagtaaatacattctttccccccttttccccgcttcatcaatgtttcgaagaaaacgtttcattgtgttgtttgtgatgagatttggtttgtttttcagtgccactctttcagtggcaagtatataaattgtaatgtttttatttaaaaattatctctatatttatatttgccaaaaaatctggagtttaactgaaaaaacgcgcgtaaactccagatttttcttttttttttgacctgaaaatttaatttgccgcctaagccccccttccccttcacttttctcgatttcccccatttccccttttgttcccactcaattttcctgattcgaaaccctaatccgaaatagcacggggattacgttatcgagtctctttttttgaactcggcgcatagagctcagttcaaaaattatgccatgttttggataactttgcgtttttttgattcccccaccctttcatcgtcatcccgtcatttttccagacgcggcgcaacgatttgagagtattgagcgagaaaaggaagaaaaatgctccgggaatcccaatggcttccgtggggcgtggtcaaaaaccacccgaaatgagctgttccctttagtgagggttaattgc	ACTGCCAAAGTGCTCACCCTAA	GGGCTTAGGCGGCAAATTAAAT	1346
	klmt-1::2xTy1::mNeonGreen	GAGACGGTACCACTTTCCAT	gccgacgaacaatccgtggagaaaaagagaaactcctcgtcacagaatccaatggaaagtggtgaagtgcataccaatcaggacccgctggatgaagtccacacaaaccaagatccactcgatggtaccgtctccaagggagaggaggacaacatggcctccctcccagccacccacgag	ACTGCCAAAGTGCTCACCCTAA	GGGCTTAGGCGGCAAATTAAAT	1409
**INK277**	*ΔN-klmt-1::3xFLAG* (p.S2_T45del)	CTGATTTTCGGACATTTCTC + CCTTCCTCCAACTACTCCAG	gtttttccgtggtttttctttgctattttcgaatttaaattaaaaatacggctaataatctcaatattccagagaaatgactccagtggtacgccattacgtttcttttatttttttatattaaaaaataacaatttaaacgacttttcgccagaaaaaccacattttcg	ATCGCATGCGCCTTAAATACCG	GATCCTTTTCCACCGCCGTTTT	768

**INK581**	*3xFLAG::klmt-1-ΔC* (p.A460_S503del)	GCAAAGTTGGATTGGAGATG + GAATGTATTTAACTTTCCAT	ctgaagaaagtgactgccaaagtgctcaccctaatgaaaggagaagtcaaaagagctgagaagaatttgttaatgattcagagcaaagtttaaatacattctttccccccttttccccgcttcatcaatgtttcgaagaaaacgtttcattgtgttgtttgtgatgagatttggtttg	ACTGCCAAAGTGCTCACCCTAA	GGGCTTAGGCGGCAAATTAAAT	302
**INK422**	*kss-1* knock-out	GAAAAATAGATTCACTACGA	no	ACGGTGTTTCAGGATAGACGAGA	TGTGCCGCCTCTCCTGAATTTA	852
**INK206**	*kss-1* knock-out	ACGTCTGGGAGTCGAATAAG	no	TATCCCATCTGCCACGTGTTGA	TACCGATGAAAGAGGTGTCGCC	306
**INK444**	*3xFLAG::kss-1*	ACGTCTGGGAGTCGAATCAA	cgaacaaccaccaattattgatttttagatctcaataaatctttggattttaatatggactacaaagatcatgacggtgactataaagatcatgacatcgattacaaggatgacgatgacaaggagtttccattgattcgactcccagacgttccaaaaattcaaattatccaattgatgtctattgg	TATCCCATCTGCCACGTGTTGA	TACCGATGAAAGAGGTGTCGCC	415
**INK324**	*pzl-1* knock-out	ACATGGGTTGCCACGGAACT	ctgcaaaagataattcggaacgacgccaacatattttgtcaagtgttggcaatcagaagcgttacatgagtaactgagtcgactcggcgcggaattctccaaattctctgtttctcttctaccagatctgttggaaaagatgaaagag	AACAATGAAATGGCAGGAGCGG	TCTCTCCAATTGCATCGCCTCA	434
**INK485**	*kss-2* knock-out	GAAGATGGAGTATAGTAAGC	ggagacgacgagtggagagtgtttctgggtccttcgaagatggagtaagtagctagccggataatatctcagaggaggatgtgaaaccatggtgagttcc	ATTCGACTGCCAGATGTTCCCA	*TTGCGACACAGGGATAACTCAGT*	521
**INK505**	*fars-3::3xFLAG*	ATAACAATAATTTAGAGGAA	cctctttcggactcacccttccatgtggagccgtggaattcaacgtcgaaccgttcttggattacaaagaccatgatggtgactataaggatcatgatattgactataaagatgacgatgacaagtaaattattgttattgaatgttttctatggaattgttgtttttaaatttaaatattcattcc	ATTCCATTGATCTCACCCCCTGG	TTCTGGCTGGGAAAAAGGGTTCT	711
**INK590**	*kss-2* (p.G297R)	AACCAACTTTTGATGACGGT	ccagagcatcgaatacaactgagaaccaacttttgatgaccgtgagattggtatcttatcctgccgagccaggacgacgagagctcgagttctacg	TGTTCTCAAGGGTTTCCGATCCA	ACCCTCTTCTCATCCAGCCAAC	334
**INK722**	*fars-3::mScarlet*	ATAACAATAATTTAGAGGAA	cccttccatgtggagccgtggaattcaacgtggaaccgttcctcggaggtggatcaatggtctcgaaaggagaggccgtcatcaaggagttcatgcgtttcaaggtccacatggagggatccatgaacggacacgagttcgagatcgagggagagggagagggacgtccatacgagggaacccaaaccgccaagctcaaggtcaccaaggtaagtttaaacatatatatactaactaaccctgattatttaaattttcagggaggaccactcccattctcctgggacatcctctccccacaattcatgtacggatcccgtgccttcatcaagcacccagccgacatcccagactactacaagcaatccttcccagagggattcaagtgggagcgtgtcatgaacttcgaggacggaggagccgtcaccgtcacccaagacacctccctcgaggacggaaccctcatctacaaggtaagtttaaacagttcggtactaactaaccatacatatttaaattttcaggtcaagctccgtggaaccaacttcccaccagacggaccagtcatgcaaaagaagaccatgggatgggaggcctccaccgagcgtctctacccagaggacggagtcctcaagggagacatcaagatggccctccgtctcaaggacggaggacgttacctcgccgacttcaaggtaagtttaaacatgattttactaactaactaatctgatttaaattttcagaccacctacaaggccaagaagccagtccaaatgccaggagcctacaacgtcgaccgtaagctcgacatcacctcccacaacgaggactacaccgtcgtcgagcaatacgagcgttccgagggacgtcactccaccggaggaatggacgaactctacaagtaaattattgttattgaatgttttctatggaattgttgtttttaaatttaaatattc	ATTCCATTGATCTCACCCCCTGG	TTCTGGCTGGGAAAAAGGGTTCT	1506
**INK793**	*kss-3.4* knock-out	GGATAAACGATGAGTGGACAATG	caacaaaattcagtaaaaaaatacggtgttttaggataaacgatgagtaagtgactaagcttagagaaaactttcaatgtgttcatacgtcttcaaaaagttcttcc	CGATGGGCAAAATAGTGCGGTT	GCTCAGCGAAACATTCTGCACT	886

**INK801**	*kss-3.3* knock-out	AGGATAGACGAGAAGAGCGCA	caacaaaattcagtaaaagaatacatgatttcaggatagacgagaagtgactaagctttgatagagagaactttcaatgtgttcatacgtcttcaaaaagttcttcc	GAGAACTATCTGGCGTGGGTGT	AGTCAAGCTCGTTTAGTATTTGGGA	1025
**INK951**	*kss-3.1; kss-3.2* double knock-out	ACTGTGCAATCCACATCGAACA + GAAACACAACAACATTCGAGTT	ccagaatcaaactggctctttcctctcgaaaaatggagaactacttgtcatgggtgttcaaaaatcactaaatgtcctgtttcgaaaagtgaaggtacgaacgagatcaaaggggttcgagatgtccgggagcc	TTTGCCCATCGAACGACTACCT	GTGGGAAGGGGATATGACTGAGC	1126
**INK1064**	*klmt-1* knock-out	CCGTCTCTCCGTGCTTCTTG	no	TGCAGCTTGAAAATGATGAAA	CGATTACCGGGTAGACAGGA	707
	NIC-ORF019770 knock-out	TGAAGCGACTCCAATCACAA	no	TGAATATGCCGACCCGTGAGTT	ATTCTCAGCTGGTTCGAACGGA	361
	pzl-1 knock-out	TGGCACAATTGGAATGGAGT	cgttgaacgaggagttgattcagtggcaaatccatggacagtggcacaagatgatatggcataactgactaagcttggaatatggctcgtgattcgaacactgatagattctactcaacctcatgggattcaactg	AGCCGCTTATTTGAAAATCCGCT	ACTGTGTTCAGCTACGATTCCGA	733
	slow-1 knock-out	AATGAAGCTCAAACCACACG	ggttgtccgaagacgattttatacaaaaatgaagctcaatgactaactgaggagacttccgagaactctgtagcgcgcgaagagtaag	GCTCAATCGCCTATTGGAGGGA	TCCGATTTGCATTGATCCCTTTGG	567
**INK1088**	*mScarlet::zyg-9.2*	AGTCAGTCAAAAAATGTCGAAT	cctctttcacccctaaaaccctctttttcagtcagtcaaaaaatggtctcgaaaggagaggccgtcatcaaggagttcatgcgtttcaaggtccacatggagggatccatgaacggacacgagttcgagatcgagggagagggagagggacgtccatacgagggaacccaaaccgccaagctcaaggtcaccaaggtaagtttaaacatatatatactaactaaccctgattatttaaattttcagggaggaccactcccattctcctgggacatcctctccccacaattcatgtacggatcccgtgccttcatcaagcacccagccgacatcccagactactacaagcaatccttcccagagggattcaagtgggagcgtgtcatgaacttcgaggacggaggagccgtcaccgtcacccaagacacctccctcgaggacggaaccctcatctacaaggtaagtttaaacagttcggtactaactaaccatacatatttaaattttcaggtcaagctccgtggaaccaacttcccaccagacggaccagtcatgcaaaagaagaccatgggatgggaggcctccaccgagcgtctctacccagaggacggagtcctcaagggagacatcaagatggccctccgtctcaaggacggaggacgttacctcgccgacttcaaggtaagtttaaacatgattttactaactaactaatctgatttaaattttcagaccacctacaaggccaagaagccagtccaaatgccaggagcctacaacgtcgaccgtaagctcgacatcacctcccacaacgaggactacaccgtcgtcgagcaatacgagcgttccgagggacgtcactccaccggaggaatggacgagctttataagggtggatcgaattgggactatatagacgaagtggacattattccaaagcttcc	CGCTTCATTCGTTCATAAAAACCTGG	AGCCCTGTAGCGAATTTAGCGA	1430
**INK1164**	*hyde-1* knock-out	TGCCCGCATTTCATTGCGATCT + CGAGCCATGCCACAGCTCCCT	cgactgagatgatgcagtacttcagcggcttctctaataatttccgagccatgactaactgaaatgcgggcacaaagtaattgtgacgtggcccactcctcctggagcgc	GTAGTGACCCGACAGAAGCTGAT	TGAAAAAGGAGCTTTGAAGGCCG	599
**INK1193**	*kss-3.1* knock-out	ACAATCTATCTGCTGAGTGAAT + GAAACACAACAACATTCGAGTT	gtcatttgagattatgacgacaagacagataccatctgccacgtcatttcacaatctatctgcaaatgtcctgtttcgaaaagtgaaggtacgaacgagatcaaaggggttcgagatgtccgggagcc	ACGGAGGTTAAGTTTTTAGCGCTT	GTGGGAAGGGGATATGACTGAGC	1106

**INK1255**	*pzl-1::mScarlet*	CGGAGGAC GACTAATT TATTA	ggttgccgccttcgagatcaaaatctttacggaggacgacggaggtggatcaatggtctcgaaaggagaggccgtcatcaaggagttcatgcgtttcaa ggtccacatggagggatccatgaacggacacgagttcgagatcgagggagagggagagggacgtccatacgagggaacccaaaccgccaagctc aaggtcaccaaggtaagtttaaacatatatatactaactaaccctgattatttaaattttcagggaggaccactcccattctcctgggacatcctctccccac aattcatgtacggatcccgtgccttcatcaagcacccagccgacatcccagactactacaagcaatccttcccagagggattcaagtgggagcgtgtcat gaacttcgaggacggaggagccgtcaccgtcacccaagacacctccctcgaggacggaaccctcatctacaaggtaagtttaaacagttcggtactaa ctaaccatacatatttaaattttcaggtcaagctccgtggaaccaacttcccaccagacggaccagtcatgcaaaagaagaccatgggatgggaggcct ccaccgagcgtctctacccagaggacggagtcctcaagggagacatcaagatggccctccgtctcaaggacggaggacgttacctcgccgacttcaa ggtaagtttaaacatgattttactaactaactaatctgatttaaattttcagaccacctacaaggccaagaagccagtccaaatgccaggagcctacaacgt cgaccgtaagctcgacatcacctcccacaacgaggactacaccgtcgtcgagcaatacgagcgttccgagggacgtcactccaccggaggaatgga cgaactctacaagtaatttattattttttaattcgttattcaattcattattttgacccccacaacc	AACCTTCT CGCCTGGA CACTAC	ATCGATGGA GGCTTGGAA GGAG	1225

Supplementary Table 5. List of repair templates and primers used for synthetic landing pad injections. Same gRNA was used for all SLP injections: 5’-AAAGTCCACAATCTCCACGT-3’. Amplicon size for empty Chr. I SLP - 2007bp. For empty Chr. IV SLP - 2236bp.

Lines generated	Insertion (short name)	Transgene inserted	Background	Repair template sequence (plasmid)	Genotyping primer - 1	Genotyping primer - 2	Genotyping primer - 3	Amplicon size 1+2 (external), bp	Amplicon size 1+3 (internal), bp
**INK310**	heat-shock inducible mCherry	*hsp-16.11p::mCherry::tbb-2 3' UTR + HygR(+)*	Chr. IV SLP	pAB0174	TCCAATCTCGCTCTTCAACTCGT	TGTTCGCCGTACAGAGAACATCT	TTTTGCGGTTTGTGTTCCCT	4966	1591
**INK318**	heat-shock inducible KLMT-1	*hsp-16.11p::klmt-1::tbb-2 3' UTR + HygR(+)*	Chr. IV SLP	pAB0176	TCCAATCTCGCTCTTCAACTCGT	TGTTCGCCGTACAGAGAACATCT	TGGAGGAAGGTCTGGAGCATTG	5614	1573
**INK322**	heat-shock inducible ΔNC-KLMT-1	*abuSi34[hsp-16.11p::klmt-1(p.1M; p.K70_R459)::tbb-2 3' UTR + HygR(+); abuSi9] IV*	Chr. IV SLP	pAB0185	TCCAATCTCGCTCTTCAACTCGT	TGTTCGCCGTACAGAGAACATCT	TTGCCCGTCTCCTGGTTATCAG	5278	1749
**INK336**	heat-shock inducible IDR-mCherry-IDR	*hsp-16.11p::KLMT-1(p.M1_D69)::mCherry::KLMT-1(p.A460_S503)::tbb-2 3' UTR + HygR(+)*	Chr. IV SLP	pAB0192	TCCAATCTCGCTCTTCAACTCGT	TGTTCGCCGTACAGAGAACATCT	TGGAGGAAGGTCTGGAGCATTG	5305	1573
**INK438**	heat-shock inducible KSS-1	*hsp-16.11p::kss-1::tbb-2 3' UTR + HygR(+)*	Chr. I SLP	pAB0260	TGAGATGATTGATGAGGCGTCAAG	TGAAGAGAAAAAGGGCATGGTCA	TACCGATGAAAGAGGTGTCGCC	5285	1588
**INK563**	3xFLAG::KSS-1 *(Ctr-rpl-36p)*	*rpl-36p::3xFLAG::kss-1::rpl-36 3' UTR + HygR(+)*	Chr. I SLP	pAB0293	TGAGATGATTGATGAGGCGTCAAG	TGAAGAGAAAAAGGGCATGGTCA	TACCGATGAAAGAGGTGTCGCC	4864	1419
**INK586**	heat-shock inducible KSS-2	*hsp-16.11p::kss-2::tbb-2 3' UTR + HygR(+)*	Chr. I SLP	pAB0294	TGAGATGATTGATGAGGCGTCAAG	TGAAGAGAAAAAGGGCATGGTCA	TTGCGACACAGGGATAACTCAGT	5955	1881
**INK680**	heat-shock inducible PZL-1	*hsp-16.11p::pzl-1::tbb-2 3' UTR + HygR(+)*	Chr. IV SLP	pAB0336	TCCAATCTCGCTCTTCAACTCGT	TGTTCGCCGTACAGAGAACATCT	ACTGTGTTCAGCTACGATTCCGA	6469	2062

**INK777**	3xFLAG::KSS-1(INDDTL) *(Ctr-rpl-36p)*	*rpl-36p::3xFLAG::kss-1(R323_V328delinsINDDTL)::rpl-36 3' UTR + HygR(+)*	Chr. I SLP	pAB0332	TGAGATGATTGATGAGGCGTCAAG	TGAAGAGAAAAAGGGCATGGTCA	TACCGATGAAAGAGGTGTCGCC	4864	1419
**INK854**	heat-shock inducible mCherry-IDR	*hsp-16.11p::mCherry::KLMT-1(p.A460_S503)::tbb-2 3' UTR + HygR(+)*	Chr. IV SLP	pAB0382	TCCAATCTCGCTCTTCAACTCGT	TGTTCGCCGTACAGAGAACATCT	TTTTGCGGTTTGTGTTCCCT	5098	1591
**INK874**	heat-shock inducible IDR-mCherry	*hsp::KLMT-1(p.M1_D69)::mCherry::tbb-2 3' UTR + HygR(+)*	Chr. IV SLP	pAB0381	TCCAATCTCGCTCTTCAACTCGT	TGTTCGCCGTACAGAGAACATCT	TGGAGGAAGGTCTGGAGCATTG	5173	1573
**INK877**	heat-shock inducible KSS-3.1	*hsp-16.11p::kss-3.1::tbb-2 3' UTR + HygR(+)*	Chr. I SLP	pAB0346	TGAGATGATTGATGAGGCGTCAAG	TGAAGAGAAAAAGGGCATGGTCA	TGGGGAGATCTGGCAGTTGGA	5457	1384
**INK880**	heat-shock inducible KSS-3.3	*hsp-16.11p::kss-3.3::tbb-2 3' UTR + HygR(+)*	Chr. I SLP	pAB0347	TGAGATGATTGATGAGGCGTCAAG	TGAAGAGAAAAAGGGCATGGTCA	ACACCTGTCAAGTTCGCGAC	5286	1891
**INK976**	3xFLAG::KSS-2 *(Ctr-rpl-36p)*	*rpl-36p::3xFLAG::kss-2::rpl-36 3' UTR + HygR(+)*	Chr. I SLP	pAB0372	TGAGATGATTGATGAGGCGTCAAG	TGAAGAGAAAAAGGGCATGGTCA	TTGCGACACAGGGATAACTCAGT	5537	1715
**INK1030**	heat-shock inducible PZL-1::mCherry	*hsp-16.11p::pzl-1::mCherry::tbb-2 3' UTR + HygR(+)*	Chr. IV SLP	pAB0407	TCCAATCTCGCTCTTCAACTCGT	TGTTCGCCGTACAGAGAACATCT	ACTGTGTTCAGCTACGATTCCGA	7342	2062

Supplementary Table 6. Raw data for KLMT-1 injection experiments into adult gonads. Co-injection marker helps to visually distinguish the offspring that received an injection dose.

Worm number	Injection mix	Strain	Total number of eggs	With co-injection marker				Without co-injection marker			
				Total	Affected	WT	% affected	Total	Affected	WT	% affected
1	Buffer	EG6180	136	38	0	38	0,0	98	0	98	0,0
2	Buffer	EG6180	110	36	1	35	2,8	74	1	73	1,4
3	Buffer	EG6180	118	49	1	48	2,0	69	0	69	0,0
4	Buffer	EG6180	131	37	1	36	2,7	94	0	94	0,0
5	Buffer	EG6180	137	31	0	31	0,0	106	8	98	7,5
6	Buffer	EG6180	110	40	0	40	0,0	70	2	68	2,9
7	Buffer	EG6180	109	38	0	38	0,0	71	0	71	0,0
8	Buffer	EG6180	65	24	0	24	0,0	41	7	34	17,1
9	Buffer	EG6180	138	42	1	41	2,4	96	2	94	2,1
10	Buffer	EG6180	119	33	0	33	0,0	86	1	85	1,2
11	Buffer	EG6180	131	23	0	23	0,0	108	3	105	2,8
12	Buffer	EG6180	117	19	0	19	0,0	98	3	95	3,1
13	Buffer	EG6180	189	26	0	26	0,0	163	2	161	1,2
14	KLMT-1	EG6180	96	28	19	9	67,9	68	7	61	10,3
15	KLMT-1	EG6180	116	41	19	22	46,3	75	8	67	10,7
16	KLMT-1	EG6180	118	45	36	9	80,0	73	1	72	1,4
17	KLMT-1	EG6180	133	34	30	4	88,2	99	1	98	1,0
18	KLMT-1	EG6180	102	32	27	5	84,4	70	5	65	7,1
19	KLMT-1	EG6180	110	52	44	8	84,6	58	3	55	5,2
20	KLMT-1	EG6180	141	40	36	4	90,0	101	3	98	3,0
21	KLMT-1	EG6180	150	31	25	6	80,6	119	5	114	4,2
22	KLMT-1	EG6180	69	36	32	4	88,9	33	4	29	12,1
23	KLMT-1	EG6180	89	26	21	5	80,8	63	2	61	3,2
24	KLMT-1	EG6180	123	28	23	5	82,1	95	9	86	9,5
25	KLMT-1	EG6180	176	39	29	10	74,4	137	8	129	5,8
26	KLMT-1	EG6180	145	42	32	10	76,2	103	5	98	4,9
27	KLMT-1	EG6180	116	27	19	8	70,4	89	6	83	6,7
28	KLMT-1	EG6180	136	18	13	5	72,2	118	3	115	2,5
29	KLMT-1	EG6180	154	39	30	9	76,9	115	8	107	7,0
30	KLMT-1	EG6180	165	32	25	7	78,1	133	8	125	6,0
31	KLMT-1	EG6180	134	38	28	10	73,7	96	10	86	10,4
32	KLMT-1	INK563 (*kss-1* OE)	101	42	0	42	0,0	59	3	56	5,1
33	KLMT-1	INK563 (*kss-1* OE)	103	10	6	4	60,0	93	2	91	2,2
34	KLMT-1	INK563 (*kss-1* OE)	153	39	0	39	0,0	114	1	113	0,9
35	KLMT-1	INK563 (*kss-1* OE)	101	35	0	35	0,0	66	1	65	1,5
36	KLMT-1	INK563 (*kss-1* OE)	150	23	0	23	0,0	127	1	126	0,8
37	KLMT-1	INK563 (*kss-1* OE)	154	44	1	43	2,3	110	1	109	0,9
38	KLMT-1	INK563 (*kss-1* OE)	3	0	0	0	0,0	3	0	3	0,0
39	KLMT-1	INK563 (*kss-1* OE)	128	46	0	46	0,0	82	3	79	3,7

Supplementary References

1. Seydoux, G. & Fire, A. Soma-germline asymmetry in the distributions of embryonic RNAs in Caenorhabditis elegans. *Development* **120**, 2823–2834 (1994).
2. Kawasaki, I. *et al.* The PGL Family Proteins Associate With Germ Granules and Function Redundantly in Caenorhabditis elegans Germline Development. *Genetics* **167**, 645–661 (2004).
3. Raj, A., van den Bogaard, P., Rifkin, S. A., van Oudenaarden, A. & Tyagi, S. Imaging individual mRNA molecules using multiple singly labeled probes. *Nat Methods* **5**, 877–879 (2008).
