# Supplementary material for: Recurrent evolution of selfishness from an essential tRNA synthetase in Caenorhabditis tropicalis
Source: Nat Ecol Evol. 2025 Nov 17;9(12):2374–90. doi: 10.1038/s41559-025-02894-2 (PMC12680543; doi:10.1038/s41559-025-02894-2)

Fig. 2f and 2g. Uncropped Coomassie-stained gels

Used region is marked with a red dashed outline. Other lanes are not relevant to the final image.

For KLMT-1–KSS-1–SKR-1 complex (Fig. 2f)

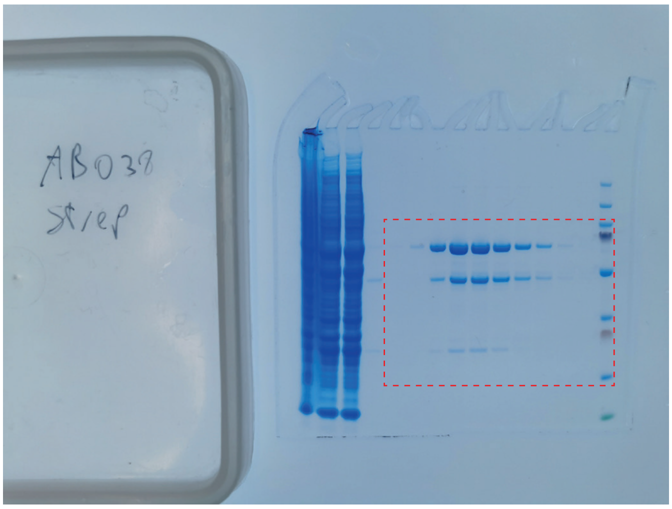

For KSS-1–SKR-1 complex (Fig. 2g)

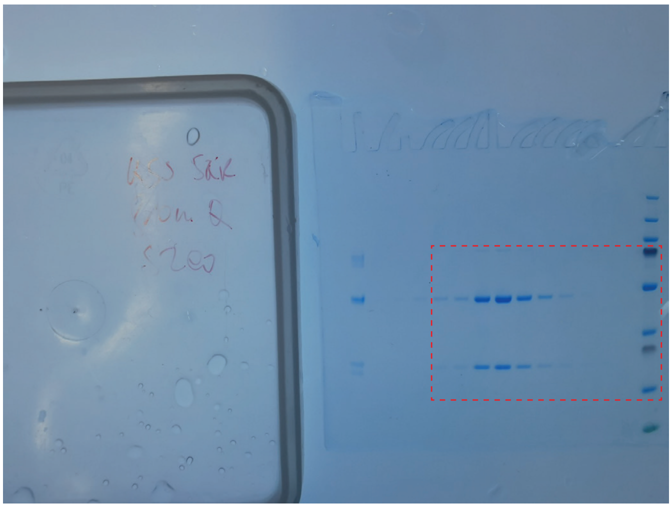

Supplement: Supplementary file 14 — Unmodified Coomassie-stained gels. [file 41559_2025_2894_MOESM14_ESM.pdf]
