# Supplementary material for: Recurrent evolution of selfishness from an essential tRNA synthetase in Caenorhabditis tropicalis
Source: Nat Ecol Evol. 2025 Nov 17;9(12):2374–90. doi: 10.1038/s41559-025-02894-2 (PMC12680543; doi:10.1038/s41559-025-02894-2)
Supplement: Supplementary file 18 — Unmodified western blot membranes. [file 41559_2025_2894_MOESM18_ESM.pdf]

Extended Data Fig. 1g. Uncropped Western blot membranes

KLMT-1::3xFLAG. Membrane was stained with anti-FLAG antibody, followed by HRP-conjugated secondary (top), then stripped and stained with anti-Actin, followed by HRP-conjugated secondary (bottom).

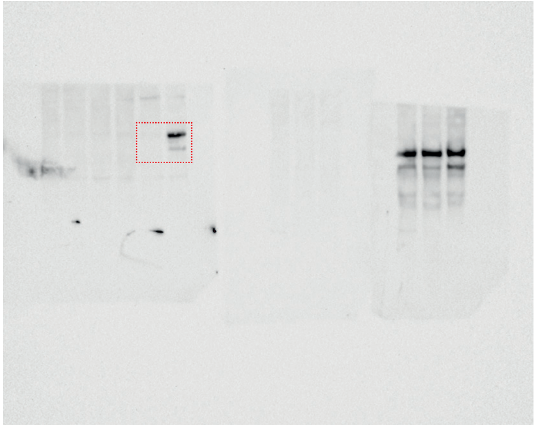

FLAG

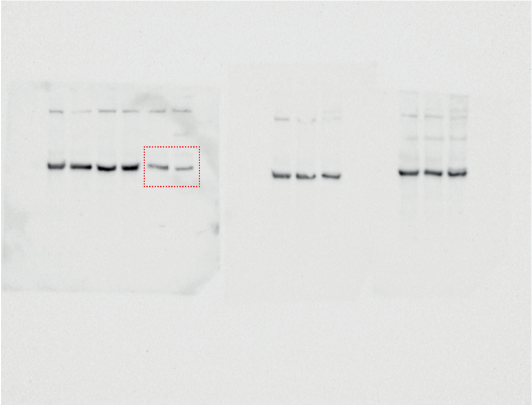

Actin

PZL-1::3xFLAG. Membrane was stained with anti-FLAG antibody (top) and anti-alpha-tubulin antibody at the same time (Alexa647, bottom), followed by HRP-conjugated secondary.

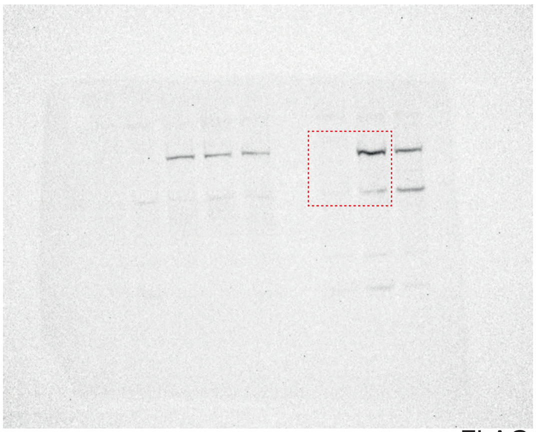

FLAG

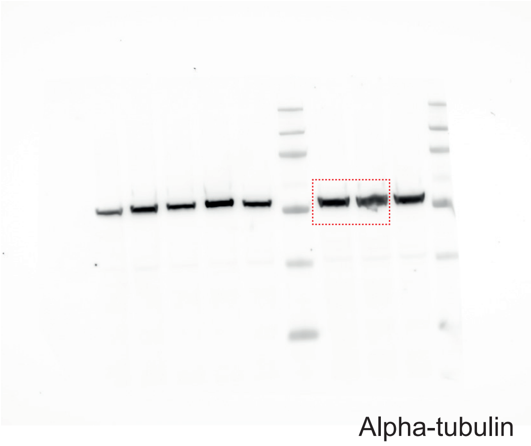

Alpha-tubulin

Regions used for the figure are marked with a red dashed outline. Other lanes and bands are not relevant to the final image.
