# Supplementary material for: Recurrent evolution of selfishness from an essential tRNA synthetase in Caenorhabditis tropicalis
Source: Nat Ecol Evol. 2025 Nov 17;9(12):2374–90. doi: 10.1038/s41559-025-02894-2 (PMC12680543; doi:10.1038/s41559-025-02894-2)
Supplement: Supplementary file 20 — Unmodified western blot membranes. [file 41559_2025_2894_MOESM20_ESM.pdf]

Extended Data Fig. 4e and 4f uncropped Western blot membranes

3xFLAG::KSS-1 Western (Ext. Data 4e)
Same membrane, but different region is shown in Ext. Data 8a.

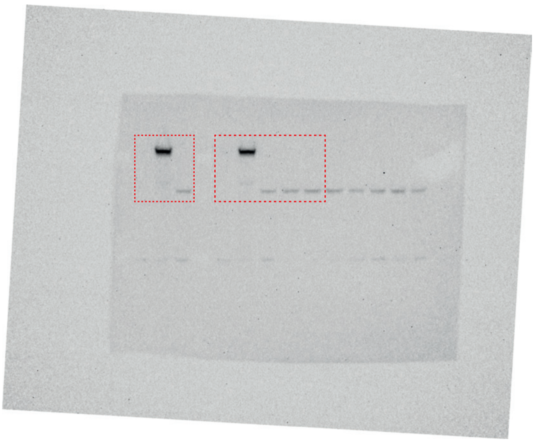

FLAG

Endogenous 3xFLAG::KSS-1
(Ext. Data 4f) - right membrane
Left membrane is shown in Ext. Data 8j

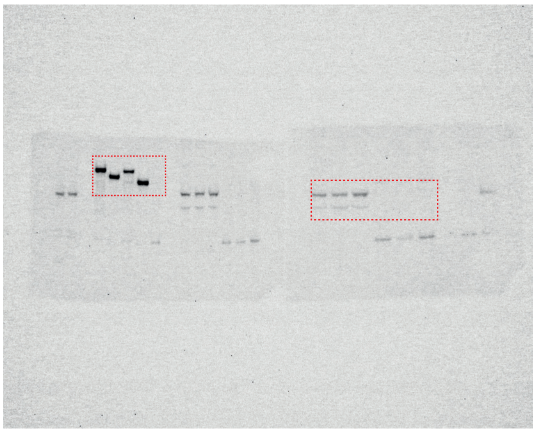

FLAG

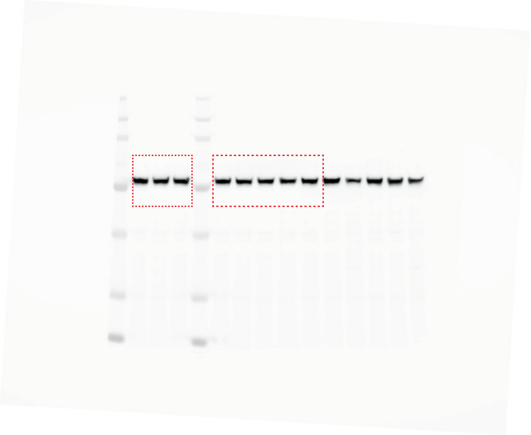

Alpha-tubulin

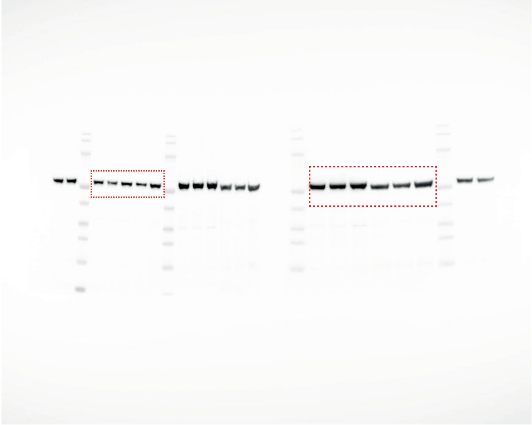

Alpha-tubulin

In both cases the membrane was stained with anti-FLAG and anti-alpha-tubulin antibodies simultaneously. Used regions are marked with a red dashed outline. Other lanes are not relevant to the final image.
