# Supplementary material for: Recurrent evolution of selfishness from an essential tRNA synthetase in Caenorhabditis tropicalis
Source: Nat Ecol Evol. 2025 Nov 17;9(12):2374–90. doi: 10.1038/s41559-025-02894-2 (PMC12680543; doi:10.1038/s41559-025-02894-2)
Supplement: Supplementary file 24 — Unmodified western blot membranes. [file 41559_2025_2894_MOESM24_ESM.pdf]

Extended Data Fig. 8a, 8h and 8j. Uncropped Western blot membranes

Ext. Data 8a - this membrane is shown in Ext. Data 4e, see Tikanova_UnmodifiedBlots_Extended-Fig4.pdf
Ext. Data 8j - see left membrane of Ext. Data 4f source file, Tikanova_UnmodifiedBlots_Extended-Fig4.pdf

KLMT-1 versus KLMT-1 with truncated IDRs (Ext. Data 8j). Each protein sample was split into two and loaded into two gels resulting in two membranes. One was stained with anti-FLAG and another one with anti-Actin antibody.

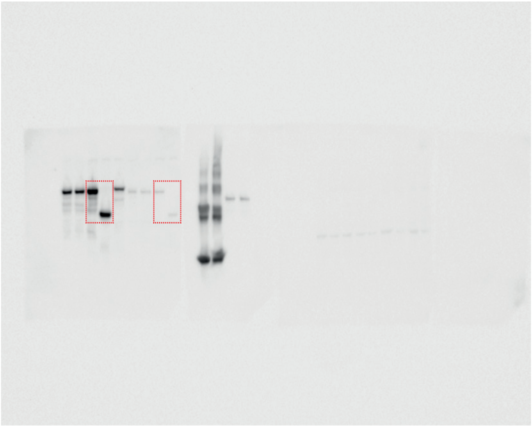

FLAG

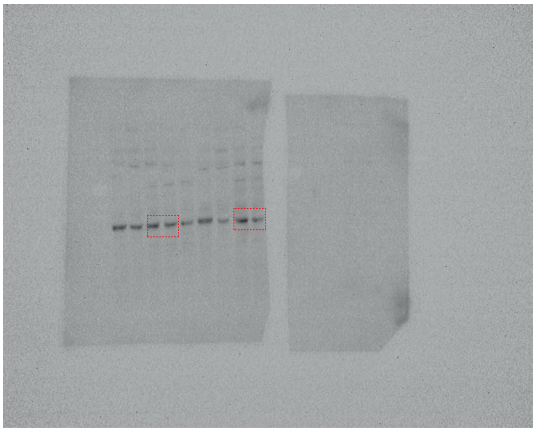

Actin

Used regions are marked with a red dashed outline. Other lanes are not relevant to the final image.
